# Molecular epidemiology of a primarily MSM acute HIV‐1 cohort in Bangkok, Thailand and connections within networks of transmission in Asia

**DOI:** 10.1002/jia2.25204

**Published:** 2018-11-22

**Authors:** David Chang, Eric Sanders‐Buell, Meera Bose, Anne Marie O'Sullivan, Phuc Pham, Eugene Kroon, Donn J Colby, Rujipas Sirijatuphat, Erik Billings, Suteeraporn Pinyakorn, Nitiya Chomchey, Wiriya Rutvisuttinunt, Gustavo Kijak, Mark de Souza, Jean‐Louis Excler, Praphan Phanuphak, Nittaya Phanuphak, Robert J O'Connell, Jerome H Kim, Merlin L Robb, Nelson L Michael, Jintanat Ananworanich, Sodsai Tovanabutra, the RV254/SEARCH 010 Study Group

**Affiliations:** ^1^ United States Military HIV Research Program Walter Reed Army Institute of Research Silver Spring MD USA; ^2^ The Henry M. Jackson Foundation for the Advancement of Military Medicine Bethesda MD USA; ^3^ SEARCH Bangkok Thailand; ^4^ Department of Medicine Faculty of Medicine Siriraj Hospital Mahidol University Bangkok Thailand; ^5^ Department of Retrovirology Armed Forces Research Institute of Medical Sciences Bangkok Thailand; ^6^ Viral Diseases Branch Walter Reed Army Institute of Research Silver Spring MD USA; ^7^ The Thai Red Cross AIDS Research Centre Bangkok Thailand; ^8^ International Vaccine Institute Seoul South Korea; ^9^ Department of Global Health Academic Medical Center University of Amsterdam Amsterdam The Netherlands; ^10^ Present address: GSK Vaccines Rockville MD USA

**Keywords:** HIV‐1 molecular epidemiology, Thailand, Asia transmission network, MSM, acute infection, recombinants, vaccine, intervention

## Abstract

**Introduction:**

Thailand plays a substantial role in global HIV‐1 transmission of CRF01_AE. Worldwide, men who have sex with men (MSM) are at elevated risk for HIV‐1 infection. Hence, understanding HIV‐1 diversity in a primarily Thai MSM cohort with acute infection, and its connections to the broader HIV‐1 transmission network in Asia is crucial for research and development of HIV‐1 vaccines, treatment and cure.

**Methods:**

Subtypes and diversity of infecting viruses from individuals sampled from 2009 to 2015 within the RV254/SEARCH 010 cohort were assessed by multiregion hybridization assay (MHAbce), multiregion subtype‐specific PCR assay (MSSPbce) and full‐length single‐genome sequencing (SGS). Phylogenetic analysis was performed by maximum likelihood. Pairwise genetic distances of envelope gp160 sequences obtained from the cohort and from Asia (Los Alamos National Laboratory HIV Database) were calculated to identify potential transmission networks.

**Results:**

MHAbce/MSSPbce results identified 81.6% CRF01_AE infecting strains in RV254. CRF01_AE/B recombinants and subtype B were found at 7.3% and 2.8% respectively. Western subtype B strains outnumbered Thai B′ strains. Phylogenetic analysis revealed one C, one CRF01_AE/CRF02_AG recombinant and one CRF01_AE/B/C recombinant. Asian network analysis identified one hundred and twenty‐three clusters, including five clusters of RV254 participants. None of the RV254 sequences clustered with non‐RV254 sequences. The largest international cluster involved 15 CRF01_AE strains from China and Vietnam. The remaining clusters were mostly intracountry connections, of which 31.7% included Thai nodes and 43.1% included Chinese nodes.

**Conclusion:**

While the majority of strains in Thailand are CRF01_AE and subtype B, emergence of unique recombinant forms (URFs) are found in a moderate fraction of new HIV‐1 infections. Approaches to vaccine design and immunotherapeutics will need to monitor and consider the expanding proportion of recombinants and the increasing genetic diversity in the region. Identified HIV‐1 transmission networks indicate ongoing spread of HIV‐1 among MSM. As HIV‐1 epidemics continue to expand in other Asian countries, transmission network analyses can inform strategies for prevention, intervention, treatment and cure.

## 1 Introduction

Over 30 years have passed since the first case of HIV‐1 was reported in Thailand 1. In the mid‐1980s, cases of HIV‐1 in the country were largely limited to people who inject drugs (PWID), and were of a subtype B variant (termed Thai B′) related to strains in the US and western Europe 2, 3. The 1990s saw the early epidemic spread rapidly within the country to female sex workers (FSW) and their clients, along with an increase in prevalence of CRF01_AE, known then as subtype E. By the mid‐1990s, interventions such as the 100% Condom Use Programme dramatically reduced incident cases among FSW and their clients 4. In the early 2000s, the HIV‐1 epidemic began to mature, and became more prevalent among men who have sex with men (MSM) 5. In recent years, the epidemic appears to be receding due to successful scale up of antiretroviral therapy (ART), as evidenced by declining incidence in the heterosexual population and decreased numbers of new infections and AIDS‐related deaths since 2001 6. As of 2016, 450,000 people were estimated to be living with HIV‐1 in Thailand, with 6400 new infections and 16,000 AIDS‐related deaths 7. In the same year, Thailand became the first country in Asia to have eliminated mother‐to‐child transmission (MTCT) of HIV‐1 8.

Situated at the crossroads of international and intercontinental trade and tourism, Thailand continues to be intertwined in networks of HIV‐1 transmission. As such, the country has served as a long‐standing base for research on HIV‐1 epidemiology, treatment, prevention, vaccines and cure strategies. Analyses for correlates of protection in the landmark HIV vaccine efficacy trial RV144 are still ongoing 9, 10, 11, 12, 13. The increasing prevalence of intersubtype recombinant forms of HIV‐1 has added deeper complexity to the already challenging genetic diversity inherent to the retrovirus 14, 15. Effective preventive vaccines or immunotherapeutics for HIV cure must be able to counter a multitude of genetic forms of circulating HIV‐1 viruses through molecular surveillance of viruses.

Despite numerous efforts to curtail the epidemic, HIV‐1 transmission persists in Thailand, largely affecting MSM and transgender women (TGW) 16, 17. Molecular surveillance of circulating viruses and understanding the underlying dynamics of HIV‐1 transmission networks can help inform strategies for prevention, intervention, treatment, and cure.

In this study, using HIV‐1 full‐genome sequencing, we report molecular epidemiological data primarily from MSM in Bangkok diagnosed with acute HIV infection (AHI) from 2009 to 2015. These participants were enrolled in the RV254 cohort conducted to support vaccine and treatment research 18. For the purpose of vaccine research and development, we also performed phylogenetic analysis using *env* sequences (~2.5 kb) with a stringent genetic distance threshold to provide more resolution in identifying transmission clusters within RV254 and across the Asia region.

## 2 Methods

### 2.1 Samples

Plasma was collected as part of the study protocol RV254 (or SEARCH 010, clinicaltrials.gov NCT00796146), an ongoing prospective, longitudinal cohort study of participants with AHI in Bangkok, Thailand. The protocol was reviewed and approved by the institutional review boards of Chulalongkorn University (Bangkok, Thailand) and the Walter Reed Army Institute of Research (Silver Spring, Maryland, USA). All study participants provided informed consent. Samples used in this study were from the first available time‐point following HIV diagnosis but prior to ART and were collected a median of two days (IQR two to three days) following first documented positive HIV RNA test. Fiebig stages categorized upon enrolment ranged from Fiebig I to V 19. In the present analysis, participant identifiers have been removed to protect privacy.

### 2.2 HIV‐1 subtyping

Viral RNA extracted from plasma samples was screened for subtype reactivity through a multiregion hybridization assay (MHAbce) or multiregion subtype‐specific PCR assay (MSSPbce) as previously described 20, 21. The sample set was subjected to near full‐genome amplification and (Sanger) sequencing, prioritized by chronological sample ID number and other parameters of interest, which included recombinant forms as determined by MHAbce and MSSPbce.

HIV‐1 sequences were obtained either as a near full‐length genome, or as two half genomes overlapping by approximately 1.5 kb, by single‐genome sequencing (SGS) as previously described 22. For samples with low viral load, only the envelope gp160 gene was amplified. For each individual, approximately 10 complete amplicons were generated, and a representative sequence most similar to the aligned consensus was used for analysis (GenBank accession numbers MG989490‐MG989671).

### 2.3 Phylogenetic analysis

Full‐genome subtype and CRF reference sequences were downloaded from the Los Alamos National Laboratory (LANL) HIV Database (Table S1). Alignments of RV254 half‐ and near full‐genome sequences and reference sequences were generated using the MAFFT alignment method in HIVAlign (https://www.hiv.lanl.gov/content/sequence/VIRALIGN/viralign.html), and manually edited using Geneious Pro 5.6.7 (Biomatters Ltd., Auckland, New Zealand) 23. Phylogenetic analyses and pairwise distance calculations were performed via the online Cyberinfrastructure for Phylogenetic Research (CIPRES) Science Gateway (https://www.phylo.org) using maximum likelihood trees reconstructed with RAxML by implementing the general time reversible (GTR+I+G) substitution model with gamma‐distributed rate heterogeneity and a proportion of invariable sites, which was the best model of evolution for the data set as evaluated by ModelFinder (local computing) or jModelTest 2 (CIPRES) 24, 25, 26, 27, 28, 29, 30. The topology of trees was tested by bootstrap analysis with 100 iterations. Phylogenetic trees were plotted in MEGA7, with labels for branch support values ≥70 31. Recombinant breakpoint structures were determined using the jumping profile hidden Markov model (jpHHM, http://jphmm.gobics.de/), visual inspection, confirmed by subgenomic phylogenetic analysis and plotted using RecDraw 32, 33. When a full‐genome sequence for a RV254 participant was not available, representative corresponding half genomes that contained no nucleotide mismatches within their 1.5‐kb overlapping region were patched to obtain a complete genome structure.

### 2.4 Transmission network analysis

Envelope gene (gp160) sequences downloaded from the LANL HIV Database were filtered to include one sequence per individual from the Asia geographic region (as defined by LANL, https://www.hiv.lanl.gov/content/sequence/HelpDocs/geo_regions.html, shown in Table S2). Identical sequences were excluded. Risk factor and country code information were extracted as annotated in the LANL database, and for consistency, MSM and homosexual were preserved as separate risk factors in the present analysis. Sequence alignments and pairwise distance calculations of gp160 from RV254 and Asia were conducted in two steps: sequences were first aligned using the HMM‐align alignment method in HIVAlign, manually refined by visual inspection, and pairwise distance calculations were performed as described above 34. To minimize confounding factors within an alignment of sequences across multiple subtypes and to more accurately calculate pairwise genetic distance, pairs of individual sequences with <10% genetic distance were then aligned pairwise by MAFFT and genetic distance between the pairs was calculated using the Tamura–Nei method 35. Subsequently, a genetic distance threshold of ≤3% was used to identify potential transmission clusters. Network diagrams were plotted using Cytoscape 3.3.0 36. Sankey diagrams were plotted using JavaScript code adapted from the GitHub repository by Sara Quigley (https://github.com/saraquigley/) and based on the D3.js JavaScript library (https://d3js.org) by Mike Bostock.

## 3 Results

The RV254 study population (n = 303) is comprised primarily of males (96.0%), MSM (92.1%), of various occupations from Bangkok. Their socio‐demographic characteristics are presented in Table S3.

### 3.1 HIV‐1 subtype identification

A total of 288 out of 303 samples were successfully screened by MHAbce (n = 63) or MSSPbce (n = 225) for subtype analysis. Viruses were predominantly CRF01_AE (235, 81.6%), followed by CRF01_AE/B recombinants (21, 7.3%), subtype B (8, 2.8%), subtype C (1, 0.3%) and non‐typeable (23, 8.0%).

Subtypes of viruses obtained by SGS from one hundred and thirty‐eight individuals were predominantly CRF01_AE (95, 68.8%), seven of which were classified based on half‐genome sequences only. Nearly a quarter of infections were CRF01_AE/B recombinant (32, 23.2%). There were eight subtype B infections (5.8%), and one each of subtype C, CRF01_AE/B/C recombinant and CRF01_AE/CRF02_AG recombinant (0.7% each). A full list of RV254 sequences included in the present analysis is shown in Table S1.

**Figure 1:**
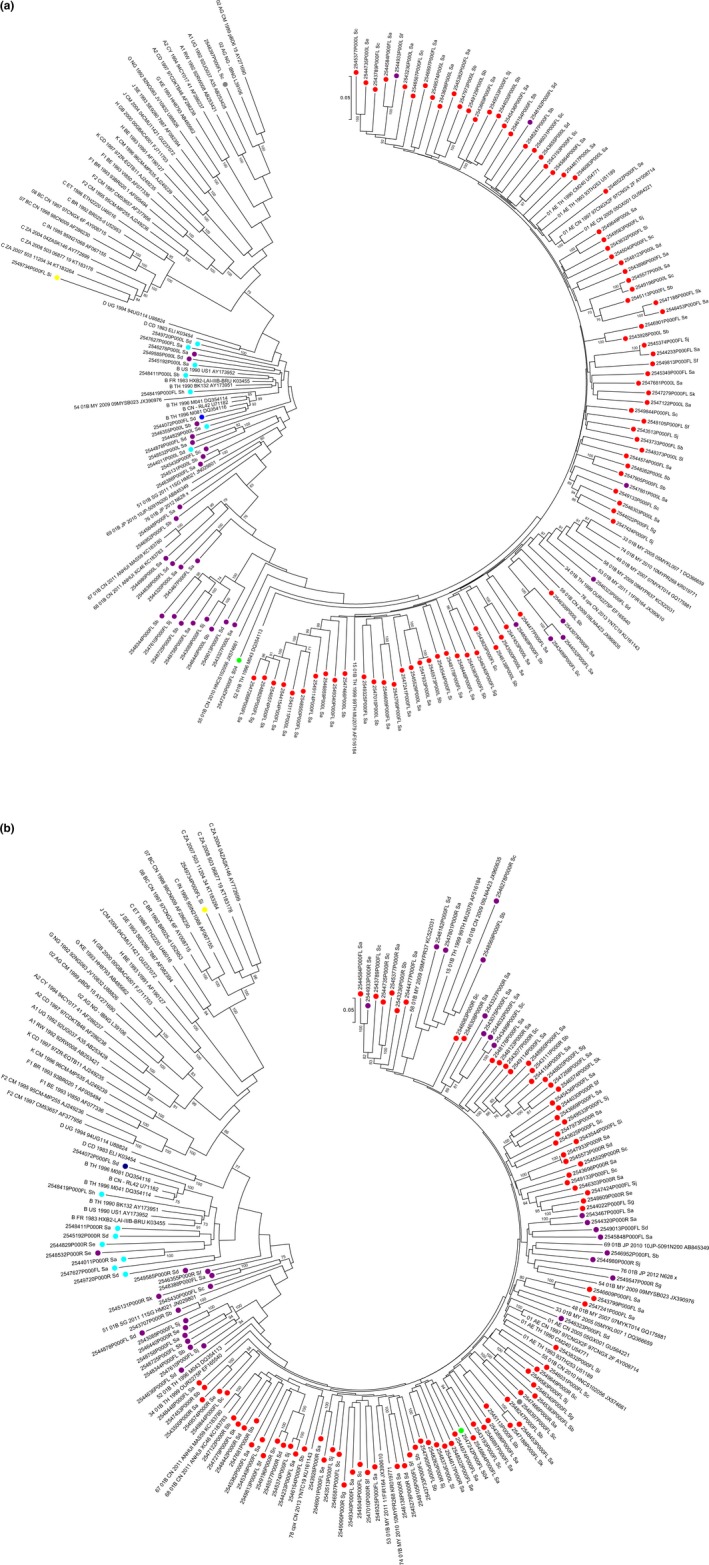
**Phylogenetic trees of sequences obtained from RV254 participants, and subtype and CRF reference sequences** **(a)** 5′‐half genome (HXB2 796‐5850), **(b)** 3′‐half genome (HXB2 4559‐9496). Coloured circles depict RV254 subtyping by phylogenetic analysis: subtype CRF01_AE (red), CRF01_AE/B recombinant (purple), B (light blue), B′ (dark blue), C (yellow), CRF01_AE/B/C recombinant (green) and CRF01_AE/CRF02_AG recombinant (grey).

**Figure 2:**
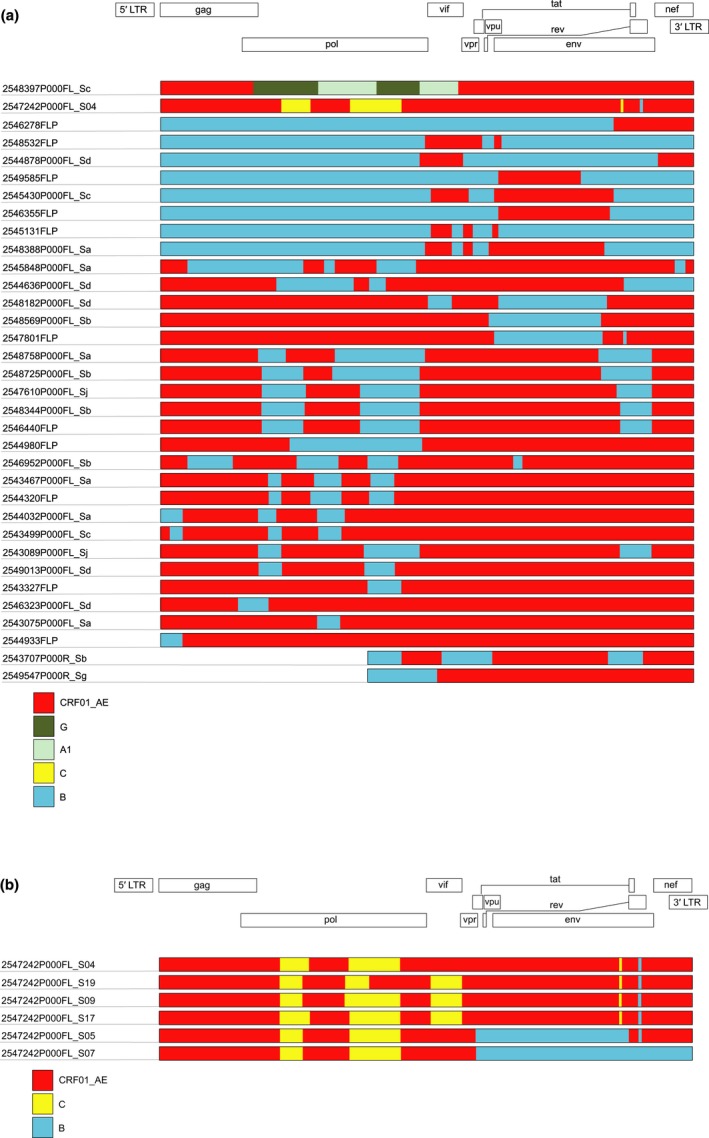
**Recombinant breakpoint structures for recombinant sequences (full‐length and patched half genomes) obtained from RV254 participants** **(a)** Apart from one CRF01_AE/CRF02_AG recombinant (2548397P000FL_Sc) and one CRF01_AE/B/C recombinant (2547242P000FL_S04), all other sequences were CRF01_AE/B recombinants. Only 3′‐half genomes were available for two participants (2543707P000R_Sb and 2549547P000R_Sg for 2543707 and 2549547 respectively). **(b)** Six different CRF01_AE/B/C recombinant structures were obtained from viruses harboured by RV254 participant 2547242.

Phylogenetic trees with reference sequences and RV254 half genomes are shown in Figure 1. Recombinant breakpoint structures of full genomes, patched corresponding half genomes and two 3′‐half genomes are shown in Figure 2a. The observed recombinant structures in RV254 participants were unique, and although some sequences shared similar breakpoints, none were consistent with published CRFs (up to CRF90_BF1, LANL database 05DEC2017 update). Six different recombinant structures containing subtype B, subtype C and CRF01_AE elements were seen in sequences from participant 2547242 (Figure 2b). When queried in HIV BLAST, the subtype C strain from participant 2549734 was most similar to subtype C South African strains; the 3.3‐kb CRF02_AG portion of the CRF01_AE/CRF02_AG recombinant found in participant 2548397 clustered with strains from Sweden, Cyprus, Ghana and Cameroon.

By phylogenetic analysis, only one of the eight subtype B infections (2544072) was a Thai B′ variant, although it contained the GPGR motif in V3 that is more commonly associated with Western B strains (Figure S1). The deduced V3 motifs and their frequency in subtype B strains of the LANL database are shown in Table S4.

### 3.2 RV254 and Asia transmission network

To determine whether RV254 sequences were closely related to other sequences within the cohort and in Asia, phylogenetic analysis was performed on *env* gp160 sequences of all subtypes from RV254 (n = 135) and Asia (n = 1794). Upon visual inspection of the alignment, two Asia sequences containing either an unusual 5′ insertion or deletion were identified and excluded from analysis (GenBank accession numbers EF036536 and KJ140245 respectively). The alignment used for the present analysis is contained in File S1.

**Figure 3:**
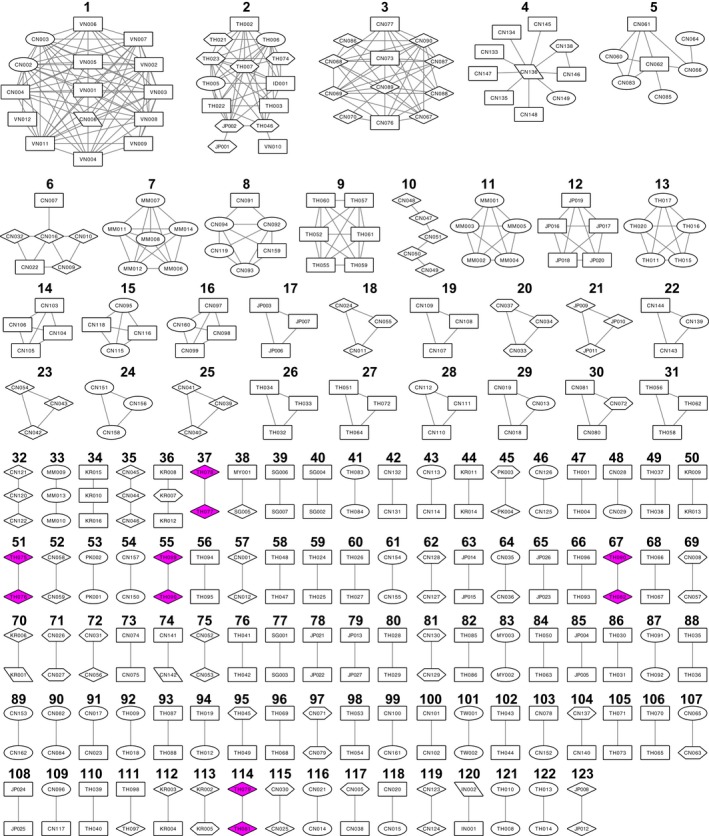
**Network diagram of sequences from RV254 participants in the larger HIV‐1 transmission network of Asia, arranged by cluster size (number of nodes, top to bottom)** Shapes (risk factors): ellipse, people who inject drugs (PWID); diamond, men who have sex with men (MSM) and homosexual (SG); hexagon, heterosexual (SH); parallelogram, mother to child (MB), nosocomial (NO), blood transfusion (PB) and haemophiliac (PH); rectangle, unknown (N/A). Magenta shading depicts RV254 participants. Edge lengths are not proportional to genetic distance between nodes.

A network diagram with identified potential transmission clusters is shown in Figure 3, and characteristics of the clusters are detailed in Table S5. Within RV254, five clusters of two nodes (also known as individuals or actors) each were identified: 2545573–2547933 (0% distance), 2544233–2545374 (2.07% distance), 2545577–2549196 (2.25% distance), 2543499–2544032 (2.6% distance) and 2547188–2548453 (2.7% distance). All were supported by bootstrap values of 100. None of the RV254 sequences clustered with sequences from Asia.

**Figure 4:**
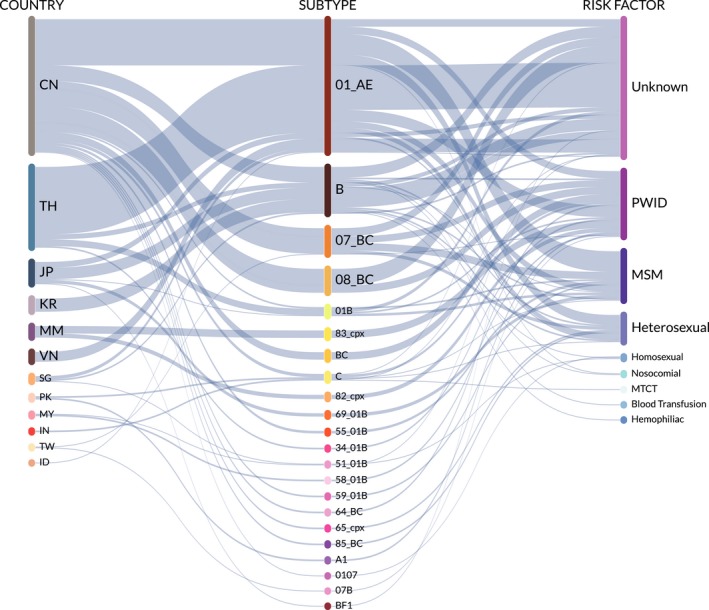
**Sankey diagram depicting the relative proportions of countries, subtypes and risk factors of nodes identified in the Asia transmission network**

Overall, 123 clusters were identified containing a total of 348 nodes and 444 edges as shown in Table S5. The nodes included sequences from China (162, 46.6%), Thailand (98, 28.2%), Japan (27, 7.8%), South Korea (16, 4.6%), Myanmar (14, 4.0%), Vietnam (12, 3.4%), Singapore (7, 2.0%), Pakistan (4, 1.1%), Malaysia (3, 0.9%), India (2, 0.6%), Taiwan (2, 0.6%) and Indonesia (1, 0.3%). Within our identified network, characteristics of the nodes (sampling country, subtype and risk factor) and their relative proportions are summarized in a Sankey diagram shown in Figure 4 and a complete description is shown in Table S6. Edges (also known as interactions or links) were predominantly from within the same country (389, 87.6%), with most involving two Chinese nodes (180, 40.5%). Edges involving at least one MSM or heterosexual were similar in proportion (88, 19.8% or 62, 14.0% respectively).

The median cluster size was a dyad, and most clusters were dyads (87, 70.7%) or triads (20, 16.3%); however, larger clusters were observed: 4‐node (3, 2.4%), 5‐ and 6‐node (each 4, 3.3%), and 7‐, 10‐, 12‐, 14‐ and 15‐node (each 1, 0.8%).

The largest cluster (15 nodes) was comprised of CRF01_AE sequences from Vietnam (11) and China (4) sampled between 1997 and 1999, and included PWID (2), nosocomial (1) and unknown (12) risk factors. Another international cluster included one 14‐node cluster with sequences from Thailand (10), Japan (2), Indonesia (1) and Vietnam (1). One dyad included sequences from Malaysia and Singapore. Each of the other clusters was limited to nodes from the same country.

For a large proportion of clusters, risk factor information was unknown for at least one node (74, 60.2%) or all nodes (44, 35.8%). Risk factors for at least one node in a cluster included PWID (39, 31.7%), MSM (25, 20.3%), heterosexuals (20, 16.3%), nosocomial exposures or homosexuals (each 2, 1.6%), and haemophiliac, blood transfusion or mother to child transmission (each 1, 0.8%). Six edges were pairs of one MSM and one heterosexual (four CRF01_AE CN‐CN, one subtype B KR‐KR and one subtype C PK‐PK), and one edge was a homosexual and haemophiliac pair (subtype B KR‐KR).

A vast majority of clusters were entirely Chinese (53, 43.1%) or entirely Thai (39, 31.7%). Ten (8.1%) clusters were Japanese, seven (5.7%) were South Korean, three (2.4%) each was either Myanmar or Singaporean, two (1.6%) were Pakistani and one (0.8%) cluster each were Indian, Malaysian or Taiwanese.

Of the 54 clusters with individuals from China, 25 included at least one PWID node, 15 included at least one heterosexual node and 14 included at least one MSM node. Of the forty‐one Thai clusters, eight included at least one PWID node, six included at least one MSM node and two included at least one heterosexual node.

## 4 Discussion

### 4.1 HIV in Thailand: A long and mature epidemic with increasing genetic diversity and expansion of recombinant viruses

Strains of CRF01_AE and subtype B, and their recombinants still represent the majority of circulating subtypes of HIV‐1 in Thailand. The subtype distribution of strains harboured by this cohort are similar to that of the Bangkok MSM Cohort Study, another primarily MSM and TGW cohort conducted between 2006 and 2014 37. Within the subtype B strains in the present study, we found a comparatively smaller proportion of the Thai variant (B′), which is consistent with a trend of diminishing B′ versus Western B as previously reported for another Thai cohort 38.

In Thailand, the major proportion of subtypes is represented by a predominance of CRF01_AE and a minority of subtype B strains, facilitating the sustained emergence of recombinants between the two. By way of comparison, in the Nigerian epidemic in which two major subtypes (G and CRF02_AG) co‐circulate, the relative proportion of recombinants is similar to that of the parental subtypes 39. A high proportion of multi‐subtype recombinants is also seen in the Tanzanian epidemic, in which subtypes A1, C and D co‐circulate 40. The Thai epidemic has seen the blending of once segregated risk groups, which provides opportunity for multiple exposures in individuals and the conditions for multiple subtypes to recombine. A recombinant virus can continue to recombine with other viruses, as is evident in the unique recombinant structures found in the RV254 cohort. That Thailand remains an international hub for MSM, business and tourism facilitates the introduction of non‐endemic subtypes (including South African variants of subtype C and West African variants of CRF02_AG seen in RV254) into the local population either as pure subtype strains or as recombinants 16, 17, 41, 42, 43.

Despite the theoretical challenges, immune correlates analyses conducted on the RV144 vaccine trial have found that vaccine‐induced antibodies targeting *env* V2 may confer protection against multiple circulating strains 13, 44. From a traditional vaccine standpoint, the induction of a broad response directed against even more diverse viruses could become more difficult, as would matching pure subtype immunogens to mixed subtype recombinant virus targets. Taken together, both increased HIV‐1 genetic diversity and the presence of various recombinant genome structures may further complicate vaccine design and highlights the need for a vaccine capable of inducing a broad immune response.

### 4.2 An HIV‐1 transmission network based on *env*

The envelope glycoprotein (encoded by *env*) mediates viral entry into host cells, and though it is partially shielded by glycans from the immune response, it possesses the highest genetic diversity and evolutionary rate of any gene in the HIV‐1 genome 45, 46, 47. Understanding *env*‐based networks may yield insight into viral pathogenesis, for a strain that has a high degree of connectivity may possess some advantage with respect to infectivity or fitness 48, 49, 50. Translated to approaches for vaccine design in which the HIV‐1 envelope glycoprotein is a target, trends in *env*‐based transmission networks could provide more contemporaneous data to show that current vaccine candidates may still cover the majority of circulating subtypes despite increasing diversity and recombination, and could also inform selection for vaccines in the pipeline.

Parameters that define a network also present caveats and limitations that must be considered in subsequent data analyses. For example, the genetic similarity threshold of the network is a parameter that can be varied depending on the research goal, set higher to identify long‐standing dynamics or lower to determine more recent and active transmission clusters 51. Plotting potential transmission networks can also reveal important information regarding the dynamics of an epidemic, allowing visualization of how previously segregated risk groups currently mix.

Here, the connection of RV254 sequences with the rest of Asia was investigated by a transmission network analysis that clustered similar *env* sequences with a pairwise distance of 3% or less. In known transmission pairs, divergence in the *env* gp160 gene has been reported to be less than 3% or approximately 75 nucleotides in the 2.5‐kb gene 52, 53, 54. At this relatively strict threshold, we identified five pairs of potential transmission linkages within RV254, and a total of 123 clusters within the larger Asia region. Four of the five RV254 pairs harboured CRF01_AE strains, and the other pair shared CRF01_AE/B recombinant strains. Interestingly, two of the CRF01_AE pairs share the same branch in a phylogenetic tree with RV254 *env* sequences, illustrating the dynamic intermixing within the cohort (Figure S2).

Nearly half of the clusters identified in this study included individuals from China belonging to different behavioural risk groups, indicating that the growing epidemic in China is also bridging risk groups. In recent years, HIV prevalence in MSM in China has increased dramatically, from 1.77% in 2000 to 5.98% in 2010 55. The local epidemic in some regions is much larger; prevalence among MSM in Chongqing who had visited saunas/bathhouses was 26.5% in 2007 56. Due to societal pressure and stigma, some MSM in China are married to a female partner, and are thereby likely to serve as bridges between risk groups 57.

Our network analysis does contain some inherent limitations. Although annotation of sequences with risk factor information became more widely adopted in the past decade, nearly half (869/1796, 48.4%) of the sequences we retrieved from the LANL HIV Database for the present analysis do not contain risk data. Risk reporting may be limited (by the individual or the researcher), due to stigma or criminalization of that risk, or to protect privacy in ongoing studies, among other reasons. Within a cluster though, risk for nodes with incomplete annotation may, to some extent, be deduced from association with nodes with known risk.

The method of identifying potential transmission networks is dependent on pairwise sequence comparisons, and therefore, the quality and quantity of input sequences supplies the foundation and boundaries of the proposed network. Selecting sequences of differential lengths and locations in the viral genome will undoubtedly affect the shape of the network. Many previous studies, including one with RV254 participants, have used partial *pol* gene sequences (approximately 1 kb) which are readily available from routine drug resistance screening but may possess limited resolution for transmission network analysis 51, 58. Although differences in methodologies experienced in the clinic or in the laboratory cannot be reconciled at the sequence level, with sufficient sequence annotation and appropriate clustering thresholds, interpretations of identified networks can still provide relevant data regarding epidemiologically active transmission clusters. Furthermore, transmission networks based on similar sequences can complement (or rule out) those established by contact tracing, which may be less reliable for more sexually active populations 59.

The majority of individuals living with HIV, including those who may transit between countries, are never enrolled in a study cohort, and thus, the comprehensive depth of any analysis of a given transmission network may be limited. However, the identified transmission network remains a reflection of the at‐risk population that could be accessed for other studies in prevention, treatment, cure or interventions such as pre‐exposure prophylaxis. Timely application of pre‐exposure prophylaxis may be key in controlling the HIV‐1 epidemic among MSM, and indeed is expanding rapidly in Thailand 16, 60. Furthermore, the bridging of risk groups and the branching of networks into the partners of high‐risk individuals suggests that multifaceted HIV‐1 prevention approaches should be developed to access these more complex networks.

## 5 Conclusions

The HIV‐1 epidemic in Asia is increasingly more complex at the molecular level and among transmission networks. The persistence of circulating viral subtypes coupled with repeatedly exposed and/or migrating hosts combine to promote the emergence of more diverse and unique recombinant forms of HIV‐1. Lessons learned from the relatively more mature Thai epidemic may be useful to consider while addressing and monitoring the expanding epidemics in other areas of Asia.

## Competing interests

The authors declare no competing interests.

## Authors’ contributions

EK, DJC, NC, MDS, JLE, PPhanuphak, NP, RJO, JHK, MLR, NLM and JA conceived, designed, implemented and managed the RV254/SEARCH 010 study protocol. DC and ST conceived and designed this analysis. DC, ESB, MB, AMO, RS, WR and ST were responsible for data collection. PPham and SP provided bioinformatics support. DC, ESB, PPham, EB, GK and ST contributed to data analysis and interpretation. DC conducted the literature search, wrote the manuscript and generated figures. All authors critically reviewed and approved the final version of the manuscript.

## Supporting information

**Figure S1**. Phylogenetic tree of subtype B strains from RV254 participants with reference Western subtype B (right tree branches) and Thai B′ strains (left tree branch). Pure subtype B strains from RV254 participants were primarily Western B (light blue circles, n = 7) versus Thai B′ (dark blue circles, n = 1). Subtype J (open triangles) is plotted as an outsider group.
**Figure S2**. Phylogenetic tree of *env* gp160 gene sequences from RV254 participants. Coloured circles depict RV254 subtyping by phylogenetic analysis: subtype CRF01_AE (red), CRF01_AE/B recombinant (purple), B (light blue), B′ (dark blue), C (yellow), CRF01_AE/B/C recombinant (green) and CRF01_AE/CRF02_AG recombinant (grey).
**Table S1**. Summary and details of RV254 and subtype reference sequences included in the present analysis. a) Summary of sequences from RV254 participants obtained by single‐genome sequencing, b) details of sequences listed by participant ID; FL = full genome, LH = 5′‐half genome, RH = 3′‐half genome, LH + RH = 5′‐ and 3′‐half genomes, ENV = envelope gene only, c) details of subtype reference sequences (full genome), d) details of subtype B, B′, and J reference sequences (*env* gp160).
**Table S2**. Details of Asia *env* gp160 sequences downloaded from the Los Alamos National Laboratory (LANL) HIV Database and included in the present analysis.
**Table S3**. Socio‐demographic characteristics of RV254 study participants.
**Table S4**. RV254 subtype B V3 loop tip motifs and their global frequencies.
**Table S5**. Clusters identified in Asian HIV‐1 transmission network.
**Table S6**. Description of nodes identified in Asian HIV‐1 Transmission Network. a) Summary of nodes, b) detailed list of nodes. Country codes: China (CN), India (IN), Indonesia (ID), Japan (JP), Malaysia (MY), Myanmar (MM), Pakistan (PK), Singapore (SG), South Korea (KR), Taiwan (TW), Thailand (TH), Vietnam (VN). Risk factors: blood transfusion (PB), haemophiliac (PH), heterosexual (SH), homosexual (SG), people who inject drugs (PWID), men who have sex with men (MSM), mother to child (MB), nosocomial (NO), unknown (N/A).
**File S1**. Alignment of RV254 and Asia *env* gp160 sequences used in the present analysis.
