# Supplementary material for: Molecular epidemiology of a primarily MSM acute HIV‐1 cohort in Bangkok, Thailand and connections within networks of transmission in Asia
Source: J Int AIDS Soc. 2018 Nov 22;21(11):e25204. doi: 10.1002/jia2.25204 (PMC6282942; doi:10.1002/jia2.25204)
Supplement: Supplementary file 2 — Figure S1. Phylogenetic tree of subtype B strains from RV254 participants with reference Western subtype B (right tree branches) and Thai B′ strains (left tree branch). Pure subtype B strains from RV254 participants were primarily Western B (light blue circles, n = 7) versus Thai B′ (dark blue circles, n = 1). Subtype J (open triangles) is plotted as an outsider group. Figure S2. Phylogenetic tree of env gp160 gene sequences from RV254 participants. Coloured circles depict RV254 subtyping by phylogenetic analysis: subtype CRF01_AE (red), CRF01_AE/B recombinant (purple), B (light blue), B′ (dark blue), C (yellow), CRF01_AE/B/C recombinant (green) and CRF01_AE/CRF02_AG recombinant (grey). Table S1. Summary and details of RV254 and subtype reference sequences included in the present analysis. a) Summary of sequences from RV254 participants obtained by single‐genome sequencing, b) details of sequences listed by participant ID; FL = full genome, LH = 5′‐half genome, RH = 3′‐half genome, LH + RH = 5′‐ and 3′‐half genomes, ENV = envelope gene only, c) details of subtype reference sequences (full genome), d) details of subtype B, B′, and J reference sequences (env gp160). Table S2. Details of Asia env gp160 sequences downloaded from the Los Alamos National Laboratory (LANL) HIV Database and included in the present analysis. Table S3. Socio‐demographic characteristics of RV254 study participants. Table S4. RV254 subtype B V3 loop tip motifs and their global frequencies. Table S5. Clusters identified in Asian HIV‐1 transmission network. Table S6. Description of nodes identified in Asian HIV‐1 Transmission Network. a) Summary of nodes, b) detailed list of nodes. Country codes: China (CN), India (IN), Indonesia (ID), Japan (JP), Malaysia (MY), Myanmar (MM), Pakistan (PK), Singapore (SG), South Korea (KR), Taiwan (TW), Thailand (TH), Vietnam (VN). Risk factors: blood transfusion (PB), haemophiliac (PH), heterosexual (SH), homosexual (SG), people who inject drugs (PWID), men who [file JIA2-21-e25204-s002.pdf]

Supporting Information

Molecular epidemiology of a primarily MSM acute HIV-1 cohort in Bangkok, Thailand and connections within networks of transmission in Asia

David Chang[1,2], Eric Sanders-Buell[1,2], Meera Bose[1,2], Anne Marie O'Sullivan[1,2], Phuc Pham[1,2], Eugene Kroon[3], Donn J. Colby[3], Rujipas Sirijatuphat[1,4], Erik Billings[1,2], Suteeraporn Pinyakorn[1,2], Nitiya Chomchey[3], Wiriya Rutvisuttinunt[5,6], Gustavo Kijak[1,2,#], Mark de Souza[2,3], Jean-Louis Excler[1,2], Praphan Phanuphak[7], Nittaya Phanuphak[7], Robert J. O'Connell[1,5], Jerome H. Kim[1,8], Merlin L. Robb[1,2], Nelson L. Michael[1], Jintanat Ananworanich[1,2,3,9], and Sodsai Tovanabutra[1,2,§], on behalf of the RV254/SEARCH 010 Study Group

[1] United States Military HIV Research Program, Walter Reed Army Institute of Research, Silver Spring, MD, USA
[2] The Henry M. Jackson Foundation for the Advancement of Military Medicine, Bethesda, MD, USA
[3] SEARCH, Bangkok, Thailand
[4] Department of Medicine, Faculty of Medicine Siriraj Hospital, Mahidol University, Bangkok, Thailand
[5] Department of Retrovirology, Armed Forces Research Institute of Medical Sciences, Bangkok, Thailand
[6] Viral Diseases Branch, Walter Reed Army Institute of Research, Silver Spring, MD, USA
[7] The Thai Red Cross AIDS Research Centre, Bangkok, Thailand
[8] International Vaccine Institute, Seoul, South Korea
[9] Department of Global Health, Academic Medical Center, University of Amsterdam, Amsterdam, The Netherlands
[#] Present affiliation: GSK Vaccines, Rockville, MD, USA

[§] Corresponding Author: Sodsai Tovanabutra
503 Robert Grant Avenue, Room 2N25
Silver Spring, MD 20910
USA
Phone: +1 301 319 9993
Email: stovanabutra@hivresearch.org

Figure Legends for Supporting Information:

Figure S1. Phylogenetic tree of subtype B strains from RV254 participants with reference Western subtype B (right tree branches) and Thai B′ strains (left tree branch). Pure subtype B strains from RV254 participants were primarily Western B (light blue circles, n=7), versus Thai B' (dark blue circles, n=1). Subtype J (open triangles) is plotted as an outsider group.

Figure S2. Phylogenetic tree of *env* gp160 gene sequences from RV254 participants. Colored circles depict RV254 subtyping by phylogenetic analysis: subtype CRF01_AE (red), CRF01_AE/B recombinant (purple), B (light blue), B′ (dark blue), C (yellow), CRF01_AE/B/C recombinant (green), and CRF01_AE/CRF02_AG recombinant (grey).

Table S1. Summary and details of RV254 and subtype reference sequences included in the present analysis. a) Summary of sequences from RV254 participants obtained by single genome sequencing, b) Details of sequences listed by participant ID; FL = full genome, LH = 5′- half genome, RH = 3′ half genome, LH+RH = 5′- and 3′- half genomes, ENV = envelope gene only, c) Details of subtype reference sequences (full genome), d) Details of subtype B, B′, and J reference sequences (*env* gp160).

Table S2. Details of Asia *env* gp160 sequences downloaded from the Los Alamos National Laboratory (LANL) HIV Database and included in the present analysis.

Table S3. Socio-demographic characteristics of RV254 study participants.

Table S4. RV254 subtype B V3 loop tip motifs and their global frequencies.

Table S5. Clusters identified in Asian HIV-1 transmission network.

Table S6. Description of nodes identified in Asian HIV-1 Transmission Network. a) Summary of nodes, b) Detailed list of nodes. Country codes: China (CN), India (IN), Indonesia (ID), Japan (JP), Malaysia (MY), Myanmar (MM), Pakistan (PK), Singapore (SG), South Korea (KR),

Taiwan (TW), Thailand (TH), Vietnam (VN). Risk factors: Blood transfusion (PB), hemophiliac (PH), heterosexual (SH), homosexual (SG), people who inject drugs (PWID), men who have sex with men (MSM), mother to child (MB), nosocomial (NO), unknown (N/A).

File S1. Alignment of RV254 and Asia *env* gp160 sequences used in the present analysis.

Figure S1

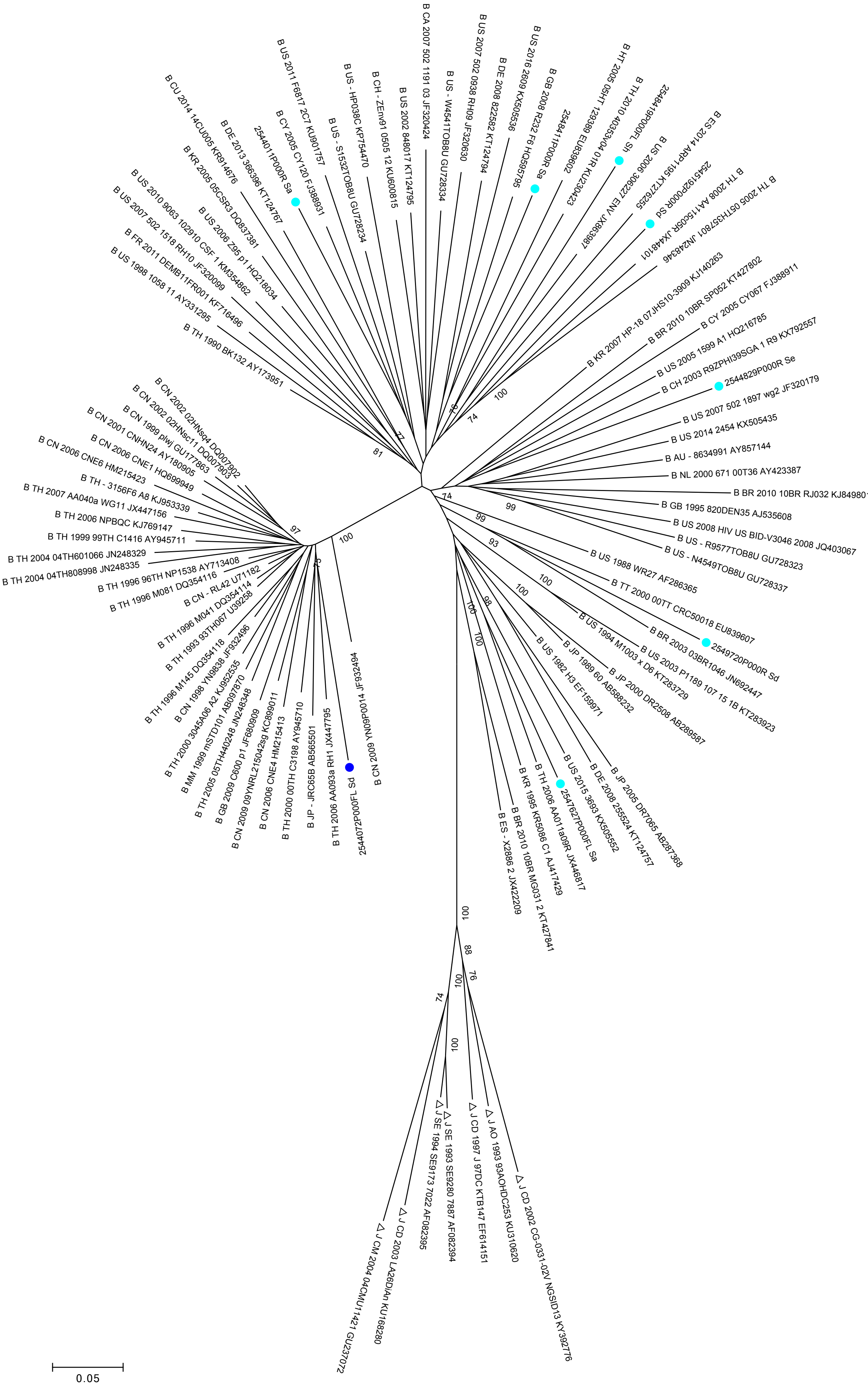
B CA 2007 502 1191 03 JF320424
B US 2007 502 0938 RH09 JF320630
B US 2016 2609 KX505536
B HT 2005 05HT 129389 EU839602
B TH 2010 4033V04 01R KU230423
2548419P00FL Sh
B ES 2014 ARP1195 KT276255
B US 2006 306227 ENV JX863987
2545192P00R Sd
B TH 2008 AA1150SR JX448101
B TH 2005 05TH357801 JN248346
B US - W4541TOB8U GU728334
B DE 2008 825582 KT124794
B GB 2008 R232 F6 HQ595795
2548411P00R Sa
B US 2002 848017 KT124795
B CH - ZEnv91 0505 12 KU600815
B US - HP038C KP754470
B US - S1532TOB8U GU728234
B US 2011 F6817 2C7 KU901757
B CY 2005 CY120 FJ388931
2544011P000R Sa
B DE 2013 366396 KT124767
B CU 2014 14CU005 KR914676
B KR 2005 05CSR3 DQ837381
B US 2006 Z95 p1 HQ218034
B US 2010 9063 102910 CSF 1 KM354862
B US 2007 502 1518 RH10 JF320099
B FR 2011 DEMB11FR001 KF716496
B US 1998 1058 11 AY331295
B TH 1990 BK132 AY173951
B KR 2007 HP-18 07JHS10-3909 KJ140263
B BR 2010 10BR SP052 KT427802
B CY 2005 CY067 FJ388911
B US 2005 1599 A1 HQ216785
B CH 2003 R9ZPHI39SGA 1 R9 KX792557
2544829P000R Se
B US 2007 502 1897 wg2 JF320179
B US 2014 2454 KX505435
B AU - 8634991 AY857144
B NL 2000 671 00T36 AY423387
B BR 2010 10BR RJ032 KJ849801
B GB 1995 820DEN35 AJ535608
B US 2008 HIV US BID-V3046 2008 JQ403067
B US - R9577TOB8U GU728323
B US - N4549TOB8U GU728337
B US 1988 WR27 AF286365
B TT 2000 00TT CRC50018 EU839607
B BR 2003 03BR1046 JN692447
2549720P000R Sd
B US 1994 M1003 x D6 KT283729
B US 2003 P1189 107 15 1B KT283923
B JP 2000 DR2508 AB289587
B JP 1989 60 AB588232
B US 1982 H3 EF159971
B JP 2005 DR7065 AB287368
B DE 2008 255524 KT124757
B US 2015 3693 KX505552
2547627P000FL Sa
B TH 2006 AA011a09R JX446817
B KR 1995 KR5086 C1 AJ417429
B BR 2010 10BR MG031 2 KT427841
B ES - X2886 2 JX422209
B CN 2002 02HNsq4 DQ007902
B CN 2002 02HNsc11 DQ007903
B CN 1999 plwj GU177863
B CN 2001 CNHN24 AY180905
B CN 2006 CNE1 HQ699949
B CN 2006 CNE6 HM215423
B TH - 3156F6 A8 KJ953339
B TH 2007 AA040a WG11 JX447156
B TH 2006 NPBQC KJ769147
B TH 1999 99TH C1416 AY945711
B TH 2004 04TH601066 JN248329
B TH 2004 04TH808998 JN248335
B TH 1996 96TH NP1538 AY713408
B TH 1996 M081 DQ354116
B CN - RL42 U71182
B TH 1996 M041 DQ354114
B TH 1993 93TH067 U39258
B TH 1996 M145 DQ354118
B CN 1998 YN9838 JF932496
B TH 2000 3045A06 A2 KJ952535
B MM 1999 mSTD101 AB097870
B TH 2005 05TH440248 JN248348
B GB 2009 C600 p1 JF680909
B CN 2009 09YNRL215042sg KC899011
B CN 2006 CNE4 HM215413
B TH 2000 00TH C3198 AY945710
B JP - JRC65B AB565501
B TH 2006 AA093a RH1 JX447795
2544072P000FL Sd
B CN 2009 YN09P0014 JF932494
△ J CD 2002 CG-0331-02V NGSID13 KY392776
△ J AO 1993 93AOHDC253 KU310620
△ J CD 1997 J 97DC KTB147 EF614151
△ J SE 1993 SE9280 7887 AF082394
△ J SE 1994 SE9173 7022 AF082395
△ J CD 2003 LA26DiAn KU168280
△ J CM 2004 04CMU11421 GU237072
100
74
77
81
97
100
99
99
93
100
100
98
100
100
100
88
76
100
74
100
75
0.05

Figure S2

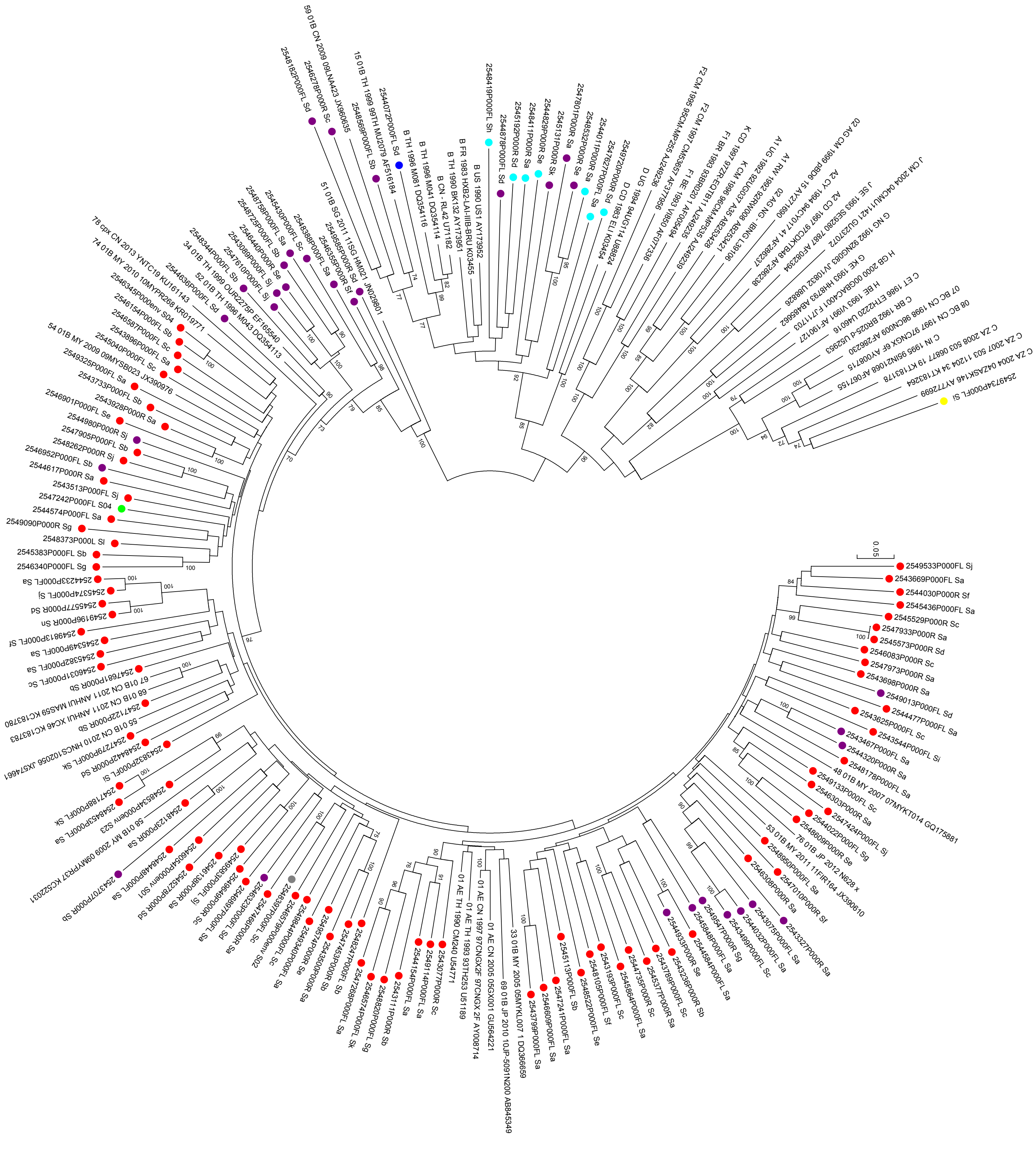

Table S1. Summary and details of RV254 and subtype reference sequences included in the present analysis

a) Summary of sequences from RV254 participants obtained by single genome sequencing

**Genomic Region**	Number of Individuals (n=138)
Full genome (FL)	80
Corresponding half genomes (both 5′- and 3′- half genomes) (LH+RH)	45
5′- single half genome only (LH)	3
3′- single half genome only (RH)	6
Envelope gene only (ENV)	4
**Sampling Year**	
2009	10
2010	22
2011	25
2012	8
2013	17
2014	29
2015	27
**Subtype by Sequencing**	
CRF01_AE	88
CRF01_AE/B Recombinant	30
B	8
CRF01_AE (RH)	4
CRF01_AE (LH)	3
CRF01_AE/B Recombinant (RH)	2
CRF01_AE/CRF02_AG recombinant	1
CRF01_AE/B/C Recombinant	1
C	1

b) Details of RV254 sequences listed by participant ID

**Participant ID**	**Sequence Name**	**Subtype by Sequencing**	**Genome Region**	**Sampling Year**	**GenBank Accession**
2543928	2543928P000L_Sb	CRF01_AE	LH	2009	MG989627
2543928	2543928P000R_Sa	CRF01_AE	RH	2009	MG989577
2544933	2544933P000L_Sf	CRF01_AE/B Recombinant	LH	2009	MG989634
2544933	2544933P000R_Se	CRF01_AE/B Recombinant	RH	2009	MG989584
2545131	2545131P000L_Sb	CRF01_AE/B Recombinant	LH	2009	MG989636
2545131	2545131P000R_Sk	CRF01_AE/B Recombinant	RH	2009	MG989586
2545573	2545573P000L_Sb	CRF01_AE	LH	2009	MG989640
2545573	2545573P000R_Sd	CRF01_AE	RH	2009	MG989591
2545577	2545577P000L_Sa	CRF01_AE	LH	2009	MG989641
2545577	2545577P000R_Sd	CRF01_AE	RH	2009	MG989592
2546083	2546083P000L_Sa	CRF01_AE	LH	2009	MG989642
2546083	2546083P000R_Sc	CRF01_AE	RH	2009	MG989593
2547933	2547933P000L_Sa	CRF01_AE	LH	2009	MG989656
2547933	2547933P000R_Sa	CRF01_AE	RH	2009	MG989606
2548262	2548262P000L_Sb	CRF01_AE	LH	2009	MG989659
2548262	2548262P000R_Sj	CRF01_AE	RH	2009	MG989609
2548442	2548442P000R_Sd	CRF01_AE	RH	2009	MG989611
2549720	2549720P000L_Sd	B	LH	2009	MG989667
2549720	2549720P000R_Sd	B	RH	2009	MG989620
2543625	2543625P000FL_Sc	CRF01_AE	FL	2010	MG989497
2543698	2543698P000L_Sa	CRF01_AE	LH	2010	MG989626
2543698	2543698P000R_Sa	CRF01_AE	RH	2010	MG989575
2543733	2543733P000FL_Sb	CRF01_AE	FL	2010	MG989499
2543789	2543789P000FL_Sc	CRF01_AE	FL	2010	MG989500
2543832	2543832P000FL_Si	CRF01_AE	FL	2010	MG989502
2544011	2544011P000L_Sd	B	LH	2010	MG989628
2544011	2544011P000R_Sa	B	RH	2010	MG989578
2544617	2544617P000L_Sa	CRF01_AE	LH	2010	MG989631
2544617	2544617P000R_Sa	CRF01_AE	RH	2010	MG989581
2545113	2545113P000FL_Sb	CRF01_AE	FL	2010	MG989515
2545192	2545192P000L_Sa	B	LH	2010	MG989637
2545192	2545192P000R_Sd	B	RH	2010	MG989587
2545374	2545374P000FL_Sj	CRF01_AE	FL	2010	MG989517

2545436	2545436P000FL_Sa	CRF01_AE	FL	2010	MG989521
2546303	2546303P000L_Sa	CRF01_AE	LH	2010	MG989645
2546303	2546303P000R_Sa	CRF01_AE	RH	2010	MG989596
2546308	2546308P000L_Sb	CRF01_AE	LH	2010	MG989646
2546308	2546308P000R_Sa	CRF01_AE	RH	2010	MG989597
2546609	2546609P000FL_Sa	CRF01_AE	FL	2010	MG989530
2547122	2547122P000L_Sa	CRF01_AE	LH	2010	MG989651
2547122	2547122P000R_Sb	CRF01_AE	RH	2010	MG989601
2547973	2547973P000L_Sb	CRF01_AE	LH	2010	MG989657
2547973	2547973P000R_Sa	CRF01_AE	RH	2010	MG989607
2548388	2548388P000FL_Sa	CRF01_AE/B Recombinant	FL	2010	MG989549
2548950	2548950P000FL_Sa	CRF01_AE	FL	2010	MG989559
2549133	2549133P000FL_Sc	CRF01_AE	FL	2010	MG989562
2549196	2549196P000L_Sc	CRF01_AE	LH	2010	MG989663
2549196	2549196P000R_Sn	CRF01_AE	RH	2010	MG989615
2549583	2549583P000FL_Sj	CRF01_AE	FL	2010	MG989566
2549844	2549844P000FL_Sc	CRF01_AE	FL	2010	MG989569
2543077	2543077P000R_Sc	CRF01_AE (RH)	RH	2011	MG989570
2543467	2543467P000FL_Sa	CRF01_AE/B Recombinant	FL	2011	MG989493
2543513	2543513P000FL_Sj	CRF01_AE	FL	2011	MG989495
2544022	2544022P000FL_Sg	CRF01_AE	FL	2011	MG989504
2544030	2544030P000L_Sb	CRF01_AE	LH	2011	MG989629
2544030	2544030P000R_Sf	CRF01_AE	RH	2011	MG989579
2544574	2544574P000FL_Sa	CRF01_AE	FL	2011	MG989510
2544735	2544735P000L_Se	CRF01_AE	LH	2011	MG989632
2544735	2544735P000R_Sc	CRF01_AE	RH	2011	MG989582
2545349	2545349P000FL_Sa	CRF01_AE	FL	2011	MG989516
2545529	2545529P000L_Sa	CRF01_AE	LH	2011	MG989639
2545529	2545529P000R_Sc	CRF01_AE	RH	2011	MG989590
2546154	2546154P000FL_Sb	CRF01_AE	FL	2011	MG989525
2546340	2546340P000FL_Sg	CRF01_AE	FL	2011	MG989527
2546355	2546355P000L_Sb	CRF01_AE/B Recombinant	LH	2011	MG989647
2546355	2546355P000R_Sf	CRF01_AE/B Recombinant	RH	2011	MG989598
2547010	2547010P000L_Sb	CRF01_AE	LH	2011	MG989650
2547010	2547010P000R_Sf	CRF01_AE	RH	2011	MG989600
2547242	2547242P000FL_S04	CRF01_AE/B/C Recombinant	FL	2011	MG989536
2547453	2547453P000L_Sa	CRF01_AE	LH	2011	MG989652
2547453	2547453P000R_Sb	CRF01_AE	RH	2011	MG989602
2547681	2547681P000L_Sa	CRF01_AE	LH	2011	MG989654
2547681	2547681P000R_Sb	CRF01_AE	RH	2011	MG989604
2547905	2547905P000FL_Sb	CRF01_AE	FL	2011	MG989542
2548123	2548123P000L_Sd	CRF01_AE	LH	2011	MG989658
2548123	2548123P000R_Sa	CRF01_AE	RH	2011	MG989608
2548178	2548178P000FL_Sa	CRF01_AE	FL	2011	MG989544
2548397	2548397P000FL_Sc	CRF01_AE/CRF02_AG recombinant	FL	2011	MG989550
2548534	2548534P000env_S23	CRF01_AE	ENV	2011	MG989671
2549114	2549114P000FL_Sa	CRF01_AE	FL	2011	MG989561
2549129	2549129P000L_Sb	CRF01_AE (LH)	LH	2011	MG989662
2549533	2549533P000FL_Sj	CRF01_AE	FL	2011	MG989565
2549574	2549574P000L_Sa	CRF01_AE	LH	2011	MG989664
2549574	2549574P000R_Se	CRF01_AE	RH	2011	MG989617
2543193	2543193P000FL_Sc	CRF01_AE	FL	2012	MG989492
2543669	2543669P000FL_Sa	CRF01_AE	FL	2012	MG989498
2544477	2544477P000FL_Sa	CRF01_AE	FL	2012	MG989509
2545382	2545382P000FL_Sa	CRF01_AE	FL	2012	MG989518
2545864	2545864P000FL_Sa	CRF01_AE	FL	2012	MG989523
2546897	2546897P000FL_Sa	CRF01_AE	FL	2012	MG989531
2547466	2547466P000L_Sb	CRF01_AE	LH	2012	MG989653
2547466	2547466P000R_Sa	CRF01_AE	RH	2012	MG989603
2549649	2549649P000L_Sa	CRF01_AE	LH	2012	MG989666
2549649	2549649P000R_Sc	CRF01_AE	RH	2012	MG989619
2543089	2543089P000FL_Sj	CRF01_AE/B Recombinant	FL	2013	MG989491
2543499	2543499P000FL_Sc	CRF01_AE/B Recombinant	FL	2013	MG989494
2543799	2543799P000FL_Sa	CRF01_AE	FL	2013	MG989501
2544072	2544072P000FL_Sd	B	FL	2013	MG989506
2544233	2544233P000FL_Sa	CRF01_AE	FL	2013	MG989508

2544320	2544320P000L_Sa	CRF01_AE/B Recombinant	LH	2013	MG989630
2544320	2544320P000R_Sa	CRF01_AE/B Recombinant	RH	2013	MG989580
2544829	2544829P000L_Se	B	LH	2013	MG989633
2544829	2544829P000R_Se	B	RH	2013	MG989583
2544878	2544878P000FL_Sd	CRF01_AE/B Recombinant	FL	2013	MG989513
2546054	2546054P000env_S01	CRF01_AE	ENV	2013	MG989668
2546345	2546345P000env_S04	CRF01_AE	ENV	2013	MG989669
2546579	2546579P000env_S02	CRF01_AE	ENV	2013	MG989670
2546859	2546859P000L_Sa	CRF01_AE (LH)	LH	2013	MG989649
2546952	2546952P000FL_Sb	CRF01_AE/B Recombinant	FL	2013	MG989533
2548448	2548448P000FL_Sa	CRF01_AE	FL	2013	MG989552
2548569	2548569P000FL_Sb	CRF01_AE/B Recombinant	FL	2013	MG989555
2549325	2549325P000FL_Sa	CRF01_AE	FL	2013	MG989563
2549340	2549340P000FL_Sa	CRF01_AE	FL	2013	MG989564
2543075	2543075P000FL_Sa	CRF01_AE/B Recombinant	FL	2014	MG989490
2543236	2543236P000L_Sa	CRF01_AE	LH	2014	MG989622
2543236	2543236P000R_Sb	CRF01_AE	RH	2014	MG989572
2543500	2543500P000L_Sa	CRF01_AE	LH	2014	MG989624
2543500	2543500P000R_Sa	CRF01_AE	RH	2014	MG989574
2543655	2543655P000L_Sd	CRF01_AE (LH)	LH	2014	MG989625
2543707	2543707P000R_Sb	CRF01_AE/B Recombinant (RH)	RH	2014	MG989576
2543896	2543896P000FL_Sa	CRF01_AE	FL	2014	MG989503
2544584	2544584P000FL_Sa	CRF01_AE	FL	2014	MG989511
2544980	2544980P000L_Sa	CRF01_AE/B Recombinant	LH	2014	MG989635
2544980	2544980P000R_Sj	CRF01_AE/B Recombinant	RH	2014	MG989585
2545040	2545040P000FL_Sc	CRF01_AE	FL	2014	MG989514
2545383	2545383P000FL_Sb	CRF01_AE	FL	2014	MG989519
2545430	2545430P000FL_Sc	CRF01_AE/B Recombinant	FL	2014	MG989520
2545848	2545848P000FL_Sa	CRF01_AE/B Recombinant	FL	2014	MG989522
2546031	2546031P000FL_Sc	CRF01_AE	FL	2014	MG989524
2546138	2546138P000L_Sb	CRF01_AE	LH	2014	MG989643
2546138	2546138P000R_Sa	CRF01_AE	RH	2014	MG989594
2546278	2546278P000L_Sa	CRF01_AE/B Recombinant	LH	2014	MG989644
2546278	2546278P000R_Sc	CRF01_AE/B Recombinant	RH	2014	MG989595
2546323	2546323P000FL_Sd	CRF01_AE/B Recombinant	FL	2014	MG989526
2546587	2546587P000FL_Sc	CRF01_AE	FL	2014	MG989529
2546901	2546901P000FL_Se	CRF01_AE	FL	2014	MG989532
2547241	2547241P000FL_Sa	CRF01_AE	FL	2014	MG989535
2547279	2547279P000FL_Sk	CRF01_AE	FL	2014	MG989538
2547610	2547610P000FL_Sj	CRF01_AE/B Recombinant	FL	2014	MG989540
2547801	2547801P000L_Sa	CRF01_AE/B Recombinant	LH	2014	MG989655
2547801	2547801P000R_Sa	CRF01_AE/B Recombinant	RH	2014	MG989605
2548373	2548373P000L_Sl	CRF01_AE	LH	2014	MG989548
2548725	2548725P000FL_Sb	CRF01_AE/B Recombinant	FL	2014	MG989556
2548758	2548758P000FL_Sa	CRF01_AE/B Recombinant	FL	2014	MG989557
2549013	2549013P000FL_Sd	CRF01_AE/B Recombinant	FL	2014	MG989560
2549090	2549090P000R_Sg	CRF01_AE (RH)	RH	2014	MG989614
2549585	2549585P000L_Sd	CRF01_AE/B Recombinant	LH	2014	MG989665
2549585	2549585P000R_Sd	CRF01_AE/B Recombinant	RH	2014	MG989618
2549734	2549734P000FL_Si	C	FL	2014	MG989567
2543111	2543111P000L_Sa	CRF01_AE	LH	2015	MG989621
2543111	2543111P000R_Sb	CRF01_AE	RH	2015	MG989571
2543327	2543327P000L_Sa	CRF01_AE/B Recombinant	LH	2015	MG989623
2543327	2543327P000R_Sa	CRF01_AE/B Recombinant	RH	2015	MG989573
2543544	2543544P000FL_Si	CRF01_AE	FL	2015	MG989496
2544032	2544032P000FL_Sa	CRF01_AE/B Recombinant	FL	2015	MG989505
2544154	2544154P000FL_Sa	CRF01_AE	FL	2015	MG989507
2544636	2544636P000FL_Sd	CRF01_AE/B Recombinant	FL	2015	MG989512
2545278	2545278P000R_Sd	CRF01_AE (RH)	RH	2015	MG989588
2545377	2545377P000L_Sc	CRF01_AE	LH	2015	MG989638
2545377	2545377P000R_Sa	CRF01_AE	RH	2015	MG989589
2546440	2546440P000L_Sb	CRF01_AE/B Recombinant	LH	2015	MG989648
2546440	2546440P000R_Se	CRF01_AE/B Recombinant	RH	2015	MG989599
2546574	2546574P000FL_Sk	CRF01_AE	FL	2015	MG989528
2547188	2547188P000FL_Sk	CRF01_AE	FL	2015	MG989534
2547268	2547268P000FL_Sa	CRF01_AE	FL	2015	MG989537

2547424	2547424P000FL_Sj	CRF01_AE	FL	2015	MG989539
2547627	2547627P000FL_Sa	B	FL	2015	MG989541
2548105	2548105P000FL_Sf	CRF01_AE	FL	2015	MG989543
2548182	2548182P000FL_Sd	CRF01_AE/B Recombinant	FL	2015	MG989545
2548247	2548247P000FL_Sb	CRF01_AE	FL	2015	MG989546
2548344	2548344P000FL_Sb	CRF01_AE/B Recombinant	FL	2015	MG989547
2548411	2548411P000L_Sb	B	LH	2015	MG989660
2548411	2548411P000R_Sa	B	RH	2015	MG989610
2548419	2548419P000FL_Sh	B	FL	2015	MG989551
2548453	2548453P000FL_Sa	CRF01_AE	FL	2015	MG989553
2548522	2548522P000FL_Se	CRF01_AE	FL	2015	MG989554
2548532	2548532P000L_Sa	CRF01_AE/B Recombinant	LH	2015	MG989661
2548532	2548532P000R_Se	CRF01_AE/B Recombinant	RH	2015	MG989612
2548609	2548609P000R_Se	CRF01_AE (RH)	RH	2015	MG989613
2548820	2548820P000FL_Sg	CRF01_AE	FL	2015	MG989558
2549547	2549547P000R_Sg	CRF01_AE/B Recombinant (RH)	RH	2015	MG989616
2549813	2549813P000FL_Sf	CRF01_AE	FL	2015	MG989568

c) Details of subtype reference sequences (full genome)

**Subtype Reference**	**Sequence Name**	**Sampling Country**	**Sampling Year**	**GenBank Accession**
A1	92RW008	RW	1992	AB253421
A1	92UG037_A35	UG	1992	AB253428
A2	97CDKTB48	CD	1997	AF286238
A2	94CY017_41	CY	1994	AF286237
B	RL42	CN	-	U71182
B	HXB2-LAI-IIIB-BRU	FR	1983	K03455
B	BK132	TH	1990	AY173951
B	M041	TH	1996	DQ354114
B	M081	TH	1996	DQ354116
B	US1	US	1990	AY173952
C	BR025-d	BR	1992	U52953
C	ETH2220	ET	1986	U46016
C	95IN21068	IN	1995	AF067155
C	04ZASK146	ZA	2004	AY772699
C	503_11204_34	ZA	2007	KT183264
C	503_06877_19	ZA	2008	KT183178
D	ELI	CD	1983	K03454
D	94UG114	UG	1994	U88824
F1	VI850	BE	1993	AF077336
F1	93BR020_1	BR	1993	AF005494
F2	95CM-MP255	CM	1995	AJ249236
F2	CM53657	CM	1997	AF377956
G	HH8793	KE	1993	AB485662
G	92NG083_JV10832	NG	1992	U88826
H	VI991	BE	1993	AF190127
H	00GBAC4001	GB	2000	FJ711703
J	04CMU11421	CM	2004	GU237072
J	SE9280_7887	SE	1993	AF082394
K	97ZR-EQTB11	CD	1997	AJ249235
K	96CM-MP535	CM	1996	AJ249239
01_AE	97CNGX2F_97CNGX_2F	CN	1997	AY008714
01_AE	05GX001	CN	2005	GU564221
01_AE	CM240	TH	1990	U54771
01_AE	93TH253	TH	1993	U51189
02_AG	pBD6_15	CM	1999	AY271690
02_AG	IBNG	NG	-	L39106
07_BC	98CN009	CN	1998	AF286230
08_BC	97CNGX_6F	CN	1997	AY008715
15_01B	99TH_MU2079	TH	1999	AF516184
33_01B	05MYKL007_1	MY	2005	DQ366659
34_01B	OUR2275P	TH	1999	EF165540
48_01B	07MYKT014	MY	2007	GQ175881
51_01B	11SG_HM021	SG	2011	JN029801
52_01B	M043	TH	1996	DQ354113

53_01B	11FIR164	MY	2011	JX390610
54_01B	09MYSB023	MY	2009	JX390976
55_01B	HNCS102056	CN	2010	JX574661
58_01B	09MYPR37	MY	2009	KC522031
59_01B	09LNA423	CN	2009	JX960635
67_01B	ANHUI_MAS59	CN	2011	KC183780
68_01B	ANHUI_XC46	CN	2011	KC183783
69_01B	10JP-5091N200	JP	2010	AB845349
74_01B	10MYPR268	MY	2010	KR019771
76_01B	N628	JP	2012	†
78_cpx	YNTC19	CN	2013	KU161143

†CRF76_01B reference sequence archived in the DNA Data Bank of Japan (DDBJ) under accession number SAMD00041168-71.

d) Details of subtype B, B′, and J reference sequences (*env* gp160)

Subtype Reference	Sequence Name	Sampling Country	Sampling Year	GenBank Accession
B	8634991	AU	-	AY857144
B	03BR1046	BR	2003	JN692447
B	10BR_MG031_2	BR	2010	KT427841
B	10BR_RJ032	BR	2010	KJ849801
B	10BR_SP052	BR	2010	KT427802
B	502_1191_03	CA	2007	JF320424
B	ZEnv91_0505_12	CH	-	KU600815
B	R9ZPHI39SGA_1_R9	CH	2003	KX792557
B	14CU005	CU	2014	KR914676
B	CY067	CY	2005	FJ388911
B	CY120	CY	2005	FJ388931
B	255524	DE	2008	KT124757
B	822582	DE	2008	KT124794
B	366396	DE	2013	KT124767
B	X2886_2	ES	-	JX422209
B	ARP1195	ES	2014	KT276255
B	DEMB11FR001	FR	2011	KF716496
B	820DEN35	GB	1995	AJ535608
B	R232_F6	GB	2008	HQ595795
B	05HT_129389	HT	2005	EU839602
B	60	JP	1989	AB588232
B	DR2508	JP	2000	AB289587
B	DR7065	JP	2005	AB287368
B	KR5086_C1	KR	1995	AJ417429
B	05CSR3	KR	2005	DQ837381
B	HP-18_07JHS10-3909	KR	2007	KJ140263
B	671_00T36	NL	2000	AY423387
B	BK132	TH	1990	AY173951
B	05TH357801	TH	2005	JN248346
B	AA011a09R	TH	2006	JX446817
B	AA115c05R	TH	2008	JX448101
B	40353v04_01R	TH	2010	KU230423
B	00TT_CRC50018	TT	2000	EU839607
B	HP038C	US	-	KP754470
B	N4549TOB8U	US	-	GU728337
B	R9577TOB8U	US	-	GU728323
B	S1532TOB8U	US	-	GU728234
B	W4541TOB8U	US	-	GU728334
B	H3	US	1982	EF159971
B	WR27	US	1988	AF286365
B	M1003_x_D6	US	1994	KT283729
B	1058_11	US	1998	AY331295
B	848017	US	2002	KT124795
B	P1189_107_15_1B	US	2003	KT283923
B	1599_A1	US	2005	HQ216785
B	306227_ENV	US	2006	JX863987
B	Z95_p1	US	2006	HQ218034
B	502_0938_RH09	US	2007	JF320630
B	502_1518_RH10	US	2007	JF320099

B	502_1897_wg2	US	2007	JF320179
B	HIV_US_BID-V3046_2008	US	2008	JQ403067
B	9063_102910_CSF_1	US	2010	KM354862
B	F6817_2C7	US	2011	KU901757
B	2454	US	2014	KX505435
B	3693	US	2015	KX505552
B	2609	US	2016	KX505536
B'	RL42	CN	-	U71182
B'	YN9838	CN	1998	JF932496
B'	plwj	CN	1999	GU177863
B'	CNHN24	CN	2001	AY180905
B'	02HNsc11	CN	2002	DQ007903
B'	02HNsq4	CN	2002	DQ007902
B'	CNE1	CN	2006	HQ699949
B'	CNE4	CN	2006	HM215413
B'	CNE6	CN	2006	HM215423
B'	09YNRL215042sg	CN	2009	KC899011
B'	YN09P0014	CN	2009	JF932494
B'	C600_p1	GB	2009	JF680909
B'	JRC65B	JP	-	AB565501
B'	mSTD101	MM	1999	AB097870
B'	3156F6_A8	TH	-	KJ953339
B'	93TH067	TH	1993	U39258
B'	96TH_NP1538	TH	1996	AY713408
B'	M041	TH	1996	DQ354114
B'	M081	TH	1996	DQ354116
B'	M145	TH	1996	DQ354118
B'	99TH_C1416	TH	1999	AY945711
B'	00TH_C3198	TH	2000	AY945710
B'	3045A06_A2	TH	2000	KJ952535
B'	04TH601066	TH	2004	JN248329
B'	04TH808998	TH	2004	JN248335
B'	05TH440248	TH	2005	JN248348
B'	AA093a_RH1	TH	2006	JX447795
B'	NPBQC	TH	2006	KJ769147
B'	AA040a_WG11	TH	2007	JX447156
J	93AOHDC253	AO	1993	KU310620
J	J_97DC_KTB147	CD	1997	EF614151
J	CG-0331-02V_NGSID13	CD	2002	KY392776
J	LA26DiAn	CD	2003	KU168280
J	04CMU11421	CM	2004	GU237072
J	SE9280_7887	SE	1993	AF082394
J	SE9173_7022	SE	1994	AF082395

Table S2. Details of Asia *env* gp160 sequences downloaded from the Los Alamos National Laboratory (LANL) HIV Database and included in the present analysis

Subtype	Sampling Country	Sampling Year	Name	Risk Factor	GenBank Accession
0107	CN	2007	07JSNJ001	PWID	FJ238521
0107	CN	2007	MSM0720	MSM	KC833436
0107	CN	2007	JL070032	SG	KC990127
0107	CN	2010	JL.RF03	SH	KJ184176
0107	CN	2010	10LNA015	SH	KU051564
0107	CN	2011	JL.RF09	SH	KJ184180
0107	CN	2012	kang124-NFL	MSM	KJ778897
0107	CN	2013	BJMP3002	MSM	KM974719
0107	CN	2013	BJMP3026	MSM	KM974720
0107	CN	2014	XC2014EU09	MSM	KT592380
0107	CN	2014	GXDY460B	SH	KT619126
0107	CN	2015	zj032	N/A	KX159285
0107	CN	2015	SX15DT013	N/A	KY216146
0107	CN	2015	SX15JC06	N/A	KY216147
0107	CN	2015	SX15JC12	N/A	KY216148
0107	TW	2008	TN_H8	PWID	KT372798
0108	CN	2012	GXDY_1299_NLFG	SU	KF541292
0108	CN	2014	12YN10551	SH	KU356857
0708	CN	2000	HH069	PWID	AP005206
0708	CN	2000	HH086	PWID	AP005207
0708	CN	2008	09YN072	N/A	HQ225812
01_AE	CN	1997	97CNGX2F_97CNGX_2F	PWID	AY008714
01_AE	CN	1997	97CNGX_11F	PWID	AY008718
01_AE	CN	1999	AE01	NO	EU363849
01_AE	CN	2002	YN0203	SH	JX112860
01_AE	CN	2002	YN0221	PWID	JX112861
01_AE	CN	2002	YN0225	SH	JX112862
01_AE	CN	2002	YN0229	PWID	JX112863
01_AE	CN	2002	YN0232	SH	JX112864
01_AE	CN	2002	YN0235	SH	JX112865
01_AE	CN	2002	YN0236	SH	JX112866
01_AE	CN	2005	FJ051	SH	DQ859178
01_AE	CN	2005	FJ053	SH	DQ859179
01_AE	CN	2005	Fj055	SH	EF036527
01_AE	CN	2005	Fj052	PWID	EF036528
01_AE	CN	2005	Fj056	SH	EF036529
01_AE	CN	2005	Fj057	SH	EF036530
01_AE	CN	2005	Fj065	SH	EF036534
01_AE	CN	2005	Fj066	SH	EF036535
01_AE	CN	2005	AE03_SH188_6	SU	EU363851
01_AE	CN	2005	05GX034	SH	GQ845124
01_AE	CN	2005	05GX079	SH	GQ845125
01_AE	CN	2005	05GX128	SH	GQ845126
01_AE	CN	2005	05GX001	SH	GU564221
01_AE	CN	2005	05GX002	SH	GU564222
01_AE	CN	2005	05GX012	SH	GU564223
01_AE	CN	2005	05GX013	PWID	GU564224
01_AE	CN	2005	05GX014	SH	GU564225
01_AE	CN	2005	05GX142	SH	GU564227
01_AE	CN	2005	05GX156	PWID	GU564228
01_AE	CN	2005	05GX162	SH	GU564229
01_AE	CN	2005	CNE62	MSM	HM215424
01_AE	CN	2005	CNE60	N/A	HQ699977
01_AE	CN	2005	CNE61_U	N/A	HQ699987
01_AE	CN	2005	GX2005002	SH	KP178420
01_AE	CN	2006	FJ054	SH	DQ859180
01_AE	CN	2006	Fj062	SH	EF036531
01_AE	CN	2006	Fj063	SH	EF036532
01_AE	CN	2006	Fj064	SH	EF036533
01_AE	CN	2006	Fj061	SH	EF036536
01_AE	CN	2006	AE02	PWID	EU363850
01_AE	CN	2006	HuB199_1	N/A	GU475025

01_AE	CN	2006	YN192_31	N/A	GU475046
01_AE	CN	2006	06GX239	SH	GU564230
01_AE	CN	2006	CNE3	PWID	HM215410
01_AE	CN	2006	CNE5	SH	HM215415
01_AE	CN	2006	CNE59	PWID	HM215422
01_AE	CN	2006	CNE8	PWID	HM215427
01_AE	CN	2006	CNE91_U	N/A	HQ699993
01_AE	CN	2006	SC070062	PWID	JX112857
01_AE	CN	2006	SC070064	SH	JX112858
01_AE	CN	2007	07JSWX045	PWID	FJ441290
01_AE	CN	2007	GX24_8	N/A	GU475015
01_AE	CN	2007	GX28_31	N/A	GU475017
01_AE	CN	2007	BJX4_6	N/A	GU475020
01_AE	CN	2007	GX34_21	N/A	GU475022
01_AE	CN	2007	BJ5_11	N/A	GU475024
01_AE	CN	2007	GX2010_36	N/A	GU475026
01_AE	CN	2007	BJ17A_6	N/A	GU475028
01_AE	CN	2007	GX25_29	N/A	GU475030
01_AE	CN	2007	GX142_2	N/A	GU475031
01_AE	CN	2007	GZ187_10	N/A	GU475036
01_AE	CN	2007	GX13_7	N/A	GU475037
01_AE	CN	2007	BJ3_4	N/A	GU475040
01_AE	CN	2007	GX11_13	N/A	GU475042
01_AE	CN	2007	GX155_55	N/A	GU475043
01_AE	CN	2007	GX54_6	N/A	GU475045
01_AE	CN	2007	BJOX005000_09_2	MSM	HM215367
01_AE	CN	2007	BJOX008000_04_3	SH	HM215371
01_AE	CN	2007	BJOX009000_02_4	MSM	HM215372
01_AE	CN	2007	BJOX010000_06_2	MSM	HM215373
01_AE	CN	2007	BJOX012000_04_1	MSM	HM215376
01_AE	CN	2007	BJOX015000_11_5	MSM	HM215377
01_AE	CN	2007	BJOX017000_07_1	MSM	HM215378
01_AE	CN	2007	BJOX018000_02_3	MSM	HM215379
01_AE	CN	2007	BJOX021000_03_3	MSM	HM215383
01_AE	CN	2007	BJOX023000_05_4	MSM	HM215385
01_AE	CN	2007	BJOX025000_01_1	MSM	HM215386
01_AE	CN	2007	BJOX028000_10_3	MSM	HM215389
01_AE	CN	2007	BJOX030000_02_5	SH	HM215393
01_AE	CN	2007	CNE28	PWID	HM215409
01_AE	CN	2007	CNE55	PWID	HM215418
01_AE	CN	2007	CNE56	PWID	HM215419
01_AE	CN	2007	CNE26	N/A	HQ699955
01_AE	CN	2007	CNE27	N/A	HQ699956
01_AE	CN	2007	CNE107	PWID	HQ699986
01_AE	CN	2007	CNE71_U	N/A	HQ699988
01_AE	CN	2007	FJ070010	SH	JX112809
01_AE	CN	2007	FJ070013	SH	JX112810
01_AE	CN	2007	FJ070017	SH	JX112811
01_AE	CN	2007	FJ070033	N/A	JX112813
01_AE	CN	2007	FJ070035	SH	JX112814
01_AE	CN	2007	FJ070037	SH	JX112815
01_AE	CN	2007	FJ070039	SH	JX112816
01_AE	CN	2007	FJ070040	SH	JX112817
01_AE	CN	2007	FJ070043	SH	JX112818
01_AE	CN	2007	GD070010	SH	JX112819
01_AE	CN	2007	GD070058	SH	JX112820
01_AE	CN	2007	GD070059	SH	JX112821
01_AE	CN	2007	GD070083	SH	JX112822
01_AE	CN	2007	GD070090	PWID	JX112823
01_AE	CN	2007	GD070092	PWID	JX112824
01_AE	CN	2007	GD070096	PWID	JX112825
01_AE	CN	2007	GD070118	PWID	JX112826
01_AE	CN	2007	GD070120	N/A	JX112827
01_AE	CN	2007	GD070176	PWID	JX112828
01_AE	CN	2007	GX070003	SH	JX112829
01_AE	CN	2007	GX070005	SH	JX112830

01_AE	CN	2007	GX070006	PWID	JX112831
01_AE	CN	2007	GX070043	SH	JX112832
01_AE	CN	2007	GX070044	SH	JX112833
01_AE	CN	2007	GX070076	SH	JX112834
01_AE	CN	2007	GX070143	SH	JX112835
01_AE	CN	2007	GX070145	SH	JX112836
01_AE	CN	2007	GX070149	PWID	JX112837
01_AE	CN	2007	GX070154	PWID	JX112838
01_AE	CN	2007	GX070167	PWID	JX112839
01_AE	CN	2007	GZ070004	PWID	JX112840
01_AE	CN	2007	GZ070015	N/A	JX112841
01_AE	CN	2007	GZ070016	PWID	JX112842
01_AE	CN	2007	GZ070123	SH	JX112843
01_AE	CN	2007	GZ070126	N/A	JX112844
01_AE	CN	2007	GZ070127	SH	JX112845
01_AE	CN	2007	JS070901	SH	JX112850
01_AE	CN	2007	JS071001	SH	JX112851
01_AE	CN	2007	JS071004	SH	JX112852
01_AE	CN	2007	JS071101	SH	JX112853
01_AE	CN	2007	LN070008	MSM	JX112854
01_AE	CN	2007	LN070010	MSM	JX112855
01_AE	CN	2007	LN070013	SH	JX112856
01_AE	CN	2007	TJ070003	MSM	JX112859
01_AE	CN	2007	07CNYN312	MB	KF835499
01_AE	CN	2007	07CNYN315	SU	KF835502
01_AE	CN	2007	07CNYN316	SU	KF835503
01_AE	CN	2007	07CNYN317	N/A	KF835504
01_AE	CN	2007	07CNYN318	MB	KF835505
01_AE	CN	2007	07CNYN326	SU	KF835513
01_AE	CN	2007	07CNYN327	PWID	KF835514
01_AE	CN	2007	07CNYN329	N/A	KF835516
01_AE	CN	2007	07CNYN332	SU	KF835518
01_AE	CN	2007	07CNYN333	SU	KF835519
01_AE	CN	2007	07CNYN337	MB	KF835523
01_AE	CN	2007	07CNYN342	SU	KF835527
01_AE	CN	2007	07CNYN343	N/A	KF835528
01_AE	CN	2007	07CNYN354	SU	KF835533
01_AE	CN	2007	07CNYN357	SU	KF835536
01_AE	CN	2007	07CNYN359	SU	KF835538
01_AE	CN	2007	07CNYN364	SU	KF835542
01_AE	CN	2007	07CNYN366	SU	KF835543
01_AE	CN	2008	GX91_2	N/A	GU475013
01_AE	CN	2008	GX81_43	N/A	GU475014
01_AE	CN	2008	GX71_18	N/A	GU475016
01_AE	CN	2008	GX88_47	N/A	GU475018
01_AE	CN	2008	GX90_1	N/A	GU475019
01_AE	CN	2008	GX74_20	N/A	GU475021
01_AE	CN	2008	GX35_33	N/A	GU475023
01_AE	CN	2008	GX83_47	N/A	GU475027
01_AE	CN	2008	BJ6_17	N/A	GU475029
01_AE	CN	2008	SH6_81	N/A	GU475032
01_AE	CN	2008	SHX335_24	N/A	GU475033
01_AE	CN	2008	SHX346_60	N/A	GU475034
01_AE	CN	2008	GX72_27	N/A	GU475035
01_AE	CN	2008	GX73_29	N/A	GU475038
01_AE	CN	2008	GX8C_31	N/A	GU475039
01_AE	CN	2008	GX68_5	N/A	GU475044
01_AE	CN	2008	BJOX031000_02_2	MSM	HM215394
01_AE	CN	2008	08LNA003	MSM	JX960606
01_AE	CN	2008	08LNA002	MSM	JX960612
01_AE	CN	2008	08LNA004	MSM	JX960617
01_AE	CN	2008	BJOX033000.e01	SH	KM218302
01_AE	CN	2009	1119	MSM	HQ215553
01_AE	CN	2009	1109	MSM	HQ215555
01_AE	CN	2009	YN09P0011	SH	JX112867
01_AE	CN	2009	YN09P0015	SH	JX112868

01_AE	CN	2009	ZK052	N/A	JX112869
01_AE	CN	2009	ZK056	N/A	JX112870
01_AE	CN	2009	09LNA379	MSM	JX960603
01_AE	CN	2009	09LNA008	MSM	JX960604
01_AE	CN	2009	09LNA011	MSM	JX960605
01_AE	CN	2009	09LNA340	MSM	JX960607
01_AE	CN	2009	09LNA020	MSM	JX960613
01_AE	CN	2009	09LNA230	MSM	JX960614
01_AE	CN	2009	09LNA040	MSM	JX960615
01_AE	CN	2009	09LNA041	MSM	JX960618
01_AE	CN	2009	09LNA425	MSM	JX960621
01_AE	CN	2009	09LNA013	MSM	JX960623
01_AE	CN	2009	09LNA527	MSM	JX960625
01_AE	CN	2009	10LNA105	MSM	JX960626
01_AE	CN	2009	09LNA007	MSM	JX960627
01_AE	CN	2009	09LNA353	MSM	JX960628
01_AE	CN	2009	10LNA016	MSM	JX960629
01_AE	CN	2009	09LNA005	MSM	JX960630
01_AE	CN	2009	09LNA136	MSM	JX960631
01_AE	CN	2009	09LNA025	MSM	JX960634
01_AE	CN	2009	09LNA480	MSM	JX960636
01_AE	CN	2009	09LNA009	MSM	JX960638
01_AE	CN	2009	09LNA086	MSM	JX960639
01_AE	CN	2009	DE00109CN005	MSM	KP109504
01_AE	CN	2009	DE00109CN008	MSM	KP109505
01_AE	CN	2010	CYM059	MSM	JX112796
01_AE	CN	2010	CYM075	MSM	JX112797
01_AE	CN	2010	CYM105	MSM	JX112798
01_AE	CN	2010	CYM124	MSM	JX112799
01_AE	CN	2010	CYM136	MSM	JX112800
01_AE	CN	2010	CYM138	MSM	JX112801
01_AE	CN	2010	CYM139	MSM	JX112802
01_AE	CN	2010	CYM140	MSM	JX112803
01_AE	CN	2010	CYM143	MSM	JX112804
01_AE	CN	2010	CYM147	MSM	JX112805
01_AE	CN	2010	CYM149	MSM	JX112806
01_AE	CN	2010	CYM152	MSM	JX112807
01_AE	CN	2010	CYM154	MSM	JX112808
01_AE	CN	2010	JL100005	SH	JX112846
01_AE	CN	2010	JL100007	MSM	JX112847
01_AE	CN	2010	JL100014	SH	JX112848
01_AE	CN	2010	JL100020	MSM	JX112849
01_AE	CN	2010	10LNA103	MSM	JX960608
01_AE	CN	2010	10LNA124	MSM	JX960609
01_AE	CN	2010	10LNA819	MSM	JX960610
01_AE	CN	2010	10LNA571	MSM	JX960611
01_AE	CN	2010	10LNA821	MSM	JX960616
01_AE	CN	2010	10LNA294	MSM	JX960619
01_AE	CN	2010	10LNA918	MSM	JX960620
01_AE	CN	2010	10LNA264	MSM	JX960622
01_AE	CN	2010	10LNA669	MSM	JX960624
01_AE	CN	2010	10LNA976	MSM	JX960632
01_AE	CN	2010	10LNA057	MSM	JX960633
01_AE	CN	2010	10LNA471	MSM	JX960637
01_AE	CN	2010	YNFL03	SH	KC870029
01_AE	CN	2010	YNFL20	SH	KC870039
01_AE	CN	2010	YNFL23	SG	KC870041
01_AE	CN	2010	DE00110CN007	MSM	KP109506
01_AE	CN	2010	DE00110CN009	MSM	KP109507
01_AE	CN	2011	DE00111CN002	MSM	KC596064
01_AE	CN	2011	DE00111CN003	MSM	KC596065
01_AE	CN	2012	DE00112CN011	MSM	KP109508
01_AE	CN	-	GX_E_14	N/A	AY217545
01_AE	HK	2004	HK001	N/A	DQ234790
01_AE	ID	1993	ID17	N/A	AB485652
01_AE	JP	1993	93JP_NH1	SH	AB052995

01_AE	JP	1993	NH25_93JPNH25T_93JP_NH2_5T	SH	AB070352
01_AE	JP	2011	DE00111JP003	N/A	KF859741
01_AE	JP	-	DR0492	N/A	AB253423
01_AE	JP	-	DR6824	N/A	AB253426
01_AE	JP	-	DR1741	N/A	AB253635
01_AE	JP	-	DR1873	N/A	AB253647
01_AE	JP	-	DR2594	N/A	AB253659
01_AE	JP	-	DR1236	N/A	AB253692
01_AE	JP	-	DR2192	N/A	AB253703
01_AE	JP	-	JRC77AE	N/A	AB565503
01_AE	KH	-	C1712	N/A	DQ518410
01_AE	KH	-	LEANG	N/A	DQ518411
01_AE	MM	1999	mCSW105	SW	AB097872
01_AE	MM	2014	fKSDU26	PWID	KU820849
01_AE	NP	2011	11NP084	SH	KJ541845
01_AE	SG	2008	HM086	N/A	KY213716
01_AE	SG	2008	HM097	N/A	KY213717
01_AE	SG	2008	HM073	N/A	KY213719
01_AE	SG	2008	FREE019	N/A	KY213720
01_AE	SG	2008	HM033	N/A	KY213722
01_AE	SG	2008	HM051	N/A	KY213723
01_AE	SG	2008	HM089	N/A	KY213724
01_AE	SG	2008	HM043	N/A	KY213725
01_AE	SG	2008	HM067	N/A	KY213726
01_AE	SG	2008	HM040	N/A	KY213727
01_AE	SG	2008	HM044	N/A	KY213728
01_AE	SG	2008	HM018	N/A	KY213730
01_AE	SG	2008	HM026	N/A	KY213731
01_AE	SG	2008	HM108	N/A	KY213732
01_AE	SG	2008	HM032	N/A	KY213733
01_AE	SG	2008	HM019	N/A	KY213735
01_AE	SG	2008	HM034	N/A	KY213736
01_AE	SG	2008	HM045	N/A	KY213737
01_AE	SG	2008	HM038	N/A	KY213738
01_AE	SG	2008	HM069	N/A	KY213739
01_AE	SG	2009	HM130	N/A	KY213715
01_AE	SG	2009	HM123D	N/A	KY213718
01_AE	SG	2009	HM153I	N/A	KY213721
01_AE	SG	2009	HM132F	N/A	KY213729
01_AE	SG	2009	HM149	N/A	KY213734
01_AE	SG	2009	HM140	N/A	KY213749
01_AE	TH	1990	CM235	SH	AF259954
01_AE	TH	1990	90TH_CM244	N/A	AY713425
01_AE	TH	1990	90TH_CM240	SH	AY736838
01_AE	TH	1990	CM246_c1	SH	JN944663
01_AE	TH	1992	92TH001	SH	AY494968
01_AE	TH	1992	TH023	SH	KU562843
01_AE	TH	1993	93TH051	N/A	AB220944
01_AE	TH	1993	93TH054	N/A	AB220945
01_AE	TH	1993	93TH060	N/A	AB220946
01_AE	TH	1993	93TH062	N/A	AB220947
01_AE	TH	1993	93TH065	PWID	AB220948
01_AE	TH	1993	93TH057	PWID	AB253424
01_AE	TH	1993	93TH9021	N/A	AF164485
01_AE	TH	1993	C2_CMU02	SH	AY494967
01_AE	TH	1993	KH03	N/A	U48264
01_AE	TH	1993	KH08	N/A	U48266
01_AE	TH	1993	93TH253	SH	U51189
01_AE	TH	1994	A01021_A8_1	SW	AF015916
01_AE	TH	1994	E11429_A3_1	N/A	AF015919
01_AE	TH	1994	VI1249	N/A	DL258716
01_AE	TH	1995	95TNIH022	SH	AB032740
01_AE	TH	1995	95TNIH047	SH	AB032741
01_AE	TH	1995	NI1144	SH	AF070703
01_AE	TH	1995	NI1145	SH	AF070704
01_AE	TH	1995	NI1146	N/A	AF070705

01_AE	TH	1995	NI1149	N/A	AF070707
01_AE	TH	1995	NI1150	SU	AF070708
01_AE	TH	1995	NI1152	SH	AF070709
01_AE	TH	1996	NI1154	SH	AF070710
01_AE	TH	1996	NI1155	SH	AF070711
01_AE	TH	1996	NI1157	SU	AF070713
01_AE	TH	1996	96TH_NI1046	N/A	AY713421
01_AE	TH	1996	96TH_M02138	N/A	AY713424
01_AE	TH	1996	M114	SH	DQ354117
01_AE	TH	1996	M066_07	SH	JN944664
01_AE	TH	1997	97TH6_107	N/A	AY125894
01_AE	TH	1997	97TH_NP1695	N/A	AY713419
01_AE	TH	1997	97TH_NP1525	N/A	AY713420
01_AE	TH	1998	98TH_NP1251	N/A	AY713422
01_AE	TH	1998	98TH_R1166	SH	AY945728
01_AE	TH	1999	OUR199I	PWID	AY358039
01_AE	TH	1999	OUR066I	PWID	AY358043
01_AE	TH	1999	OUR098I	PWID	AY358044
01_AE	TH	1999	OUR164I	PWID	AY358045
01_AE	TH	1999	OUR202I	PWID	AY358047
01_AE	TH	1999	OUR203I	PWID	AY358048
01_AE	TH	1999	OUR258I	PWID	AY358049
01_AE	TH	1999	OUR422I	PWID	AY358051
01_AE	TH	1999	OUR008I	N/A	AY358065
01_AE	TH	1999	99TH_NI1052	N/A	AY713423
01_AE	TH	1999	99TH_C1080	SH	AY945712
01_AE	TH	1999	99TH_C2405	N/A	AY945718
01_AE	TH	1999	99TH_C4460	N/A	AY945726
01_AE	TH	1999	99TH_R1149	N/A	AY945727
01_AE	TH	1999	99TH_R3006	N/A	AY945731
01_AE	TH	1999	99TH_R3265	SH	AY945732
01_AE	TH	1999	3022A02.A1	N/A	KJ952376
01_AE	TH	1999	3025A02.A3	N/A	KJ952432
01_AE	TH	1999	3067A08.A10	N/A	KJ952689
01_AE	TH	1999	3078A02.E16	N/A	KJ952712
01_AE	TH	1999	3084A16.A1	N/A	KJ952732
01_AE	TH	1999	3125A12.D15	N/A	KJ952991
01_AE	TH	1999	3151A16.A2	N/A	KJ953229
01_AE	TH	1999	3162A10.A3	N/A	KJ953366
01_AE	TH	1999	3184A08.B1	N/A	KJ953408
01_AE	TH	1999	3186A14.A2	N/A	KJ953438
01_AE	TH	1999	3193A14.C1	N/A	KJ953503
01_AE	TH	1999	3210A08.D22	N/A	KJ953596
01_AE	TH	1999	3212A10.B10	N/A	KJ953611
01_AE	TH	2000	OUR201I	PWID	AY358046
01_AE	TH	2000	OUR595I	PWID	AY358052
01_AE	TH	2000	OUR661I	PWID	AY358057
01_AE	TH	2000	OUR724I	PWID	AY358060
01_AE	TH	2000	OUR746I	PWID	AY358061
01_AE	TH	2000	OUR810I	PWID	AY358063
01_AE	TH	2000	OUR200I	PWID	AY358066
01_AE	TH	2000	OUR721I	PWID	AY358067
01_AE	TH	2000	00TH_C2101	SH	AY945716
01_AE	TH	2000	00TH_C2257	N/A	AY945717
01_AE	TH	2000	00TH_C3347	SH	AY945721
01_AE	TH	2000	00TH_C4118	SH	AY945722
01_AE	TH	2000	00TH_C4151	N/A	AY945724
01_AE	TH	2000	00TH_C4382	N/A	AY945725
01_AE	TH	2000	C1705	N/A	DQ789392
01_AE	TH	2000	3002B01.A12	N/A	KJ952242
01_AE	TH	2000	3019A06.C1	N/A	KJ952346
01_AE	TH	2000	3043A03.B11	N/A	KJ952505
01_AE	TH	2000	3046A02.A1	N/A	KJ952562
01_AE	TH	2000	3063A10.B1	N/A	KJ952667
01_AE	TH	2000	3090A02.C6	N/A	KJ952765
01_AE	TH	2000	3104A06.C4	N/A	KJ952852

01_AE	TH	2000	3111A02.A2	N/A	KJ952897
01_AE	TH	2000	3112A02.A1	N/A	KJ952917
01_AE	TH	2000	3118A16.A1	N/A	KJ952968
01_AE	TH	2000	3131A12.C11	N/A	KJ953089
01_AE	TH	2000	3135A10.A11	N/A	KJ953119
01_AE	TH	2000	3153A14.A1	N/A	KJ953282
01_AE	TH	2000	3189A08.A2	N/A	KJ953459
01_AE	TH	2000	3203A14.A1	N/A	KJ953555
01_AE	TH	2000	3218A16.A1	N/A	KJ953634
01_AE	TH	2000	3219A08.D1	N/A	KJ953664
01_AE	TH	2001	OUR786I	PWID	AY358036
01_AE	TH	2001	OUR674I	PWID	AY358038
01_AE	TH	2001	OUR609I	PWID	AY358040
01_AE	TH	2001	OUR642I	PWID	AY358041
01_AE	TH	2001	OUR414I	N/A	AY358050
01_AE	TH	2001	OUR647I	PWID	AY358056
01_AE	TH	2001	OUR702I	PWID	AY358059
01_AE	TH	2001	OUR830I	PWID	AY358064
01_AE	TH	2001	OUR788I	PWID	AY358068
01_AE	TH	2001	01TH_C1436	N/A	AY945713
01_AE	TH	2001	01TH_C2570	N/A	AY945719
01_AE	TH	2001	01TH_C3256	N/A	AY945720
01_AE	TH	2001	01TH_R2184	SH	AY945730
01_AE	TH	2002	OUR737I	PWID	AY358037
01_AE	TH	2002	OUR769I	PWID	AY358062
01_AE	TH	2003	TH7229	N/A	KU168309
01_AE	TH	2004	BKD	N/A	DQ314731
01_AE	TH	2004	BKM	N/A	DQ314732
01_AE	TH	2004	T500617_sga02	N/A	HQ691003
01_AE	TH	2004	T502281_sga02	N/A	HQ691013
01_AE	TH	2004	T276248_sga01	N/A	JF297221
01_AE	TH	2004	04TH107542	N/A	JN248318
01_AE	TH	2004	04TH328531	N/A	JN248324
01_AE	TH	2004	04TH427990	N/A	JN248327
01_AE	TH	2004	04TH505841	N/A	JN248328
01_AE	TH	2004	04TH613543	N/A	JN248330
01_AE	TH	2004	04TH807015	N/A	JN248334
01_AE	TH	2004	04TH817196	N/A	JN248336
01_AE	TH	2004	AA027a_wg1	N/A	JX447018
01_AE	TH	2004	AA074a07R	N/A	JX447529
01_AE	TH	2004	AA075a_WG10	N/A	JX447535
01_AE	TH	2004	T256254_01	N/A	KC748972
01_AE	TH	2005	T293735_sga06	N/A	HQ690964
01_AE	TH	2005	05TH127331	N/A	JN248338
01_AE	TH	2005	05TH130087	N/A	JN248339
01_AE	TH	2005	05TH327568	N/A	JN248341
01_AE	TH	2005	05TH342968	N/A	JN248342
01_AE	TH	2005	05TH741452	N/A	JN248355
01_AE	TH	2005	05TH841749	N/A	JN248356
01_AE	TH	2005	356272_c02	SH	JN944654
01_AE	TH	2005	620345_c10	SH	JN944656
01_AE	TH	2005	703357_c02	SH	JN944658
01_AE	TH	2005	AA004a_WG12	N/A	JX446710
01_AE	TH	2005	AA023a10R	N/A	JX446976
01_AE	TH	2005	AA029b01R	N/A	JX447043
01_AE	TH	2005	AA033a_wg1	N/A	JX447077
01_AE	TH	2005	AA049a_WG11	N/A	JX447266
01_AE	TH	2005	AA051a01R	N/A	JX447294
01_AE	TH	2005	AA062b07R	N/A	JX447395
01_AE	TH	2005	AA064a_WG1	N/A	JX447412
01_AE	TH	2005	AA066a09R	N/A	JX447447
01_AE	TH	2005	AA077a_RH10	N/A	JX447561
01_AE	TH	2005	AA078a11R	N/A	JX447579
01_AE	TH	2005	AA079a_WG1	N/A	JX447589
01_AE	TH	2005	AA094b05R	N/A	JX447814
01_AE	TH	2005	AA097a01R	N/A	JX447853

01_AE	TH	2005	AA101a_RH1	N/A	JX447921
01_AE	TH	2005	AA103a06R	N/A	JX447958
01_AE	TH	2005	AA107a_wg1	N/A	JX448019
01_AE	TH	2005	AA122a02R	N/A	JX448217
01_AE	TH	2005	AA125a12R	N/A	JX448253
01_AE	TH	2005	AA126a06R	N/A	JX448270
01_AE	TH	2006	21PL2	N/A	EU743757
01_AE	TH	2006	22PL1	N/A	EU743758
01_AE	TH	2006	29CC1	N/A	EU743759
01_AE	TH	2006	41CC1	N/A	EU743763
01_AE	TH	2006	41PB3	N/A	EU743764
01_AE	TH	2006	45CC1	N/A	EU743765
01_AE	TH	2006	45PB1	N/A	EU743766
01_AE	TH	2006	47CC11	N/A	EU743767
01_AE	TH	2006	47PL1	N/A	EU743768
01_AE	TH	2006	50PB2	N/A	EU743769
01_AE	TH	2006	50PL1	N/A	EU743770
01_AE	TH	2006	52PB3	N/A	EU743771
01_AE	TH	2006	52PL4	N/A	EU743772
01_AE	TH	2006	52PL7	N/A	EU743773
01_AE	TH	2006	55PL1	N/A	EU743774
01_AE	TH	2006	60CC3	N/A	EU743775
01_AE	TH	2006	60PB2	N/A	EU743776
01_AE	TH	2006	60PL2	N/A	EU743777
01_AE	TH	2006	62PL1	N/A	EU743778
01_AE	TH	2006	65CC1	N/A	EU743779
01_AE	TH	2006	65CC4	N/A	EU743780
01_AE	TH	2006	65PL1	N/A	EU743781
01_AE	TH	2006	98CC2	N/A	EU743782
01_AE	TH	2006	98CC3	N/A	EU743783
01_AE	TH	2006	98PB2	N/A	EU743784
01_AE	TH	2006	99CC8	N/A	EU743785
01_AE	TH	2006	99PB2	N/A	EU743786
01_AE	TH	2006	99PL2	N/A	EU743787
01_AE	TH	2006	101PL1	N/A	EU743788
01_AE	TH	2006	102CC2	N/A	EU743789
01_AE	TH	2006	104PB4	N/A	EU743790
01_AE	TH	2006	105PB1	N/A	EU743791
01_AE	TH	2006	105PL2	N/A	EU743792
01_AE	TH	2006	105PL3	N/A	EU743793
01_AE	TH	2006	107CC2	N/A	EU743794
01_AE	TH	2006	T614109_sga02	N/A	HQ691082
01_AE	TH	2006	T501602_sga01	N/A	JF297225
01_AE	TH	2006	427299_c12	SH	JN944655
01_AE	TH	2006	644039_c01b	SH	JN944657
01_AE	TH	2006	816763_c02	SH	JN944659
01_AE	TH	2006	AA002a_WG1	N/A	JX446666
01_AE	TH	2006	AA003b05R	N/A	JX446698
01_AE	TH	2006	AA006a02	N/A	JX446736
01_AE	TH	2006	AA014a05	N/A	JX446853
01_AE	TH	2006	AA017a_wg1	N/A	JX446899
01_AE	TH	2006	AA018a07R	N/A	JX446910
01_AE	TH	2006	AA022a_RH1	N/A	JX446960
01_AE	TH	2006	AA034a_wg1	N/A	JX447087
01_AE	TH	2006	AA035a01R	N/A	JX447102
01_AE	TH	2006	AA038a_WG3	N/A	JX447132
01_AE	TH	2006	AA042a12R	N/A	JX447199
01_AE	TH	2006	AA044a_RH1	N/A	JX447220
01_AE	TH	2006	AA055a_WG4	N/A	JX447312
01_AE	TH	2006	AA056a_WG2	N/A	JX447315
01_AE	TH	2006	AA058a02R	N/A	JX447344
01_AE	TH	2006	AA059a_WG1	N/A	JX447349
01_AE	TH	2006	AA063a_RH1	N/A	JX447402
01_AE	TH	2006	AA068a_14	N/A	JX447465
01_AE	TH	2006	AA073a_RH1	N/A	JX447515
01_AE	TH	2006	AA076a03R	N/A	JX447550

01_AE	TH	2006	AA082a_WG1	N/A	JX447637
01_AE	TH	2006	AA083a08R	N/A	JX447657
01_AE	TH	2006	AA085a_wg1	N/A	JX447678
01_AE	TH	2006	AA086a06R	N/A	JX447699
01_AE	TH	2006	AA088a_wg11	N/A	JX447709
01_AE	TH	2006	AA099a_WG1	N/A	JX447884
01_AE	TH	2006	AA100b07R	N/A	JX447902
01_AE	TH	2006	AA104a_RH1	N/A	JX447978
01_AE	TH	2006	AA109a01R	N/A	JX448039
01_AE	TH	2006	AA121a04R	N/A	JX448198
01_AE	TH	2006	AA123a04R	N/A	JX448238
01_AE	TH	2006	AA127a02R	N/A	JX448279
01_AE	TH	2006	AA129a02R	N/A	JX448289
01_AE	TH	2006	AA130a07R	N/A	JX448301
01_AE	TH	2007	WN04	N/A	GQ916569
01_AE	TH	2007	WN10	N/A	GQ916571
01_AE	TH	2007	WN12	N/A	GQ916572
01_AE	TH	2007	WN13	N/A	GQ916573
01_AE	TH	2007	WN26	N/A	GQ916574
01_AE	TH	2007	WN27	N/A	GQ916575
01_AE	TH	2007	T357545_sga01	N/A	HQ690973
01_AE	TH	2007	T502102_sga02	N/A	HQ691004
01_AE	TH	2007	T503006_sga10	N/A	HQ691020
01_AE	TH	2007	T509989_sga11	N/A	HQ691039
01_AE	TH	2007	T518020_sga08	N/A	HQ691052
01_AE	TH	2007	T535902_sga02	N/A	HQ691073
01_AE	TH	2007	MERLBDTRC2	N/A	JN860761
01_AE	TH	2007	MERLBDTRC4	N/A	JN860763
01_AE	TH	2007	MERLBDTRC5	N/A	JN860764
01_AE	TH	2007	MERLBDTRC7	N/A	JN860766
01_AE	TH	2007	MERLBDTRC8	N/A	JN860767
01_AE	TH	2007	AA005a07R	N/A	JX446728
01_AE	TH	2007	AA008a_RH1	N/A	JX446775
01_AE	TH	2007	AA009b01R	N/A	JX446790
01_AE	TH	2007	AA012a_RH1	N/A	JX446837
01_AE	TH	2007	AA015a_RH5	N/A	JX446873
01_AE	TH	2007	AA019a_WG1	N/A	JX446916
01_AE	TH	2007	AA021a08R	N/A	JX446942
01_AE	TH	2007	AA024a10	N/A	JX446987
01_AE	TH	2007	AA026a02R	N/A	JX447013
01_AE	TH	2007	AA028a_wg3	N/A	JX447028
01_AE	TH	2007	AA030a08	N/A	JX447054
01_AE	TH	2007	AA031a01	N/A	JX447063
01_AE	TH	2007	AA036a01R	N/A	JX447117
01_AE	TH	2007	AA041a01R	N/A	JX447177
01_AE	TH	2007	AA048b08R	N/A	JX447252
01_AE	TH	2007	AA050a_WG7	N/A	JX447283
01_AE	TH	2007	AA061a05R	N/A	JX447379
01_AE	TH	2007	AA065a01R	N/A	JX447427
01_AE	TH	2007	AA069a01R	N/A	JX447480
01_AE	TH	2007	AA072a09	N/A	JX447499
01_AE	TH	2007	AA080a09R	N/A	JX447608
01_AE	TH	2007	AA089a05	N/A	JX447721
01_AE	TH	2007	AA091a02R	N/A	JX447746
01_AE	TH	2007	AA092b13R	N/A	JX447773
01_AE	TH	2007	AA098a08R	N/A	JX447875
01_AE	TH	2007	AA105a06R	N/A	JX448001
01_AE	TH	2008	TR01	N/A	GQ916576
01_AE	TH	2008	TR09	N/A	GQ916577
01_AE	TH	2008	TR18	N/A	GQ916578
01_AE	TH	2008	TR23	N/A	GQ916579
01_AE	TH	2008	TR26	N/A	GQ916580
01_AE	TH	2008	TR27	N/A	GQ916581
01_AE	TH	2008	TR28	N/A	GQ916582
01_AE	TH	2008	TR32	N/A	GQ916583
01_AE	TH	2008	RY05	N/A	GQ916584

01_AE	TH	2008	RY09	N/A	GQ916585
01_AE	TH	2008	RY12	N/A	GQ916587
01_AE	TH	2008	RY17	N/A	GQ916588
01_AE	TH	2008	RY18	N/A	GQ916589
01_AE	TH	2008	RY25	N/A	GQ916590
01_AE	TH	2008	RY27	N/A	GQ916591
01_AE	TH	2008	RY31	N/A	GQ916592
01_AE	TH	2008	RY37	N/A	GQ916593
01_AE	TH	2008	T504258_sga07	N/A	HQ691028
01_AE	TH	2008	AE_Env_CR2_Apr08D	N/A	JN388081
01_AE	TH	2008	AE_Env_CR3_Apr08R	N/A	JN388091
01_AE	TH	2008	AE_Env_CR10_Apr08D	N/A	JN388101
01_AE	TH	2008	AE_Env_CR14_Apr08R	N/A	JN388111
01_AE	TH	2008	AE_Env_CR15_Apr08R	N/A	JN388121
01_AE	TH	2008	AE_Env_CR8_Apr08R	N/A	JN388131
01_AE	TH	2008	AE_Env_CR11_Apr08R	N/A	JN388141
01_AE	TH	2008	AE_Env_CR12_Apr08R	N/A	JN388151
01_AE	TH	2008	AE_Env_CR17_Apr08R	N/A	JN388161
01_AE	TH	2008	AE_Env_CR19_Apr08D	N/A	JN388171
01_AE	TH	2008	AE_Env_CR25_Apr08D	N/A	JN388181
01_AE	TH	2008	AE_Env_CR28_Apr08D	N/A	JN388191
01_AE	TH	2008	AE_Env_CR29_Apr08D	N/A	JN388201
01_AE	TH	2008	AE_Env_CR36_Apr08D	N/A	JN388211
01_AE	TH	2008	AE_Env_CR38_Apr08D	N/A	JN388221
01_AE	TH	2008	MERLBDTRC9	N/A	JN860768
01_AE	TH	2008	AA001a07R	N/A	JX446655
01_AE	TH	2008	AA007a_WG1	N/A	JX446755
01_AE	TH	2008	AA013a05	N/A	JX446848
01_AE	TH	2008	AA016a14R	N/A	JX446888
01_AE	TH	2008	AA032a01	N/A	JX447072
01_AE	TH	2008	AA037A_WG11	N/A	JX447122
01_AE	TH	2008	AA052a01	N/A	JX447300
01_AE	TH	2008	AA054a05	N/A	JX447305
01_AE	TH	2008	AA057a02R	N/A	JX447328
01_AE	TH	2008	AA060a_WG1	N/A	JX447359
01_AE	TH	2008	AA067A_WG17	N/A	JX447454
01_AE	TH	2008	AA070a_RH01	N/A	JX447493
01_AE	TH	2008	AA102a_RH1	N/A	JX447941
01_AE	TH	2008	AA108a_WG6	N/A	JX448028
01_AE	TH	2008	AA110a01R	N/A	JX448051
01_AE	TH	2008	AA112a03R	N/A	JX448071
01_AE	TH	2008	AA113a_RH1	N/A	JX448086
01_AE	TH	2008	AA131a01R	N/A	JX448312
01_AE	TH	2009	R163b_p1	SG	JF680932
01_AE	TH	2009	AA045a03R	N/A	JX447235
01_AE	TH	2009	AA090a_WG1	N/A	JX447726
01_AE	TH	2009	AA111a_WG1	N/A	JX448057
01_AE	TH	2009	254001P00Ra.c01	SH	JX512892
01_AE	TH	2009	254003P00Ra.c01	MSM	JX512893
01_AE	TH	2009	254006P00Ra.c01	MSM	JX512894
01_AE	TH	2009	254007P00Ra.c01	MSM	JX512895
01_AE	TH	2009	254008P00Rd.c01	MSM	JX512896
01_AE	TH	2009	254010P00Ra.c01	MSM	JX512898
01_AE	TH	2009	SN40032_01_vA	SH	JX512901
01_AE	TH	2009	RTA2-env	N/A	KF268035
01_AE	TH	2009	RTA3-env	N/A	KF268036
01_AE	TH	2009	RTA4-env	N/A	KF268037
01_AE	TH	2009	RTA5-env	N/A	KF268038
01_AE	TH	2009	RTA6-env	N/A	KF268039
01_AE	TH	2009	RTA8-env	N/A	KF268040
01_AE	TH	2009	RTA9-env	N/A	KF268041
01_AE	TH	2009	RTA11-env	N/A	KF268042
01_AE	TH	2009	RTA13-env	N/A	KF268043
01_AE	TH	2009	RTA16-env	N/A	KF268044
01_AE	TH	2009	RTA21-env	N/A	KF268045
01_AE	TH	2009	RTA23-env	N/A	KF268046

01_AE	TH	2009	RTA24-env	N/A	KF268047
01_AE	TH	2009	RTA27-env	N/A	KF268048
01_AE	TH	2009	254002C01Ra	N/A	KT185744
01_AE	TH	2009	254004C01Ra	N/A	KT185983
01_AE	TH	2009	40061v03_06	N/A	KU230417
01_AE	TH	2009	254001_P00Ra	N/A	KU230427
01_AE	TH	2009	254006_P00Ra	N/A	KU230428
01_AE	TH	2009	254007_P00Ra	N/A	KU230429
01_AE	TH	2010	DE00110TH001	MSM	KP109513
01_AE	TH	2010	254016C01Ra	N/A	KT185678
01_AE	TH	2010	254024C01Ra	N/A	KT185724
01_AE	TH	2010	254034C01Ra	N/A	KT185793
01_AE	TH	2010	254032C01Ra	N/A	KT185921
01_AE	TH	2010	254019C01Rb	N/A	KT185941
01_AE	TH	2010	254023C01Ra	N/A	KT185962
01_AE	TH	2010	254029C01Ra	N/A	KT186005
01_AE	TH	2010	40094v01_01R	N/A	KU230418
01_AE	TH	2010	40100v01_06	N/A	KU230419
01_AE	TH	2010	40123v03_06R	N/A	KU230420
01_AE	TH	2010	40250v03_02R	N/A	KU230422
01_AE	TH	2010	254014_P00Rb	N/A	KU230430
01_AE	TH	2010	254018_P00a	N/A	KU230432
01_AE	TH	2010	254020_P00Ra	N/A	KU230433
01_AE	TH	2010	254021_P00c	N/A	KU230434
01_AE	TH	2010	254022_P00c	N/A	KU230435
01_AE	TH	2010	254026_P00Ra	N/A	KU230436
01_AE	TH	2010	254027_P00Ra	N/A	KU230437
01_AE	TH	2011	254036C01Ra	N/A	KT185851
01_AE	TH	2011	40231v02_02R	N/A	KU230421
01_AE	TH	2011	40363v03_01	N/A	KU230424
01_AE	TH	2011	40436v02_01	N/A	KU230426
01_AE	TH	-	NP03	N/A	AB485654
01_AE	TH	-	KSS_0514_13	N/A	HM215429
01_AE	TH	-	PSR_0508_2	N/A	HM215432
01_AE	TH	-	SPK_0525_13	N/A	HM215436
01_AE	TH	-	107747_048	N/A	JX848346
01_AE	TH	-	113035_007	N/A	JX848348
01_AE	TH	-	142902_011	N/A	JX848350
01_AE	TH	-	3007F3.A3	N/A	KJ952283
01_AE	TH	-	3017F5.A1	N/A	KJ952314
01_AE	TH	-	3098F5.A4	N/A	KJ952820
01_AE	TH	-	3126A02.B1	N/A	KJ952992
01_AE	TH	-	3127F6.A1	N/A	KJ953058
01_AE	TH	-	3139F5.A13	N/A	KJ953149
01_AE	TH	-	3141A16.B2	N/A	KJ953175
01_AE	TH	-	3155F6.A10	N/A	KJ953316
01_AE	TH	-	3156F6.A10	N/A	KJ953335
01_AE	TH	-	3202F6.B1	N/A	KJ953525
01_AE	TH	-	3220F5.A3	N/A	KJ953685
01_AE	VN	1997	97VNHCM301	N/A	FJ185237
01_AE	VN	1997	97VNHCM302	N/A	FJ185238
01_AE	VN	1997	97VNHCM303	N/A	FJ185239
01_AE	VN	1997	97VNHCM310	N/A	FJ185240
01_AE	VN	1997	97VNHCM314	N/A	FJ185241
01_AE	VN	1997	97VNHCM319	N/A	FJ185242
01_AE	VN	1997	97VNHCM343	N/A	FJ185243
01_AE	VN	1997	97VNHCM345	N/A	FJ185244
01_AE	VN	1997	97VNAG201	N/A	FJ185245
01_AE	VN	1997	97VNAG202	N/A	FJ185246
01_AE	VN	1997	97VNAG204	N/A	FJ185247
01_AE	VN	1997	97VNAG206	N/A	FJ185248
01_AE	VN	1997	97VNAG207	N/A	FJ185249
01_AE	VN	1997	97VNAG208	N/A	FJ185250
01_AE	VN	1997	97VNAG210	N/A	FJ185251
01_AE	VN	1997	97VNAG212	N/A	FJ185252
01_AE	VN	1997	97VNAG214	N/A	FJ185253

01_AE	VN	1997	97VNAG216	N/A	FJ185254
01_AE	VN	1997	97VNAG218	N/A	FJ185255
01_AE	VN	1997	97VNAG220	N/A	FJ185256
01_AE	VN	1997	97VNAG221	N/A	FJ185257
01_AE	VN	1997	97VNAG223	N/A	FJ185258
01_AE	VN	1997	97VNHCM306	N/A	FJ185259
01_AE	VN	1997	97VNHCM309	N/A	FJ185260
01_AE	VN	1998	98VNBG4	N/A	FJ185228
01_AE	VN	1998	98VNBG5	N/A	FJ185229
01_AE	VN	1998	98VNBG6	N/A	FJ185230
01_AE	VN	1998	98VNBG7	N/A	FJ185231
01_AE	VN	1998	98VNHD9	N/A	FJ185232
01_AE	VN	1998	98VNHD10	N/A	FJ185233
01_AE	VN	1998	98VNHD11	N/A	FJ185234
01_AE	VN	1998	98VNND15	N/A	FJ185235
01_AE	VN	1998	98VNND17	N/A	FJ185236
01B	CN	2006	CNHLJSM06059	N/A	EU131799
01B	CN	2007	07CNYN370	SU	KF835546
01B	CN	2010	JL.RF07	SG	KF859773
01B	CN	2011	JS2011001	SH	KM111555
01B	CN	2013	BJMP3037B	MSM	KP418805
01B	CN	2013	BJMP3194B	MSM	KP418806
01B	CN	2013	01B.CN.2012.11092	SH	KU501256
01B	CN	2014	12YN10135	SH	KT999999
01B	JP	2003	03JP-5091K279	MSM	AB859012
01B	JP	2005	05JPMYC113SP420	SH	LC027100
01B	JP	-	pHIV_1_Y271B01AE54	N/A	AB646289
01B	MM	1999	mCSW104	SW	AB097867
01B	MM	2000	mIDU502	PWID	AB097865
01B	MM	2013	mSSDU187	PWID	KU820835
01B	MY	2004	04MYKL019_1	N/A	DQ366665
01B	MY	2005	05MYKL043_1	N/A	DQ366666
01B	MY	2006	06MMYKLD46	PWID	EF495062
01B	MY	2007	07MYKLD48	N/A	EU031914
01B	MY	2008	08MYKL056	N/A	KT438782
01B	MY	2010	10MYPR226	PWID	KJ206289
01B	MY	2010	10MYKJ086	PWID	KT438783
01B	MY	2010	10MYPR70	PWID	KT438784
01B	TH	1991	CM237	SH	AY167123
01B	TH	1996	M005	SH	DQ354115
01B	TH	1996	M171	SH	DQ354121
01B	TH	1999	OUR044I	PWID	AY358042
01B	TH	1999	OUR2574	PWID	DQ354123
01B	TH	2000	00TH_C2254_BE	N/A	AY945733
01B	TH	2001	OUR033I	PWID	AY358071
01B	TH	2002	OUR847I	PWID	AY358069
01B	TH	2002	OUR840I	PWID	AY358070
01B	TH	2002	OUR846I	PWID	AY358072
01B	TH	2002	OUR740I	PWID	AY358073
01B	TH	2003	03TH700065	N/A	JN248316
01B	TH	2003	03TH700078	N/A	JN248317
01B	TH	2004	04TH228466	N/A	JN248319
01B	TH	2004	04TH312908	N/A	JN248320
01B	TH	2004	04TH321566	N/A	JN248322
01B	TH	2004	04TH322500	N/A	JN248323
01B	TH	2004	04TH423323	N/A	JN248326
01B	TH	2004	04TH704320	N/A	JN248331
01B	TH	2004	04TH801743	N/A	JN248332
01B	TH	2005	05TH245651	N/A	JN248340
01B	TH	2005	05TH443479	N/A	JN248349
01B	TH	2005	05TH518944	N/A	JN248350
01B	TH	2005	05TH637314	N/A	JN248352
01B	TH	2005	05TH852327	N/A	JN248357
01B	TH	2005	05TH140456	N/A	JN631793
01B	TH	2005	AA095a_WG16	N/A	JX447827
01B	TH	2005	AA106c05R	N/A	JX448016

01B	TH	2006	40CC3	N/A	EU743760
01B	TH	2006	40PB1	N/A	EU743761
01B	TH	2006	40PB3	N/A	EU743762
01B	TH	2006	AA020a_wg2	N/A	JX446927
01B	TH	2006	AA025a_WG1	N/A	JX446997
01B	TH	2006	AA084a_WG10	N/A	JX447668
01B	TH	2007	WN07	N/A	GQ916570
01B	TH	2007	MERLBDTRC3	N/A	JN860762
01B	TH	2007	MERLBDTRC6	N/A	JN860765
01B	TH	2007	AA096a_WG1	N/A	JX447833
01B	TH	-	NP1623	N/A	AF362994
01B	TH	-	TH283	N/A	AF468970
01B	TH	-	1269	N/A	AF490973
01B	TH	-	TH9_95	N/A	AF490974
01B	TH	-	TH_13-26	N/A	AY082968
01B	TH	-	MU2003	N/A	DQ354122
01B	TH	-	CU98_26	N/A	EF192591
01BC	CN	2007	07CNYN330	SU	KF835517
01BC	CN	2007	07CNYN334	SU	KF835520
01BC	CN	2007	07CNYN358	N/A	KF835537
01BC	CN	2008	BH095	N/A	KF803580
01BC	CN	2010	DH02	SH	KF250395
01BC	CN	2010	DH07	SH	KF250398
01BC	CN	2010	JL.RF01	SH	KF850149
01BC	CN	2010	JL.RF05	SH	KJ184177
01BC	CN	2011	ANHUI_BB17	MSM	KC183774
01BC	CN	2011	ANHUI_WH69	MSM	KC183781
01BC	CN	2012	DH28	SH	KF250407
01BC	CN	2013	S15-5h4-3h2-FL	MSM	KP170487
01BC	CN	2013	01BC.CN.2011.11312	SH	KU501257
01BC	CN	2013	SZ44LS7251	SH	KX378999
01BC	CN	2013	SZ95LS8027	SH	KX379000
01BC	CN	2014	BJ.2014.MSM.SP01	MSM	KP668994
01BC	CN	2014	XC2014EU01	SH	KX353919
01BC	CN	2015	ZJCIQ15005	N/A	KX010453
01BC	CN	2015	YA1996-00-NFLG	N/A	MF084205
01BC	MM	1999	mIDU107	PWID	AB097868
01BC	MM	2000	mCSW503	SW	AB097866
01BC	MM	2008	08mLDTD011	SH	KP455640
01BC	MM	2013	mSSDU139	PWID	KU820827
01BC	MM	2013	mSSDU153	PWID	KU820830
01BC	MM	2013	mSSDU199	PWID	KU820838
01BC	MM	2013	mSSDU247	PWID	KU820841
01BC	MM	2014	mKSDU24	PWID	KU820848
01C	CN	2008	08YN080	SH	HM138656
01C	CN	2010	DH18	SH	KF250401
01C	CN	2012	kang140-NFL	MSM	KJ778896
01C	CN	2013	kang019a-NFL	MSM	KJ778895
01C	CN	2014	12YN10159	SH	KT321211
01C	IN	1998	NARI-FLS_VB5	SH	KT175204
01C	IN	2014	NARI_FLS_YCM-151	SH	KT074935
01C	MM	2013	mSSDU101	PWID	KU820822
01C	NP	2011	11NP004	SH	KJ158422
01C	NP	2011	11NP016	SH	KJ158427
01C	NP	2011	11NP091	SH	KJ158437
01C	NP	2011	11NP115	MSM	KJ541850
01C	TH	-	NP1809	N/A	AY262830
01CD	NP	2011	11NP076	SH	KJ541842
02_AG	CN	2004	CNE85_U	N/A	HQ700002
02_AG	KR	2007	07MHI10	SH	JQ316136
02_AG	KR	2012	12MHR9	SW	KF561435
02_AG	PK	2014	PK005	PWID	KX232598
02_AG	PK	2015	PK024	PWID	KX232616
02_AG	PK	2015	PK032	PWID	KX232622
02_AG	PK	2015	PK035	PWID	KX232625
02_AG	TH	2007	AA039a11R	N/A	JX447146

02A1	PK	2014	DEURF14PK006	PWID	KU749413
02A1	PK	2014	DEURF14PK015	PWID	KU749414
02A1	PK	2014	PK003	PWID	KX232596
02A1	PK	2014	PK008	PWID	KX232601
02A1	PK	2014	PK012	PWID	KX232605
02A1	PK	2014	PK019	PWID	KX232612
02A1	PK	2015	PK023	PWID	KX232615
02A1	PK	2015	PK025	N/A	KX232617
02A1	PK	2015	PK033	PWID	KX232623
02A1	PK	2015	PK038	PWID	KX232627
02A1	PK	2015	PK039	PWID	KX232628
02A1	PK	2015	PK040	PWID	KX232629
07_BC	CN	1997	97CN001_C54	PWID	AF286226
07_BC	CN	1998	98CN009	N/A	AF286230
07_BC	CN	2003	BC14_BJ25_51	SU	EU363844
07_BC	CN	2004	CH064	PWID	EF117254
07_BC	CN	2004	CH070	PWID	EF117255
07_BC	CN	2004	CH091	PWID	EF117256
07_BC	CN	2004	CH110	PWID	EF117257
07_BC	CN	2004	CH111	PWID	EF117258
07_BC	CN	2004	CH181	PWID	EF117259
07_BC	CN	2004	CH120	PWID	EF117260
07_BC	CN	2004	CH119	PWID	EF117261
07_BC	CN	2004	CH117	PWID	EF117262
07_BC	CN	2004	CH115	PWID	EF117263
07_BC	CN	2004	CH114	PWID	EF117264
07_BC	CN	2004	CBJ008	PWID	JF346900
07_BC	CN	2004	CBJA017	PWID	JF346901
07_BC	CN	2004	CBJA021	PWID	JF346902
07_BC	CN	2004	CBJA022	PWID	JF346903
07_BC	CN	2004	CBJA023	PWID	JF346904
07_BC	CN	2004	CBJA025	PWID	JF346905
07_BC	CN	2004	CBJA028	PWID	JF346906
07_BC	CN	2005	XJDC6441	SH	EF368370
07_BC	CN	2005	XJN0084	PWID	EF368371
07_BC	CN	2005	XJDC6431_2	PWID	EF368372
07_BC	CN	2005	pXJDC6441-2	PWID	EF420986
07_BC	CN	2005	BC07_YN109_6	PWID	EU363837
07_BC	CN	2005	BC10_YN55_3	PWID	EU363840
07_BC	CN	2005	BC18_XJ153_35	PWID	EU363848
07_BC	CN	2005	pXJDC6291-13	PWID	KC492737
07_BC	CN	2006	CNHLJ_M06054	N/A	EU131811
07_BC	CN	2006	CNE92_U	N/A	HQ699994
07_BC	CN	2006	CBJB347	PWID	JF346915
07_BC	CN	2006	Sichuan_2006_SC006	PWID	JX392378
07_BC	CN	2006	Sichuan_2006_SC020	PWID	JX392380
07_BC	CN	2006	Sichuan_2006_SC025	PWID	JX392381
07_BC	CN	2006	Sichuan_2006_SC124	PWID	JX392382
07_BC	CN	2006	Xinjiang_2006_709	PWID	JX392383
07_BC	CN	2006	Xinjiang_2006_713	PWID	JX392384
07_BC	CN	2006	SC070059	PWID	KF250376
07_BC	CN	2007	BJOX002000_01_3	PWID	HM215363
07_BC	CN	2007	BJOX011000_01_3	MSM	HM215375
07_BC	CN	2007	BJOX019000_02_1	MSM	HM215380
07_BC	CN	2007	BJOX027000_02_1	MSM	HM215388
07_BC	CN	2007	BJOX029000_03_1	MSM	HM215392
07_BC	CN	2007	CNE19	SH	HM215405
07_BC	CN	2007	CNE20	SH	HM215406
07_BC	CN	2007	CNE21	SH	HM215407
07_BC	CN	2007	CNE39	N/A	HQ699965
07_BC	CN	2007	CNE68	SH	HQ699982
07_BC	CN	2007	CNE69	N/A	HQ699983
07_BC	CN	2007	CNE70	N/A	HQ699984
07_BC	CN	2007	CNGZD	PWID	JQ423923
07_BC	CN	2007	BJ070032	MSM	KF250366
07_BC	CN	2007	GD070145	PWID	KF250371

07_BC	CN	2007	GZ070087	MSM	KF250372
07_BC	CN	2007	HB070037	SH	KF250373
07_BC	CN	2007	JS070205	SH	KF250374
07_BC	CN	2007	NX070014	PWID	KF250375
07_BC	CN	2007	XJ070248	PWID	KF250377
07_BC	CN	2007	xj070241	SH	KF250385
07_BC	CN	2007	07CNYN303	PWID	KF835494
07_BC	CN	2007	07CNYN323	PWID	KF835510
07_BC	CN	2007	07CNYN324	PWID	KF835511
07_BC	CN	2007	07CNYN349	PWID	KF835532
07_BC	CN	2007	BJOX016000.e02	MSM	KM217857
07_BC	CN	2008	1114	MSM	HQ215552
07_BC	CN	2008	08LNA001	MSM	JX960601
07_BC	CN	2008	BJOX037000.e01	MSM	KM218157
07_BC	CN	2009	CBJA552	PWID	JF346907
07_BC	CN	2009	CBJA555	PWID	JF346908
07_BC	CN	2009	CBJA556	PWID	JF346909
07_BC	CN	2009	CBJA557	PWID	JF346910
07_BC	CN	2009	CBJA558	PWID	JF346911
07_BC	CN	2009	CBJA561	PWID	JF346912
07_BC	CN	2009	CBJA563	PWID	JF346913
07_BC	CN	2009	CBJA570	PWID	JF346914
07_BC	CN	2009	09LNA446	MSM	JX960600
07_BC	CN	2009	09LNA745	MSM	JX960602
07_BC	CN	-	CNGL179	N/A	AF503396
07_BC	CN	-	BC02_XJ74_2	PWID	EU363832
07_BC	CN	-	BC12_SC142_6	PWID	EU363842
07_BC	CN	-	BC16_BJ29_34	SU	EU363846
07_BC	CN	-	HB5_3	N/A	HQ326124
07_BC	CN	-	BJ22_5	N/A	HQ326125
07_BC	CN	-	BJ24_3	N/A	HQ326126
07_BC	CN	-	SC11_59	N/A	HQ326127
07_BC	CN	-	SC19_15	N/A	HQ326129
07_BC	CN	-	SC20_15	N/A	HQ326130
07_BC	CN	-	SC21_28	N/A	HQ326131
07_BC	CN	-	SC22_16	N/A	HQ326132
07_BC	CN	-	XJ16_6	N/A	HQ326133
07_BC	CN	-	XJ47_5	N/A	HQ326134
07_BC	CN	-	XJ50_6	N/A	HQ326135
07_BC	CN	-	XJ180_29	N/A	HQ326136
07_BC	CN	-	YN99r_5	N/A	HQ326137
07_BC	CN	-	YN108r_4	N/A	HQ326138
07_BC	CN	-	YN148r_9	N/A	HQ326139
07_BC	CN	-	GX33m_25	N/A	HQ326141
07_BC	CN	-	GX45_57	N/A	HQ326143
07_BC	CN	-	GX84_59	N/A	HQ326146
07_BC	MM	2013	mSSDU163	PWID	KU820832
07_BC	TW	2004	TW_D3	PWID	DQ230841
07_BC	TW	2013	pCRF07	PWID	KF234628
07B	TW	2004	TW_D60	PWID	DQ230842
08_BC	CN	1997	97CNGX_6F	PWID	AY008715
08_BC	CN	1997	97CNGX_7F	PWID	AY008716
08_BC	CN	1997	97CNGX_9F	PWID	AY008717
08_BC	CN	1998	98CN006	N/A	AF286229
08_BC	CN	2000	p00CH-WS035_08_BC51	PWID	AB746344
08_BC	CN	2000	p00CH-HH090_08_BC02	PWID	AB773884
08_BC	CN	2000	QJ001	N/A	KC914396
08_BC	CN	2001	p01CH-DL001_08_BC06	N/A	AB746342
08_BC	CN	2005	BC06_YNP45_45	PWID	EU363836
08_BC	CN	2006	BC01_YN161_5	PWID	EU363831
08_BC	CN	2006	BC17_YN78_8	PWID	EU363847
08_BC	CN	2006	nx2	N/A	HM067748
08_BC	CN	2007	CNE52	PWID	HM215416
08_BC	CN	2007	CNE53	PWID	HM215417
08_BC	CN	2007	CNE22	N/A	HQ699952
08_BC	CN	2007	CNE24	N/A	HQ699953

08_BC	CN	2007	CNE25	N/A	HQ699954
08_BC	CN	2007	CNE29	N/A	HQ699957
08_BC	CN	2007	CNE32	N/A	HQ699958
08_BC	CN	2007	CNE33	N/A	HQ699959
08_BC	CN	2007	CNE35	N/A	HQ699961
08_BC	CN	2007	CNE36	N/A	HQ699962
08_BC	CN	2007	CNE37	N/A	HQ699963
08_BC	CN	2007	CNE38	N/A	HQ699964
08_BC	CN	2007	CNE41	N/A	HQ699966
08_BC	CN	2007	CNE42	N/A	HQ699967
08_BC	CN	2007	CNE43	N/A	HQ699968
08_BC	CN	2007	CNE44	N/A	HQ699969
08_BC	CN	2007	CNE45	N/A	HQ699970
08_BC	CN	2007	CNE46	PWID	HQ699971
08_BC	CN	2007	CNE47	PWID	HQ699972
08_BC	CN	2007	CNE48	N/A	HQ699973
08_BC	CN	2007	CNE49	PWID	HQ699974
08_BC	CN	2007	CNE50	N/A	HQ699975
08_BC	CN	2007	CNE54	N/A	HQ699976
08_BC	CN	2007	CNE80_U	N/A	HQ700001
08_BC	CN	2007	CNE51	N/A	HQ700004
08_BC	CN	2007	2007CNGX_HK	PWID	JF719819
08_BC	CN	2007	GD070060	PWID	KF250368
08_BC	CN	2007	GD070077	PWID	KF250369
08_BC	CN	2007	07CNYN302	PWID	KF835493
08_BC	CN	2007	07CNYN306	PWID	KF835495
08_BC	CN	2007	07CNYN307	SU	KF835496
08_BC	CN	2007	07CNYN310	SU	KF835497
08_BC	CN	2007	07CNYN313	MB	KF835500
08_BC	CN	2007	07CNYN314	PWID	KF835501
08_BC	CN	2007	07CNYN319	SU	KF835506
08_BC	CN	2007	07CNYN320	PWID	KF835507
08_BC	CN	2007	07CNYN321	PWID	KF835508
08_BC	CN	2007	07CNYN322	SU	KF835509
08_BC	CN	2007	07CNYN325	SU	KF835512
08_BC	CN	2007	07CNYN344	PWID	KF835529
08_BC	CN	2007	07CNYN346	PWID	KF835530
08_BC	CN	2007	07CNYN350	PWID	KF835531
08_BC	CN	2007	07CNYN355	SU	KF835534
08_BC	CN	2007	07CNYN351	SU	KF835535
08_BC	CN	2007	07CNYN363	PWID	KF835541
08_BC	CN	2007	07CNYN369	PWID	KF835545
08_BC	CN	2007	07CNYN371	PWID	KF835547
08_BC	CN	-	YN177_1	N/A	HQ326140
08_BC	CN	-	GX43_2	N/A	HQ326142
08_BC	CN	-	GX75_20	N/A	HQ326144
08_BC	MM	2013	mSSDU220	PWID	KU820840
08_BC	MM	2013	mSSDU91	PWID	KU820846
15_01B	TH	1996	M169	SH	DQ354120
15_01B	TH	1999	99TH_MU2079	N/A	AF516184
15_01B	TH	1999	99TH_R2399	N/A	AF530576
15_01B	TH	2002	02TH_OUR1331	N/A	AF529572
15_01B	TH	2002	02TH_OUR1332	N/A	AF529573
15_01B	TH	2004	04TH409819	N/A	JN248325
15_01B	TH	2005	05TH522586	N/A	JN248351
15_01B	TH	-	3147F5.D15	N/A	KJ953197
16_A2D	KR	1997	97KR004	SW	AF286239
33_01B	ID	2007	JKT189-C	N/A	AB547463
33_01B	ID	2007	JKT194-C	SH	AB547464
33_01B	MY	2005	05MYKL007_1	SG	DQ366659
33_01B	MY	2005	05MYKL015_2	SG	DQ366660
33_01B	MY	2005	05MYKL031_1	SH	DQ366661
33_01B	MY	2005	05MYKL045_1	SH	DQ366662
33_01B	MY	2007	07MYKLD47	N/A	EU031913
34_01B	TH	1999	OUR1969P	PWID	EF165539
34_01B	TH	1999	OUR2275P	PWID	EF165540

34_01B	TH	1999	OUR2478P	PWID	EF165541
48_01B	MY	2007	07MYKT014	PWID	GQ175881
48_01B	MY	2007	07MYKT016	PWID	GQ175882
48_01B	MY	2007	07MYKT021	PWID	GQ175883
51_01B	MY	2009	09MYKL050	N/A	KJ485698
51_01B	MY	2011	11MYKL055	N/A	KJ485697
51_01B	SG	2011	11SG_HM021	SG	JN029801
51_01B	SG	2011	11SG_HM091	SG	JN029803
52_01B	MY	2003	03MYKL018_1	SB	DQ366664
52_01B	TH	1996	M043	SH	DQ354113
52_01B	TH	2000	00TH_R1741	N/A	AY945734
53_01B	MY	2004	04MYKL016_1	SH	DQ366663
53_01B	MY	2010	10MYKJ079	PWID	JX390611
53_01B	MY	2010	10MYKJ067	PWID	JX390612
53_01B	MY	2011	11FIR164	N/A	JX390610
54_01B	MY	2007	07MYKLD49	N/A	EU031915
54_01B	MY	2008	08MYKL044	N/A	JX390977
54_01B	MY	2009	09MYSB023	N/A	JX390976
55_01B	CN	2008	08CYM047	MSM	JF340054
55_01B	CN	2010	HNCS102056	MSM	JX574661
55_01B	CN	2011	GDDG318	MSM	JX574662
55_01B	CN	2011	GDDG095	MSM	JX574663
55_01B	CN	2011	ANHUI_FY64	MSM	KC183777
55_01B	CN	2011	11HNCS500434	MSM	KF927150
55_01B	CN	2011	11CN.GDDG325	MSM	KF927151
57_BC	CN	2007	341	PWID	HM776939
57_BC	CN	2009	1439	PWID	JX679207
57_BC	CN	2009	YNFL37	SH	KC870044
57_BC	CN	2009	09YNLX19sg	PWID	KC899008
57_BC	CN	2010	DH17	SH	KF250400
58_01B	MY	2009	09MYPR37	PWID	KC522031
58_01B	MY	2010	10MYKJ036	PWID	KC522035
58_01B	MY	2010	10MYPR87	PWID	KF425293
58_01B	MY	2011	11MY1ZK731	PWID	KC522032
58_01B	MY	2011	11MY1RJ704	PWID	KC522033
58_01B	MY	2011	11MY1EP794	PWID	KC522034
59_01B	CN	2007	GD070126	SG	KF011494
59_01B	CN	2007	GX070051	SG	KF011495
59_01B	CN	2009	09LNA423	MSM	JX960635
59_01B	CN	2010	10LNSY300533	MSM	KC462191
59_01B	CN	2011	11LNSY300392	MSM	KC462190
59_01B	CN	2011	11CN.GDMM152	MSM	KJ484433
59_01B	CN	2011	11CN.LNSY300876	SH	KJ484434
59_01B	CN	2012	12CN.YNKM200199	MSM	KJ484435
61_BC	CN	2007	FJ070004	PB	KC990125
61_BC	CN	2007	JL070009	SH	KC990126
61_BC	CN	2010	JL100010	SH	KC990124
62_BC	CN	2010	YNFL13	PWID	KC870034
62_BC	CN	2010	YNFL15	SH	KC870035
62_BC	CN	2010	YNFL18	SH	KC870037
64_BC	CN	2009	YNFL08	PWID	KC870031
64_BC	CN	2009	YNFL31	SH	KC870042
64_BC	CN	2009	YNFL33	PWID	KC870043
64_BC	CN	2009	09YNLX047sg	PWID	KC898994
64_BC	CN	2009	09YNLX219037sg	PWID	KC899009
64_BC	CN	2010	YNFL10_1	PWID	KC870032
64_BC	CN	2010	YNFL16	SH	KC870036
64_BC	CN	2010	YNFL22	SH	KC870040
65_cpx	CN	2009	YNFL05	SH	KC870030
65_cpx	CN	2010	YNFL01	SH	KC870027
65_cpx	CN	2010	YNFL02	SH	KC870028
65_cpx	CN	2011	ANHUI_HF104	MSM	KC183778
67_01B	CN	2011	ANHUI_HF115	MSM	KC183779
67_01B	CN	2011	ANHUI_MAS59	MSM	KC183780
68_01B	CN	2010	JS2010001	MSM	KF758551
68_01B	CN	2011	ANHUI_WH73	MSM	KC183782

68_01B	CN	2011	ANHUI_XC46	MSM	KC183783
69_01B	JP	2003	03JP-5091K231	MSM	AB845344
69_01B	JP	2004	04JP-5091K320	MSM	AB845345
69_01B	JP	2004	04JP-5091K323	MSM	AB845346
69_01B	JP	2005	05JP-5091K448	MSM	AB845347
69_01B	JP	2010	10JP-5091N172	SB	AB845348
69_01B	JP	2010	10JP-5091N200	SH	AB845349
74_01B	MY	2010	10MYKJ052	PWID	KR019770
74_01B	MY	2010	10MYPR268	PWID	KR019771
74_01B	MY	2011	11MYPR416	PWID	KR019772
77_cpx	MY	2013	13MYNBB108	N/A	KX673818
77_cpx	MY	2014	14MYNBB084	N/A	KX673819
77_cpx	MY	2014	14MYNBB090	N/A	KX673820
77_cpx	MY	2014	14MYNBB164	N/A	KX673821
78_cpx	CN	2013	YNTC19	SH	KU161143
78_cpx	CN	2013	YNTC35	SH	KU161144
78_cpx	CN	2013	YNTC88	PWID	KU161145
82_cpx	MM	2013	mSSDU12	PWID	KU820825
82_cpx	MM	2013	mSSDU160	PWID	KU820831
82_cpx	MM	2013	mSSDU191	PWID	KU820836
82_cpx	MM	2013	mSSDU195	PWID	KU820837
82_cpx	MM	2013	mSSDU63	PWID	KU820844
82_cpx	MM	2013	mSSDU75	PWID	KU820845
83_cpx	MM	2013	mSSDU109	PWID	KU820823
83_cpx	MM	2013	mSSDU118	PWID	KU820824
83_cpx	MM	2013	mSSDU137	PWID	KU820826
83_cpx	MM	2013	mSSDU144	PWID	KU820828
83_cpx	MM	2013	mSSDU151	PWID	KU820829
83_cpx	MM	2013	mSSDU178	PWID	KU820833
83_cpx	MM	2013	mSSDU180	PWID	KU820834
83_cpx	MM	2013	mSSDU21	PWID	KU820839
83_cpx	MM	2013	mSSDU24	PWID	KU820842
83_cpx	MM	2013	mSSDU28	PWID	KU820843
83_cpx	MM	2013	mSSDU94	PWID	KU820847
85_BC	CN	2014	14CN_SCYB1	SH	KU992928
85_BC	CN	2014	14CN_SCYB2	SH	KU992929
85_BC	CN	2014	14CN_SCYB20	SH	KU992930
85_BC	CN	2014	14CN_SCYB3	SH	KU992931
85_BC	CN	2014	14CN_SCYB4	SH	KU992932
85_BC	CN	2014	14CN_SCYB7	SH	KU992934
85_BC	CN	2014	14CN_SCYB11	SH	KU992935
85_BC	CN	2014	14CN_SCYB12	SH	KU992936
85_BC	CN	2014	14CN_SCYB18	SH	KU992937
86_BC	CN	2013	15YNHS18	SH	KX582249
86_BC	CN	2013	15YNHS23	SH	KX582250
86_BC	CN	2013	15YNHS26	SH	KX582251
87_cpx	CN	2009	09YNLC497sg	PWID	KC898992
87_cpx	CN	2009	09YNRL215050sg	PWID	KC899012
87_cpx	CN	2012	DH32	SH	KF250408
88_BC	CN	2005	05YNRL07sg	PWID	KC898975
88_BC	CN	2005	05YNRL25sg	PWID	KC898979
88_BC	CN	2009	DH19	PWID	KF250402
A1	IN	1997	NARI-FLS_VB6	SH	KT152840
A1	IN	1999	NARI-FLS_VB11	SH	KT152841
A1	IN	1999	NARI-FLS_VB15	SH	KT152842
A1	IN	1999	NARI-FLS_VB81-4	SH	KT152843
A1	IN	2000	NARI-FLS_VB99-30	MB	KT152846
A1	IN	2009	NARI-FLS_IVC19-1	SH	KT152839
A1	NP	2011	11NP086	SH	KJ158436
A1	PK	2014	DEMA114PK001	SH	KU749409
A1	PK	2014	DEMA114PK002	SH	KU749410
A1	PK	2014	DEMA114PK013	PWID	KU749411
A1	PK	2014	PK004	PWID	KX232597
A1	PK	2014	PK007	PWID	KX232600
A1	PK	2014	PK014	PWID	KX232607
A1	PK	2014	PK016	PWID	KX232609

A1	PK	2014	PK017	PWID	KX232610
A1	PK	2014	PK018	PWID	KX232611
A1	PK	2014	PK020	PWID	KX232613
A1	PK	2014	PK027	PWID	KX232619
A1	PK	2015	PK021	PWID	KX232614
A1	PK	2015	PK026	PWID	KX232618
A1	PK	2015	PK030	PWID	KX232620
A1	PK	2015	PK031	PWID	KX232621
A1	PK	2015	PK034	PWID	KX232624
A1	PK	2015	PK036	PWID	KX232626
A1C	IN	1995	95IN21301	SH	AF067156
A1C	IN	1999	NARI-FLS_VB27-5	SH	KT175205
A1C	IN	2001	1579A	N/A	DQ083238
A1C	IN	2006	NARI-FLS_IVC3-1.28	SH	KT175202
A1C	IN	2009	NARI-FLS_09-387	SH	KC911635
A1C	NP	2011	11NP122	MSM	KJ541851
B	CN	1998	YN9802	PWID	JF932495
B	CN	1998	YN9838	PWID	JF932496
B	CN	1999	plwj	PB	GU177863
B	CN	2001	CNHN24	PB	AY180905
B	CN	2002	02HNsmx2	N/A	DQ007901
B	CN	2002	02HNsq4	N/A	DQ007902
B	CN	2002	02HNsc11	N/A	DQ007903
B	CN	2003	CHNHLJBSF03009c34	N/A	AY905493
B	CN	2003	CNHLJBM03010	N/A	EU131793
B	CN	2003	B01	NO	EU363825
B	CN	2003	B02	NO	EU363826
B	CN	2003	B03	SU	EU363827
B	CN	2003	SHXDC0081	SH	JF932492
B	CN	2004	CHNHLJSM04028c3	N/A	AY905494
B	CN	2004	CHNHLJBF04029c2	N/A	AY905495
B	CN	2004	CHNHLJSM0417c3	N/A	AY905496
B	CN	2004	CHNHLJBF04016c4	N/A	AY905497
B	CN	2004	CNHLJBM04038	N/A	EU131787
B	CN	2004	CNHLJBF04026	N/A	EU131788
B	CN	2004	CNHLJBM04020	N/A	EU131789
B	CN	2004	CNHLJBF04025	N/A	EU131790
B	CN	2004	CNHLJBF04024	N/A	EU131791
B	CN	2004	CNHLJBF04019	N/A	EU131792
B	CN	2004	CNHLJBM04018	N/A	EU131796
B	CN	2004	CNHLJBF04023	N/A	EU131797
B	CN	2004	CNHLJBF04021	N/A	EU131801
B	CN	2004	CNHLJBF04032	N/A	EU131806
B	CN	2004	CNHLJBF04030	N/A	EU131808
B	CN	2004	CNE10	PB	HM215397
B	CN	2004	CNE12	SH	HM215399
B	CN	2004	CNE14	SH	HM215400
B	CN	2004	CNE13	N/A	HQ699951
B	CN	2004	CNE63	N/A	HQ699978
B	CN	2004	CNE64	PB	HQ699979
B	CN	2004	CNE82_U	N/A	HQ699989
B	CN	2004	CNE83_U	N/A	HQ699990
B	CN	2004	CNE99_U	N/A	HQ699996
B	CN	2004	CNE100_U	N/A	HQ699997
B	CN	2005	05CNHB_hp3	MB	DQ990880
B	CN	2005	CNHLJBM05036	N/A	EU131803
B	CN	2005	CNE11	PB	HM215398
B	CN	2005	CNE57	MSM	HM215420
B	CN	2005	CNE9	SH	HM215428
B	CN	2005	CNE95_U	N/A	HQ699995
B	CN	2005	CNE101_U	N/A	HQ699998
B	CN	2006	CNHLJSM06048	N/A	EU131794
B	CN	2006	CNHLJBM06050	N/A	EU131798
B	CN	2006	CNHLJBM06057	N/A	EU131800
B	CN	2006	CNHLJBF06051	N/A	EU131802
B	CN	2006	CNHLJSF06056	N/A	EU131804

B	CN	2006	CNHLJBF06044	N/A	EU131805
B	CN	2006	CNHLJ_M06053	N/A	EU131807
B	CN	2006	CNHLJ_M06058	N/A	EU131809
B	CN	2006	CNHLJSF06060	N/A	EU131810
B	CN	2006	B04	NO	EU363828
B	CN	2006	B05	NO	EU363829
B	CN	2006	CNE4	PWID	HM215413
B	CN	2006	CNE6	PWID	HM215423
B	CN	2006	CNE1	PWID	HQ699949
B	CN	2006	CC056	N/A	JF932482
B	CN	2007	BJOX003000_19_1	MSM	HM215365
B	CN	2007	BJOX006000_13_3	MSM	HM215369
B	CN	2007	BJOX007000_02_3	MSM	HM215370
B	CN	2007	BJOX020000_03_3	MSM	HM215382
B	CN	2007	BJOX022000_02_3	MSM	HM215384
B	CN	2007	CNE105_U	N/A	HQ700000
B	CN	2007	AH070011	N/A	JF932468
B	CN	2007	AH070014	N/A	JF932469
B	CN	2007	AH070017	N/A	JF932470
B	CN	2007	AH070018	N/A	JF932471
B	CN	2007	AH070057	N/A	JF932472
B	CN	2007	BJ070030	PWID	JF932473
B	CN	2007	CBJC261	N/A	JF932474
B	CN	2007	CBJC392	N/A	JF932475
B	CN	2007	CBJC394	N/A	JF932476
B	CN	2007	CBJC396	N/A	JF932477
B	CN	2007	FJ070016	SH	JF932483
B	CN	2007	GS070017	NO	JF932484
B	CN	2007	GZ070002	N/A	JF932485
B	CN	2007	GZ070030	N/A	JF932486
B	CN	2007	HB070006	N/A	JF932487
B	CN	2007	HB070022	N/A	JF932488
B	CN	2007	HB070035	N/A	JF932489
B	CN	2007	JL070038	N/A	JF932490
B	CN	2007	JS070389	SH	JF932491
B	CN	2007	SX070080	SH	JF932493
B	CN	2007	hb070025	N/A	JF932499
B	CN	2007	hen1345	N/A	JF932500
B	CN	2007	BJOX004000.e03	MSM	KM217617
B	CN	2007	BJOX014000.e01	MSM	KM217780
B	CN	2008	1106	MSM	HQ215554
B	CN	2008	CBJC502	PB	JF346917
B	CN	2008	CBJC507	PB	JF346918
B	CN	2008	CBJC513	PB	JF346919
B	CN	2008	CBJC476	N/A	JF932478
B	CN	2008	CBJC489	N/A	JF932479
B	CN	2008	CBJC500	N/A	JF932480
B	CN	2008	CBJC503	N/A	JF932481
B	CN	2008	cbjc468	N/A	JF932498
B	CN	2008	BJOX035000.e01	MSM	KM218138
B	CN	2008	BJOX041000.e01	MSM	KM218159
B	CN	2008	BJOX046000.e01	MSM	KM218184
B	CN	2008	BJOX047000.e01	MSM	KM218210
B	CN	2009	1121	MSM	HQ215556
B	CN	2009	CBJC485	PB	JF346916
B	CN	2009	YN09P0014	PWID	JF932494
B	CN	2009	ZK042	N/A	JF932497
B	CN	2009	09LNA014	MSM	JX960597
B	CN	2009	09LNA439	MSM	JX960598
B	CN	2009	09LNA336	MSM	JX960599
B	CN	2009	DEMB09CN002	SH	KC596066
B	CN	2009	09YNRL215042sg	PWID	KC899011
B	CN	2009	DEMB09CN013	MSM	KP109510
B	CN	2010	DEMB10CN002	N/A	JX140658
B	CN	2012	DEMB12CN006	MSM	KP109511
B	CN	2012	DEMB12CN010	SH	KP109512

B	CN	2012	134-0-10	N/A	KX692903
B	CN	2012	15-0-10	N/A	KX692925
B	CN	2012	2019-0-2	N/A	KX692941
B	CN	2012	2039-0-1	N/A	KX692948
B	CN	2012	2079-1	N/A	KX692969
B	CN	2012	2081-1	N/A	KX692980
B	CN	2012	2092-0-1	N/A	KX692997
B	CN	2012	2097-0-10	N/A	KX693025
B	CN	2012	2110-10	N/A	KX693036
B	CN	2012	2124-0-1	N/A	KX693054
B	CN	2012	2142-0-1	N/A	KX693065
B	CN	2012	2157-1	N/A	KX693075
B	CN	2012	2162-11	N/A	KX693092
B	CN	2012	2243-0-3	N/A	KX693099
B	CN	2012	2259-1	N/A	KX693100
B	CN	2012	2350-0-1	N/A	KX693113
B	CN	2012	2357-11	N/A	KX693131
B	CN	2012	2358-0-1	N/A	KX693150
B	CN	2012	2360-0-1a	N/A	KX693166
B	CN	2012	2361-0-1	N/A	KX693171
B	CN	2012	2362-0-1	N/A	KX693190
B	CN	2012	2363-1	N/A	KX693215
B	CN	2012	2406-12	N/A	KX693238
B	CN	2012	2408-0-1	N/A	KX693247
B	CN	2012	2418-0-14	N/A	KX693265
B	CN	2012	2460-11	N/A	KX693281
B	CN	2012	3124-0-17	N/A	KX693301
B	CN	2012	3559-11	N/A	KX693308
B	CN	2012	3560-1	N/A	KX693314
B	CN	2012	3581-1	N/A	KX693322
B	CN	2012	3596-1	N/A	KX693338
B	CN	2012	3617-1	N/A	KX693357
B	CN	2012	3624-10	N/A	KX693366
B	CN	2012	3627-1	N/A	KX693387
B	CN	2012	3786-012C	N/A	KX693393
B	CN	2012	3796-1	N/A	KX693415
B	CN	2012	3832-1	N/A	KX693430
B	CN	2012	3841-10	N/A	KX693454
B	CN	2012	3856-1	N/A	KX693468
B	CN	2012	3858-11	N/A	KX693475
B	CN	2012	3879-2	N/A	KX693489
B	CN	2012	3899-2	N/A	KX693491
B	CN	2012	3954-0-1	N/A	KX693496
B	CN	2012	543-0-1	N/A	KX693515
B	CN	2012	555-0-1	N/A	KX693533
B	CN	-	B06	N/A	EU363830
B	CN	-	RL42	PWID	U71182
B	HK	2006	HK002	N/A	FJ460499
B	HK	2006	HK003	N/A	FJ460500
B	HK	2006	HK004	N/A	FJ460501
B	IN	2007	NARI-L5_NEM.J3	N/A	FJ515874
B	IN	-	11807	N/A	EF694037
B	JP	1988	2	NO	AB588196
B	JP	1988	3	NO	AB588207
B	JP	1989	31	MSM	AB588209
B	JP	1989	33	SH	AB588211
B	JP	1989	40	NO	AB588223
B	JP	1989	60	MSM	AB588232
B	JP	1998	DR1120	N/A	AB480698
B	JP	1999	DR1348	N/A	AB287369
B	JP	2000	DR2508	N/A	AB289587
B	JP	2000	117	N/A	AB428551
B	JP	2001	DR388	N/A	AB289589
B	JP	2001	134	N/A	AB428552
B	JP	2002	194	N/A	AB428553
B	JP	2002	197	N/A	AB428554

B	JP	2003	227	N/A	AB428555
B	JP	2003	285	N/A	AB428558
B	JP	2004	04JPDR6075B	N/A	AB221125
B	JP	2004	DR6089	N/A	AB286955
B	JP	2004	329	N/A	AB428559
B	JP	2004	DR6174	N/A	AB480692
B	JP	2004	DR6175	N/A	AB480694
B	JP	2004	DR5913	N/A	AB480696
B	JP	2005	DR6538	N/A	AB287363
B	JP	2005	DR6737	N/A	AB287364
B	JP	2005	DR7060	MSM	AB287366
B	JP	2005	DR7065	N/A	AB287368
B	JP	2005	426	N/A	AB428556
B	JP	2005	436	N/A	AB428557
B	JP	2005	398	N/A	AB428560
B	JP	2005	DR6657	MSM	AB588243
B	JP	2005	DR6739	SH	AB588253
B	JP	2005	DR6826	MSM	AB588263
B	JP	2005	DR6871	MSM	AB588271
B	JP	2005	DR6946	SH	AB588283
B	JP	2005	DR7015	SH	AB588291
B	JP	2006	472	N/A	AB428561
B	JP	2006	574	N/A	AB428562
B	JP	2006	DR7259	SH	AB588311
B	JP	2006	DR7374	MSM	AB588329
B	JP	2008	NMC104_clone_01	N/A	AB731663
B	JP	2009	NMC851A_clone_14	N/A	AB731665
B	JP	2009	NMC127_clone_07	N/A	AB731667
B	JP	2011	DEMB11JP002	N/A	KF716497
B	JP	2012	DEMB12JP001	N/A	KF716498
B	JP	-	MOKW9C	SH	AB262952
B	JP	-	DR1673	N/A	AB564744
B	JP	-	DR2735	N/A	AB564746
B	JP	-	pJPDR0796B02	N/A	AB565478
B	JP	-	JRC03B	N/A	AB565495
B	JP	-	JRC05B	N/A	AB565497
B	JP	-	JRC53B	N/A	AB565499
B	JP	-	JRC65B	N/A	AB565501
B	JP	-	DR1712	N/A	AB604946
B	JP	-	DR1777	N/A	AB604948
B	JP	-	DR5929	N/A	AB604950
B	JP	-	pJRC57B09	N/A	AB641836
B	JP	-	KP_5_48_A02	N/A	AB742145
B	JP	-	ETR	EX	D12582
B	JP	-	KKwt_12	N/A	KT961002
B	KR	1991	91LCS9-15330_15319	N/A	KX960965
B	KR	1991	91KYB12-15320_15293_15314	N/A	KX960966
B	KR	1991	91LGJ11-15285_15339_15331	N/A	KX960967
B	KR	1991	91PJB12-15284_15329_15280	N/A	KX960973
B	KR	1992	KR2057_C1	SG	AJ417423
B	KR	1992	HP-1_92LYS9-4500	PH	KJ140245
B	KR	1992	HP-2_97JJW2-12101	PH	KJ140247
B	KR	1992	HP-3_02LJW8-4757	PH	KJ140248
B	KR	1992	HP-6_03JHJ2-3477	PH	KJ140251
B	KR	1992	HP-7_96LSM10-3474	PH	KJ140252
B	KR	1992	HP-10_02SHJ8-6986	PH	KJ140255
B	KR	1992	HP-11_02PGU10-4780	PH	KJ140256
B	KR	1992	HP-12_02LGH10-3448	PH	KJ140257
B	KR	1992	HP-14_97KTG8-3466	PH	KJ140259
B	KR	1992	HP-16_12JIS11-6075	PH	KJ140261
B	KR	1992	93LSW7-10899	PH	KJ140266
B	KR	1992	94KHB5-3295	N/A	KJ140267
B	KR	1992	92KYJ5-13316	SH	KU869580
B	KR	1992	92YWS6-13327_13320	SG	KU869584
B	KR	1992	92HSH6-13313	SG	KU869589
B	KR	1992	92PJA12-13306	SG	KU869595

B	KR	1992	92DGi6-13344	N/A	KU869604
B	KR	1992	92PTH3-13342	N/A	KU869607
B	KR	1992	92KSS12-13399	SG	KU896115
B	KR	1992	92JSH3-13355	N/A	KU896116
B	KR	1992	92CYK6-13395	SG	KU896118
B	KR	1992	92JYO12-13400	N/A	KU896119
B	KR	1992	Donor-R-92CIS6-13392	SG	KU896125
B	KR	1992	92KChS3-15180_15181	N/A	KX702368
B	KR	1992	92HSW8-14918_15297_14911	N/A	KX960963
B	KR	1992	92OCH3-13375_15333_14915	SG	KX960964
B	KR	1992	92HJiH1-15292_15294_15318	N/A	KX960968
B	KR	1992	92PGJ4-15289_15291_15312	N/A	KX960969
B	KR	1992	92KYS7-15322	SG	KX960970
B	KR	1992	92SJCL8-15336	N/A	KX960971
B	KR	1993	KRA812_C1	SH	AJ417420
B	KR	1993	93JHS5_HP18	PH	KF561443
B	KR	1993	HP-17_02LSP11-2268	PH	KJ140262
B	KR	1993	Donor-P-93KPS10-13295_13289	SG	KU869532
B	KR	1993	93BJR3-13326_13318	N/A	KU869582
B	KR	1993	93AJE5-13319	N/A	KU869583
B	KR	1993	93BGO2-13323_13321	N/A	KU869585
B	KR	1993	93LSY2-13324_13322	N/A	KU869586
B	KR	1993	93GHS5-13309	N/A	KU869597
B	KR	1993	93HCS2-13370_13353	N/A	KU896101
B	KR	1993	93YEH1-13398	N/A	KU896108
B	KR	1993	93JIJ6-13358	SH	KU896123
B	KR	1993	93KJK4-13359	SG	KU896124
B	KR	1993	93PCKy3-15236	N/A	KX960972
B	KR	1994	93KDG4-14929_14927_15325	N/A	KX960962
B	KR	1995	KR5076_K1X	SH	AJ417414
B	KR	1995	KR5086_C1	SH	AJ417429
B	KR	1995	HP-5_95PJH6-10862	PH	KJ140250
B	KR	1995	95KJHw4-15335	N/A	KX960974
B	KR	1996	KR3042_K5	SG	AJ417408
B	KR	1996	KR5058_K1	SH	AJ417411
B	KR	1996	KR6035_K1	SG	AJ417417
B	KR	1996	KR3026_C1	SH	AJ417426
B	KR	1997	WK	SH	AF224507
B	KR	1999	99HYH2	SH	JQ316129
B	KR	2000	00LJI12-13346	SG	KU869602
B	KR	2002	02OSG1	SG	JQ429433
B	KR	2002	HP-4_02KGJ10-4782	PH	KJ140249
B	KR	2002	HP-19_02LGS11-3443	PH	KJ140264
B	KR	2002	HP-20_02KJO10-3480	PH	KJ140265
B	KR	2003	03CWS10_1432_HP_8	PH	DQ339425
B	KR	2003	03LSH1	SH	JQ316127
B	KR	2003	03KDE11	PH	JQ316128
B	KR	2003	03HJY8	PB	JQ316131
B	KR	2003	03KGS5	PB	JQ316132
B	KR	2003	03YGS3	N/A	JQ316135
B	KR	2003	HP-15_03LSW3_6491	PH	KJ140260
B	KR	2004	04LHS6	N/A	AY839827
B	KR	2004	04LSK7	SG	DQ295192
B	KR	2004	04KMH5	SH	DQ295193
B	KR	2004	04KJin8_1955	SH	DQ295195
B	KR	2004	04KYR8	SG	DQ295196
B	KR	2004	04KMK5	PH	JQ316126
B	KR	2004	04KJS8	SB	JQ316130
B	KR	2005	05CSR3	PH	DQ837381
B	KR	2005	05YJN2	SG	JQ316134
B	KR	2007	07KYY4	SG	JQ341411
B	KR	2009	KOR_HIV_Env-1	MSM	KT878021
B	KR	2009	KOR_HIV_Env-2	SH	KT878022
B	KR	2009	KOR_HIV_Env-3	SH	KT878023
B	KR	2009	KOR_HIV_Env-4	SH	KT878024
B	KR	2009	KOR_HIV_Env-5	MSM	KT878025

B	KR	2009	KOR_HIV_Env-6	MSM	KT878026
B	KR	2009	KOR_HIV_Env-7	SH	KT878027
B	KR	2009	KOR_HIV_Env-8	SH	KT878028
B	KR	2009	KOR_HIV_Env-9	SH	KT878029
B	KR	2009	KOR_HIV_Env-10	MSM	KT878030
B	KR	2009	KOR_HIV_Env-11	SH	KT878031
B	KR	2009	KOR_HIV_Env-12	SH	KT878032
B	KR	2009	KOR_HIV_Env-13	MSM	KT878033
B	KR	2009	KOR_HIV_Env-14	N/A	KT878034
B	KR	2009	KOR_HIV_Env-15	MSM	KT878035
B	KR	2009	KOR_HIV_Env-16	N/A	KT878036
B	KR	2009	KOR_HIV_Env-17	MSM	KT878037
B	KR	2009	KOR_HIV_Env-18	MSM	KT878038
B	KR	2009	KOR_HIV_Env-19	N/A	KT878039
B	KR	2009	KOR_HIV_Env-20	SH	KT878040
B	MM	1999	mSTD101	SG	AB097870
B	SG	2008	HM039	N/A	KY213740
B	SG	2008	HM024	N/A	KY213741
B	SG	2008	HM027	N/A	KY213742
B	SG	2008	HM080	N/A	KY213743
B	SG	2008	HM053	N/A	KY213746
B	SG	2008	HM075	N/A	KY213747
B	SG	2008	HM106	N/A	KY213751
B	SG	2009	HM151	N/A	KY213744
B	SG	2009	HM141	N/A	KY213745
B	SG	2009	HM122	N/A	KY213748
B	TH	1990	BK132	N/A	AY173951
B	TH	1996	96TH_NP1538	N/A	AY713408
B	TH	1996	M140	SH	DQ354112
B	TH	1996	M041	SH	DQ354114
B	TH	1996	M081	SH	DQ354116
B	TH	1996	M145	SH	DQ354118
B	TH	1996	M149	SH	DQ354119
B	TH	1999	99TH_C1416	N/A	AY945711
B	TH	2000	00TH_C3198	N/A	AY945710
B	TH	2000	3045A06.A2	N/A	KJ952535
B	TH	2004	04TH317223	N/A	JN248321
B	TH	2004	04TH601066	N/A	JN248329
B	TH	2004	04TH803686	N/A	JN248333
B	TH	2004	04TH808998	N/A	JN248335
B	TH	2004	04TH821921	N/A	JN248337
B	TH	2005	T286588_sga01	N/A	JF297222
B	TH	2005	05TH355614	N/A	JN248343
B	TH	2005	05TH356764	N/A	JN248344
B	TH	2005	05TH356883	N/A	JN248345
B	TH	2005	05TH357801	N/A	JN248346
B	TH	2005	05TH429730	N/A	JN248347
B	TH	2005	05TH440248	N/A	JN248348
B	TH	2005	05TH645189	N/A	JN248353
B	TH	2005	05TH736580	N/A	JN248354
B	TH	2005	T286588_01	N/A	KC749001
B	TH	2006	AA010a_WG1	N/A	JX446795
B	TH	2006	AA011a09R	N/A	JX446817
B	TH	2006	AA093a_RH1	N/A	JX447795
B	TH	2006	NPBQC	N/A	KJ769147
B	TH	2007	402288_c01	N/A	JQ352781
B	TH	2007	AA040a_WG11	N/A	JX447156
B	TH	2008	T503963_sga03	N/A	JF297229
B	TH	2008	MERLBDTRC10	N/A	JN860769
B	TH	2008	AA115c05R	N/A	JX448101
B	TH	2008	T503963_01	N/A	KC749033
B	TH	2010	DEMB10TH002	MSM	KP109514
B	TH	2010	40353v04_01R	N/A	KU230423
B	TH	-	NKR_0512_8	N/A	HM215431
B	TH	-	RPW_0510_2	N/A	HM215435
B	TW	1994	TWCYS_LM49	MB	AF086817

BC	CN	1996	YNRL9613	PWID	AY967803
BC	CN	1996	YNRL9607	PWID	AY967804
BC	CN	1996	YNRL9618	PWID	AY967807
BC	CN	1996	YN3018	PWID	KF250378
BC	CN	1996	YN4007	PWID	KF250379
BC	CN	1996	YN4018	PWID	KF250380
BC	CN	1998	YNRL9828	PWID	AY967805
BC	CN	2003	BC03	PWID	EU363833
BC	CN	2003	BC09	SU	EU363839
BC	CN	2003	BC15	SU	EU363845
BC	CN	2004	CH038	PWID	EF042692
BC	CN	2005	BC11	PWID	EU363841
BC	CN	2005	05YNRL08sg	PWID	KC898976
BC	CN	2005	05YNRL09sg	PWID	KC898977
BC	CN	2005	05YNRL20sg	PWID	KC898978
BC	CN	2005	05YNRL17sg	PWID	KC899006
BC	CN	2006	BC08	PWID	EU363838
BC	CN	2006	CNE87_U	N/A	HQ700003
BC	CN	2006	06YNRL106sg	PWID	KC898981
BC	CN	2007	BC04	PWID	EU363834
BC	CN	2007	BC05	PWID	EU363835
BC	CN	2007	CNE15	PWID	HM215401
BC	CN	2007	CNE16	PWID	HM215402
BC	CN	2007	CNE18	PWID	HM215404
BC	CN	2007	CNE40	PWID	HM215414
BC	CN	2007	CNE67	PWID	HM215425
BC	CN	2007	309	SH	HM776938
BC	CN	2007	07YNLC08sg	PWID	KC898982
BC	CN	2007	07YNLC18sg	PWID	KC898983
BC	CN	2007	07YNLC22sg	PWID	KC899007
BC	CN	2007	cq070084	N/A	KF250381
BC	CN	2007	gd070112	N/A	KF250382
BC	CN	2007	gx070056	N/A	KF250383
BC	CN	2007	jx070017	N/A	KF250384
BC	CN	2007	07CNYN335	SU	KF835521
BC	CN	2007	07CNYN338	PWID	KF835524
BC	CN	2007	07CNYN339	PWID	KF835525
BC	CN	2007	07CNYN340	PWID	KF835526
BC	CN	2007	07CNYN360	PWID	KF835539
BC	CN	2007	07CNYN361	PWID	KF835540
BC	CN	2007	07CNYN367	PWID	KF835544
BC	CN	2008	08YN065	SH	GU362013
BC	CN	2009	09YNLC10sg	PWID	KC898984
BC	CN	2009	09YNLC216002sg	PWID	KC898985
BC	CN	2009	09YNLC216027sg	PWID	KC898986
BC	CN	2009	09YNLC216031sg	PWID	KC898987
BC	CN	2009	09YNLC216036sg	PWID	KC898988
BC	CN	2009	09YNLC492sg	PWID	KC898989
BC	CN	2009	09YNLC494sg	PWID	KC898990
BC	CN	2009	09YNLC496sg	PWID	KC898991
BC	CN	2009	09YNLC499sg	PWID	KC898993
BC	CN	2009	09YNYJ217036sg	PWID	KC899003
BC	CN	2009	09YNRL215025sg	PWID	KC899010
BC	CN	2009	09YNYJ217010sg	PWID	KC899013
BC	CN	2009	09YNYJ217016sg	PWID	KC899014
BC	CN	2009	09YNYJ479sg	PWID	KC899015
BC	CN	2010	JL100091	SH	KF011493
BC	CN	2010	DH04	SH	KF250397
BC	CN	2010	DH15	SH	KF250399
BC	CN	2012	DH23	MSM	KF250405
BC	CN	2012	DH24	SH	KF250406
BC	CN	2012	DH33	SH	KF250409
BC	CN	2012	DH36	SH	KF250410
BC	CN	2014	12YN10192	PWID	KT960983
BC	CN	2014	XC2014EU20	SH	KU886698
BC	CN	-	CHN19_CN19	N/A	AF268277

BC	CN	-	BC13	PWID	EU363843
BC	IN	1999	NARI10-1	N/A	EU000515
BC	IN	2002	NARI9-1	N/A	EU000506
BC	IN	2002	NARI7-1	N/A	EU000509
BC	IN	2002	INDNARI_0218440	N/A	EU000512
BC	IN	2008	Man_40	PWID	HM573466
BC	IN	2008	MAN_146	PWID	HM598635
BC	MM	1999	mIDU106	PWID	AB097869
BC	MM	1999	mIDU103	PWID	AB097873
BC	MM	2014	mKSDU81	PWID	KU820850
BC	MM	2014	mKSDU92	PWID	KU820851
BC	MM	2014	fKSDU97	PWID	KU820852
BF1	JP	2004	DR6082	N/A	AB480298
BF1	JP	2004	DR6190	N/A	AB480300
BF1	JP	-	DR0769	N/A	AB253430
BF1G	MO	2005	MO108	PWID	GU207884
C	CN	1998	YNRL9840	PWID	AY967806
C	CN	2006	CNE58	PWID	HM215421
C	CN	2006	CNE86_U	N/A	HQ699991
C	CN	2006	CNE88_U	N/A	HQ699992
C	CN	2006	06YNLC028sg	PWID	KC898980
C	CN	2006	06YNLC32sg	PWID	KC899004
C	CN	2007	CNE17	PWID	HM215403
C	CN	2007	CNE23	PWID	HM215408
C	CN	2007	CNE30	PWID	HM215411
C	CN	2007	CNE2	N/A	HQ699950
C	CN	2007	CNE65	PWID	HQ699980
C	CN	2007	CNE66	PWID	HQ699981
C	CN	2007	CNE106	N/A	HQ699985
C	CN	2007	CNE103_U	N/A	HQ699999
C	CN	2007	07CNYN328	N/A	KF835515
C	CN	2007	07CNYN336	PWID	KF835522
C	CN	2009	09YNLX090sg	PWID	KC898995
C	CN	2009	09YNLX133sg	PWID	KC898996
C	CN	2009	09YNLC216005sg	PWID	KC899005
C	CN	2009	DH21	N/A	KF250403
C	CN	2009	DH22	SH	KF250404
C	CN	2010	CNGX752_R1	N/A	KC807912
C	CN	2010	CNGX757_R1	N/A	KC807924
C	CN	2010	CNXJ0651_R1	N/A	KC807932
C	CN	2010	CNXJ1081_R1	N/A	KC807943
C	CN	2010	CNXJ09466_R2	N/A	KC807954
C	CN	2010	CNXJ0031_R1	N/A	KC807957
C	CN	2010	CNXJ09277_R1	N/A	KC807969
C	CN	2010	CNXJ1271_R6	N/A	KC807981
C	CN	2010	YNFL19	SH	KC870038
C	CN	-	GX_C_14	N/A	AY217546
C	IN	1993	93IN101	N/A	AB023804
C	IN	1993	93IN999	SG	AF067154
C	IN	1993	93IN904	PB	AF067157
C	IN	1993	93IN905	PB	AF067158
C	IN	1994	94IN11246	SH	AF067159
C	IN	1994	94IN476	N/A	AF286223
C	IN	1994	94IN_20635_4	N/A	AY713414
C	IN	1995	95IN21068	SH	AF067155
C	IN	1998	98IN012	N/A	AF286231
C	IN	1998	98IN022	N/A	AF286232
C	IN	1999	01IN565_10	PB	AY049708
C	IN	1999	HIV_16055_2	SH	EF117268
C	IN	1999	HIV_16845_2	SH	EF117269
C	IN	1999	HIV_16936_2	SH	EF117270
C	IN	1999	HIV_25710_2	SH	EF117271
C	IN	1999	HIV_25711_2	SH	EF117272
C	IN	1999	HIV_25925_2	SH	EF117273
C	IN	2000	HIV_00836_2	SH	EF117265
C	IN	2000	HIV_001428_2	SH	EF117266

C	IN	2000	HIV_0013095_2	SH	EF117267
C	IN	2000	HIV_26191_2	SH	EF117274
C	IN	2000	NARI_VB105_J10	SH	EU521729
C	IN	2003	D24	N/A	EF469243
C	IN	2004	C.IN.04.NIRT379.1	N/A	KF766537
C	IN	2005	C.IN.05.NIRT333.1	N/A	KF766540
C	IN	2005	C.IN.05.NIRT723.1	N/A	KF766541
C	IN	2007	NARI_VB52_J19	SH	EU521727
C	IN	2007	NARI_IVC4_NEM_J2	SH	EU908218
C	IN	2009	T125_p1	SH	JF680935
C	IN	2011	NIRT009	N/A	KX069227
C	IN	2011	NIRT010	N/A	KX069228
C	IN	2013	NIRT_ENV003	MB	KX756602
C	IN	2013	NIRT_ENV011	MB	KX756610
C	IN	2013	NIRT_ENV012	MB	KX756611
C	IN	2013	NIRT_ENV013	MB	KX756612
C	IN	2013	NIRT_ENV014	MB	KX756613
C	IN	2013	NIRT_ENV015	MB	KX756614
C	IN	2013	NIRT_ENV017	MB	KX756615
C	IN	2014	NIRT004	N/A	KX069222
C	IN	2014	NIRT_ENV001	MB	KX756600
C	IN	2014	NIRT_ENV002	MB	KX756601
C	IN	2014	NIRT_ENV004	MB	KX756603
C	IN	2014	NIRT_ENV005	MB	KX756604
C	IN	2014	NIRT_ENV006	MB	KX756605
C	IN	2014	NIRT_ENV007	MB	KX756606
C	IN	2014	NIRT_ENV008	MB	KX756607
C	IN	2014	NIRT_ENV009	MB	KX756608
C	IN	2014	NIRT_ENV010	MB	KX756609
C	IN	2015	NIRT001	N/A	KX069219
C	IN	2015	NIRT002	N/A	KX069220
C	IN	2015	NIRT003	N/A	KX069221
C	IN	2015	NIRT005	N/A	KX069223
C	IN	2015	NIRT006	N/A	KX069224
C	IN	2015	NIRT007	N/A	KX069225
C	IN	2015	NIRT008	N/A	KX069226
C	IN	-	CALCMANDAL	N/A	AJ276221
C	IN	-	cal308F	SW	DQ404010
C	IN	-	VB39	N/A	EF694032
C	IN	-	VB49	N/A	EF694034
C	IN	-	NARI_VB96_J21	N/A	EU521728
C	MM	1999	mIDU101_3	PWID	AB097871
C	NP	2011	11NP003	SH	KJ158421
C	NP	2011	11NP007	PWID	KJ158423
C	NP	2011	11NP008	MB	KJ158424
C	NP	2011	11NP010	SH	KJ158425
C	NP	2011	11NP014	SH	KJ158426
C	NP	2011	11NP028	SH	KJ158428
C	NP	2011	11NP041	PWID	KJ158430
C	NP	2011	11NP046	PWID	KJ158431
C	NP	2011	11NP065	SH	KJ158432
C	NP	2011	11NP068	PWID	KJ158433
C	NP	2011	11NP069	MB	KJ158434
C	NP	2011	11NP080	SH	KJ158435
C	NP	2011	11NP092	MSM	KJ158438
C	NP	2011	11NP093	SH	KJ158439
C	NP	2011	11NP102	SH	KJ158440
C	NP	2011	11NP015	SH	KJ541837
C	NP	2011	11NP071	PWID	KJ541839
C	NP	2011	11NP074	SH	KJ541840
C	NP	2011	11NP075	SH	KJ541841
C	NP	2011	11NP082	SH	KJ541844
C	NP	2011	11NP095	SH	KJ541846
C	NP	2011	11NP103	SH	KJ541847
C	NP	2011	11NP104	SH	KJ541848
C	NP	2011	11NP107	SH	KJ541849

C	PK	2014	DEMC14PK009	SH	KU749412
C	PK	2014	PK010	MSM	KX232603
C	TH	-	PWJ_0513_39	N/A	HM215433
CD	NP	2011	11NP035	MB	KJ158429
CD	NP	2011	11NP079	SH	KJ541843
CU	JP	2004	DR5782	N/A	AB286849
D	KR	2004	04KBH8	SG	DQ054367
G	CN	2006	sh52	N/A	HM067749
G	CN	2008	GX_2084_08	N/A	JN106043
-	CN	2014	YN10134	SH	KY406739
-	JP	1986	JH32	PH	M21138
-	PH	2015	DE00115PH012	SG	KY658688
-	PH	2015	DEMB15PH002	SG	KY658689
-	PH	2015	DEMB15PH003	SG	KY658690
-	PH	2015	DEURF15PH005	SG	KY658691
-	PH	2015	DEURF15PH001	SG	KY658692
-	TH	1992	TH921104	SH	U39255
-	TH	1993	93TH067	N/A	U39258

Guide to abbreviations:

**Country**	
CN	China
HK	Hong Kong
ID	Indonesia
IN	India
JP	Japan
KH	Cambodia
KR	South Korea
MM	Myanmar
MO	Macau
MY	Malaysia
NP	Nepal
PH	Philippines
PK	Pakistan
SG	Singapore
TH	Thailand
TW	Taiwan
VN	Vietnam

**Risk Factor**	
EX	Experimental
MB	Mother to child
MSM	Men who have sex with men
N/A	Not recorded, unknown, other
NO	Nosocomial
PB	Blood transfusion
PH	Hemophiliac
PWID	People who inject drugs
SB	Bisexual
SG	Homosexual
SH	Heterosexual
SU	Sexual transmission, unknown type
SW	Sex worker

Table S3. Socio-demographic characteristics of SEARCH 010/RV254 participants

**Characteristics**	
**N**	303
**Age**	
Mean (SD)	28.0 (7.5)
Median (IQR)	26 (23-31)
Range	18-57
**Gender**	
Male (%)	291 (96.0%)
Female (%)	12 (4.0%)
**Days from first documented positive HIV RNA**	
Mean (SD)	2.34 (0.99)
Median (IQR)	2 (2-3)
Range	0-8
**HIV RNA (log10 copies/ml)**	
Mean (SD)	5.78 (1.03)
Median (IQR)	5.80 (5.23-6.69)
Range	2.78-7.85
**CD4 T cells (cells/mm3)**	
Mean (SD)	408.6 (188.7)
Median (IQR)	381 (271-515)
Range	7-1236
**Occupation, n (%)**	
Private business/factory employee	113 (37.3%)
Student	73 (24.1%)
Unemployed	31 (10.2%)
Business owner	29 (9.6%)
Government officer	22 (7.3%)
Sex worker	10 (3.3%)
Soldier/police	3 (1.0%)
Agriculture	2 (0.7%)
Taxi/Truck driver	2 (0.7%)
Homemaker	1 (0.3%)
Laborer	1 (0.3%)
Other	16 (5.3%)
**Number of sexual partners in the past month**	
Mean (SD)	2.3 (2.0)
Median (IQR)	2 (1-3)
Range	1-20
**Risk factor**	
Homosexual male (%)	265 (87.5%)
Bisexual male (%)	14 (4.6%)
Heterosexual male (%)	12 (4.0%)
Heterosexual female (%)	12 (4.0%)
People who inject drugs (%)	0 (0%)

Table S4. RV254 Subtype B V3 loop tip motifs and their global frequencies

Deduced V3 motif (HXB2 7158-7169)	Frequency of V3 motif in Subtype B Strains (LANL, n=122,265)	RV254 Participants with Subtype B Strains
GPGR (Western B consensus)	67.68%	2544072, 2545192, 2548411, 2549720
APGR	4.06%	2544011, 2544829
GPGG	3.04%	2547627
APGQ	0.02%	2548419
GPGQ (Thai B′ consensus)	1.31%	None

Table S5. Clusters identified in Asian HIV-1 transmission network

Cluster	Nodes	Country	Subtype	Year	Risk
1	15	VN (11), CN (4)	01_AE (15)	1997 (5), 1998 (8), 1999 (1), N/A (1)	N/A (12), PWID (2), NO (1)
2	14	TH (10), JP (2), ID (1), VN (1)	01_AE (14)	1990 (4), 1992 (2), 1993 (7), 1997 (1)	SH (7), N/A (5), PWID (2)
3	12	CN (12)	07_BC (12)	2007 (7), 2008 (1), 2009 (1), N/A (3)	MSM (9), N/A (3)
4	10	CN (10)	B (10)	2002 (3), 2003 (1), 2004 (1), 2012 (4), N/A (1)	N/A (7), NO (1), PWID (1), SH (1)
5	7	CN (7)	07_BC (7)	1997 (1), 1998 (1), 2005 (1), 2006 (2), N/A (2)	PWID (5), N/A (2)
6	6	CN (6)	01_AE (6)	2005 (2), 2007 (4)	MSM (4), N/A (2)
7	6	MM (6)	83_cpx (6)	2013 (6)	PWID (6)
8	6	CN (6)	08_BC (5), C (1)	1997 (3), 1998 (1), 2007 (1), N/A (1)	PWID (4), N/A (2)
9	6	TH (6)	01_AE (6)	2009 (6)	N/A (6)
10	5	CN (5)	01_AE (5)	2008 (1), 2009 (2), 2010 (2)	MSM (5)
11	5	MM (5)	82_cpx (5)	2013 (5)	PWID (5)
12	5	JP (5)	B (5)	2003 (1), 2004 (1), 2005 (1), 2006 (2)	N/A (5)
13	5	TH (5)	01_AE (5)	1999 (3), 2000 (2)	PWID (5)
14	4	CN (4)	08_BC (4)	2007 (4)	N/A (4)
15	4	CN (4)	08_BC (4)	2007 (4)	N/A (2), PWID (2)
16	4	CN (4)	08_BC (3), C (1)	2007 (4)	N/A (3), PWID (1)
17	3	JP (3)	01_AE (3)	N/A (3)	N/A (3)
18	3	CN (3)	01_AE (3)	2007 (1), 2008 (1), 2010 (1)	MSM (2), SH (1)
19	3	CN (3)	08_BC (3)	2007 (3)	N/A (3)
20	3	CN (3)	01_AE (3)	2002 (3)	SH (3)
21	3	JP (3)	69_01B (3)	2003 (1), 2004 (2)	MSM (3)
22	3	CN (3)	B (3)	2005 (1), 2006 (1), 2007 (1)	N/A (2), PWID (1)
23	3	CN (3)	01_AE (3)	2010 (2), 2011 (1)	MSM (3)
24	3	CN (3)	BC (3)	1996 (3)	PWID (3)
25	3	CN (3)	01_AE (3)	2008 (1), 2009 (2)	MSM (3)
26	3	TH (3)	01_AE (3)	2006 (3)	N/A (3)
27	3	TH (3)	01_AE (3)	1999 (1), N/A (2)	N/A (3)
28	3	CN (3)	08_BC (3)	2007 (3)	N/A (2), PWID (1)
29	3	CN (3)	01_AE (3)	2007 (3)	N/A (2), PWID (1)
30	3	CN (3)	07_BC (3)	2007 (3)	N/A (2), SH (1)
31	3	TH (3)	01_AE (3)	2009 (3)	N/A (3)
32	3	CN (3)	55_01B (3)	2010 (1), 2011 (2)	MSM (3)
33	3	MM (3)	83_cpx (3)	2013 (3)	PWID (3)
34	3	KR (3)	B (3)	1992 (1), 1993 (1), 1995 (1)	N/A (3)
35	3	CN (3)	01_AE (3)	2008 (1), 2009 (2)	MSM (3)
36	3	KR (3)	B (3)	1991 (1), 1992 (1), 1993 (1)	N/A (2), SH (1)
37	2	TH (2)	01_AE (2)	2015 (2)	MSM (2)
38	2	MY (1), SG (1)	51_01B (2)	2009 (1), 2011 (1)	N/A (1), SG (1)
39	2	SG (2)	B (2)	2008 (1), 2009 (1)	N/A (2)
40	2	SG (2)	01_AE (2)	2008 (2)	N/A (2)
41	2	TH (2)	01B (2)	2001 (1), 2002 (1)	PWID (2)
42	2	CN (2)	B (2)	2004 (2)	N/A (2)
43	2	CN (2)	08_BC (2)	2007 (2)	N/A (1), PWID (1)
44	2	KR (2)	B (2)	1991 (1), 1992 (1)	N/A (2)
45	2	PK (2)	C (2)	2014 (2)	MSM (1), SH (1)
46	2	CN (2)	64_BC (2)	2009 (2)	PWID (2)
47	2	TH (2)	01_AE (2)	1993 (2)	N/A (2)
48	2	CN (2)	01_AE (2)	2007 (2)	N/A (1), PWID (1)
49	2	TH (2)	01_AE (2)	2006 (2)	N/A (2)
50	2	KR (2)	B (2)	1992 (2)	N/A (2)
51	2	TH (2)	01_AE (2)	2010 (1), 2013 (1)	MSM (2)
52	2	CN (2)	01B (2)	2013 (2)	MSM (2)
53	2	PK (2)	A1 (2)	2014 (1), 2015 (1)	PWID (2)
54	2	CN (2)	BC (2)	1996 (2)	PWID (2)
55	2	TH (2)	01B (2)	2013 (1), 2015 (1)	MSM (2)
56	2	TH (2)	B (2)	2005 (2)	N/A (2)
57	2	CN (2)	01_AE (1), 0107 (1)	2007 (2)	MSM (1), SG (1)
58	2	TH (2)	01_AE (2)	2005 (1), 2008 (1)	N/A (2)
59	2	TH (2)	01_AE (2)	2004 (2)	N/A (2)
60	2	TH (2)	01_AE (2)	2006 (2)	N/A (2)

61	2	CN (2)	BC (2)	2009 (2)	PWID (2)
62	2	CN (2)	65_cpx (2)	2009 (1), 2010 (1)	SH (2)
63	2	JP (2)	B (2)	2002 (2)	N/A (2)
64	2	CN (2)	01_AE (2)	2002 (2)	PWID (1), SH (1)
65	2	JP (2)	B (2)	N/A (2)	N/A (2)
66	2	TH (2)	B (2)	2005 (2)	N/A (2)
67	2	TH (2)	01_AE (2)	2009 (1), 2010 (1)	MSM (2)
68	2	TH (2)	01_AE (2)	2000 (2)	N/A (2)
69	2	CN (2)	01_AE (2)	2005 (2)	SH (2)
70	2	KR (2)	B (2)	1993 (1), 1995 (1)	PH (1), SG (1)
71	2	CN (2)	01_AE (2)	2007 (2)	SH (2)
72	2	CN (2)	01_AE (2)	2007 (1), 2009 (1)	MSM (1), SH (1)
73	2	CN (2)	07_BC (2)	N/A (2)	N/A (2)
74	2	CN (2)	B (2)	2004 (2)	N/A (1), PB (1)
75	2	CN (2)	01_AE (2)	2009 (1), 2010 (1)	MSM (2)
76	2	TH (2)	01_AE (2)	2006 (2)	N/A (2)
77	2	SG (2)	01_AE (2)	2008 (1), 2009 (1)	N/A (2)
78	2	JP (2)	B (2)	N/A (2)	N/A (2)
79	2	JP (2)	B (1), BF (1)	2004 (1), N/A (1)	N/A (2)
80	2	TH (2)	01_AE (2)	2006 (2)	N/A (2)
81	2	CN (2)	85_BC (2)	2014 (2)	SH (2)
82	2	TH (2)	01B (2)	2006 (2)	N/A (2)
83	2	MY (2)	58_01B (2)	2011 (2)	PWID (2)
84	2	TH (2)	01_AE (2)	2000 (1), N/A (1)	N/A (2)
85	2	JP (2)	01_AE (2)	N/A (2)	N/A (2)
86	2	TH (2)	01_AE (2)	2006 (2)	N/A (2)
87	2	TH (2)	34_01B (2)	1999 (2)	PWID (2)
88	2	TH (2)	01_AE (2)	2006 (2)	N/A (2)
89	2	CN (2)	BC (1), C (1)	2007 (2)	PWID (2)
90	2	CN (2)	07_BC (2)	2004 (1), 2006 (1)	PWID (2)
91	2	CN (2)	01_AE (2)	2006 (2)	N/A (1), PWID (1)
92	2	TH (2)	01_AE (2)	2002 (2)	PWID (2)
93	2	TH (2)	01B (2)	2005 (1), 2007 (1)	N/A (2)
94	2	TH (2)	01_AE (2)	1999 (2)	N/A (1), PWID (1)
95	2	TH (2)	01_AE (2)	2005 (1), 2006 (1)	N/A (1), SH (1)
96	2	TH (2)	01_AE (2)	1999 (1), N/A (1)	N/A (2)
97	2	CN (2)	07_BC (2)	2007 (2)	SH (2)
98	2	TH (2)	01_AE (2)	2009 (2)	N/A (2)
99	2	CN (2)	08_BC (1), C (1)	2007 (2)	N/A (1), PWID (1)
100	2	CN (2)	08_BC (2)	2007 (2)	N/A (2)
101	2	TW (2)	07_BC (1), 07B (1)	2004 (2)	PWID (2)
102	2	TH (2)	01_AE (2)	2006 (2)	N/A (2)
103	2	CN (2)	07_BC (1), BC (1)	2007 (2)	N/A (1), PWID (1)
104	2	CN (2)	B (2)	2004 (2)	N/A (1), SH (1)
105	2	TH (2)	01_AE (2)	1999 (1), 2000 (1)	N/A (2)
106	2	TH (2)	01_AE (2)	1999 (1), 2000 (1)	N/A (2)
107	2	CN (2)	07_BC (2)	2005 (2)	PWID (1), SH (1)
108	2	JP (2)	B (2)	N/A (2)	N/A (2)
109	2	CN (2)	08_BC (2)	2007 (2)	N/A (1), PWID (1)
110	2	TH (2)	01_AE (2)	2006 (2)	N/A (2)
111	2	TH (2)	B (2)	2010 (2)	MSM (1), N/A (1)
112	2	KR (2)	B (2)	2009 (2)	MSM (1), N/A (1)
113	2	KR (2)	B (2)	2009 (2)	MSM (1), SH (1)
114	2	TH (2)	01_AE (2)	2009 (2)	MSM (2)
115	2	CN (2)	01_AE (2)	2007 (1), 2010 (1)	MSM (1), SH (1)
116	2	CN (2)	01_AE (2)	2007 (2)	PWID (2)
117	2	CN (2)	01_AE (2)	2005 (1), 2009 (1)	N/A (1), SH (1)
118	2	CN (2)	01_AE (2)	2005 (1), 2006 (1)	N/A (1), PWID (1)
119	2	CN (2)	59_01B (2)	2009 (1), 2011 (1)	MSM (2)
120	2	IN (2)	C (2)	2014 (1), 2015 (1)	MB (1), N/A (1)
121	2	TH (2)	01_AE (2)	2001 (2)	PWID (2)
122	2	TH (2)	01_AE (2)	1999 (2)	PWID (2)
123	2	JP (2)	69_01B (1), 01B (1)	2003 (1), 2005 (1)	MSM (2)

Table S6. Nodes in Asian HIV-1 transmission network

a) Summary of nodes in transmission network (n=348)

**Country**	**n**	**%**
China (CN)	162	46.6%
Thailand (TH)	98	28.2%
Japan (JP)	27	7.8%
South Korea (KR)	16	4.6%
Myanmar (MM)	14	4.0%
Vietnam (VN)	12	3.4%
Singapore (SG)	7	2.0%
Pakistan (PK)	4	1.1%
Malaysia (MY)	3	0.9%
India (IN)	2	0.6%
Taiwan (TW)	2	0.6%
Indonesia (ID)	1	0.3%
**Subtype**	**n**	**%**
01_AE	162	46.6%
B	57	16.4%
07_BC	32	9.2%
08_BC	29	8.3%
01B	11	3.2%
83_cpx	9	2.6%
BC	9	2.6%
C	8	2.3%
82_cpx	5	1.4%
69_01B	4	1.1%
55_01B	3	0.9%
34_01B	2	0.6%
51_01B	2	0.6%
58_01B	2	0.6%
59_01B	2	0.6%
64_BC	2	0.6%
65_cpx	2	0.6%
85_BC	2	0.6%
A1	2	0.6%
0107	1	0.3%
07B	1	0.3%
BF1	1	0.3%
**Risk Factor**	**n**	**%**
Unknown (N/A)	167	48.0%
People who inject drugs (PWID)	80	23.0%
Men who have sex with men (MSM)	60	17.2%
Heterosexual (SH)	33	9.5%
Homosexual (SG)	3	0.9%
Nosocomial (NO)	2	0.6%
Mother to child (MB)	1	0.3%
Blood transfusion (PB)	1	0.3%
Hemophiliac (PH)	1	0.3%
**Sampling Year**	**n**	**%**
1990	4	1.1%
1991	2	0.6%
1992	7	2.0%
1993	12	3.4%
1995	2	0.6%
1996	5	1.4%
1997	10	2.9%
1998	10	2.9%
1999	14	4.0%
2000	7	2.0%
2001	3	0.9%
2002	13	3.7%
2003	4	1.1%
2004	16	4.6%
2005	19	5.5%

2006	31	8.9%
2007	63	18.1%
2008	10	2.9%
2009	37	10.6%
2010	13	3.7%
2011	7	2.0%
2012	4	1.1%
2013	18	5.2%
2014	6	1.7%
2015	5	1.4%
N/A	26	7.5%

b) Detailed list of nodes in transmission network

Network ID	Country Code	Subtype	Sampling Year	Risk Factor	Name	Accession
CN001	CN	0107	2007	SG	JL070032	KC990127
CN002	CN	01_AE	1997	PWID	97CNGX2F_97CNGX_2F	AY008714
CN003	CN	01_AE	1997	PWID	97CNGX_11F	AY008718
CN004	CN	01_AE	N/A	N/A	GX_E_14	AY217545
CN005	CN	01_AE	2005	SH	FJ051	DQ859178
CN006	CN	01_AE	1999	NO	AE01	EU363849
CN007	CN	01_AE	2007	N/A	BJ5_11	GU475024
CN008	CN	01_AE	2005	SH	05GX002	GU564222
CN009	CN	01_AE	2007	MSM	BJOX015000_11_5	HM215377
CN010	CN	01_AE	2007	MSM	BJOX018000_02_3	HM215379
CN011	CN	01_AE	2007	MSM	BJOX021000_03_3	HM215383
CN012	CN	01_AE	2007	MSM	BJOX025000_01_1	HM215386
CN013	CN	01_AE	2007	PWID	CNE28	HM215409
CN014	CN	01_AE	2007	PWID	CNE56	HM215419
CN015	CN	01_AE	2006	PWID	CNE59	HM215422
CN016	CN	01_AE	2005	MSM	CNE62	HM215424
CN017	CN	01_AE	2006	PWID	CNE8	HM215427
CN018	CN	01_AE	2007	N/A	CNE26	HQ699955
CN019	CN	01_AE	2007	N/A	CNE27	HQ699956
CN020	CN	01_AE	2005	N/A	CNE60	HQ699977
CN021	CN	01_AE	2007	PWID	CNE107	HQ699986
CN022	CN	01_AE	2005	N/A	CNE61_U	HQ699987
CN023	CN	01_AE	2006	N/A	CNE91_U	HQ699993
CN024	CN	01_AE	2010	MSM	CYM105	JX112798
CN025	CN	01_AE	2010	MSM	CYM152	JX112807
CN026	CN	01_AE	2007	SH	FJ070013	JX112810
CN027	CN	01_AE	2007	SH	FJ070039	JX112816
CN028	CN	01_AE	2007	N/A	GZ070015	JX112841
CN029	CN	01_AE	2007	PWID	GZ070016	JX112842
CN030	CN	01_AE	2007	SH	JS071004	JX112852
CN031	CN	01_AE	2007	SH	LN070013	JX112856
CN032	CN	01_AE	2007	MSM	TJ070003	JX112859
CN033	CN	01_AE	2002	SH	YN0203	JX112860
CN034	CN	01_AE	2002	SH	YN0225	JX112862
CN035	CN	01_AE	2002	PWID	YN0229	JX112863
CN036	CN	01_AE	2002	SH	YN0232	JX112864
CN037	CN	01_AE	2002	SH	YN0235	JX112865
CN038	CN	01_AE	2009	N/A	ZK052	JX112869
CN039	CN	01_AE	2009	MSM	09LNA008	JX960604
CN040	CN	01_AE	2009	MSM	09LNA011	JX960605
CN041	CN	01_AE	2008	MSM	08LNA003	JX960606
CN042	CN	01_AE	2010	MSM	10LNA124	JX960609
CN043	CN	01_AE	2010	MSM	10LNA819	JX960610
CN044	CN	01_AE	2008	MSM	08LNA002	JX960612
CN045	CN	01_AE	2009	MSM	09LNA020	JX960613
CN046	CN	01_AE	2009	MSM	09LNA230	JX960614
CN047	CN	01_AE	2008	MSM	08LNA004	JX960617
CN048	CN	01_AE	2009	MSM	09LNA041	JX960618
CN049	CN	01_AE	2010	MSM	10LNA294	JX960619
CN050	CN	01_AE	2010	MSM	10LNA918	JX960620
CN051	CN	01_AE	2009	MSM	09LNA425	JX960621
CN052	CN	01_AE	2009	MSM	09LNA013	JX960623

CN053	CN	01_AE	2010	MSM	10LNA669	JX960624
CN054	CN	01_AE	2011	MSM	DE00111CN003	KC596065
CN055	CN	01_AE	2008	SH	BJOX033000.e01	KM218302
CN056	CN	01_AE	2009	MSM	DE00109CN005	KP109504
CN057	CN	01_AE	2005	SH	GX2005002	KP178420
CN058	CN	01B	2013	MSM	BJMP3037B	KP418805
CN059	CN	01B	2013	MSM	BJMP3194B	KP418806
CN060	CN	07_BC	1997	PWID	97CN001_C54	AF286226
CN061	CN	07_BC	1998	N/A	98CN009	AF286230
CN062	CN	07_BC	N/A	N/A	CNGL179	AF503396
CN063	CN	07_BC	2005	SH	XJDC6441	EF368370
CN064	CN	07_BC	2005	PWID	XJN0084	EF368371
CN065	CN	07_BC	2005	PWID	pXJDC6441-2	EF420986
CN066	CN	07_BC	N/A	PWID	BC02_XJ74_2	EU363832
CN067	CN	07_BC	2007	MSM	BJOX011000_01_3	HM215375
CN068	CN	07_BC	2007	MSM	BJOX019000_02_1	HM215380
CN069	CN	07_BC	2007	MSM	BJOX027000_02_1	HM215388
CN070	CN	07_BC	2007	MSM	BJOX029000_03_1	HM215392
CN071	CN	07_BC	2007	SH	CNE19	HM215405
CN072	CN	07_BC	2007	SH	CNE20	HM215406
CN073	CN	07_BC	N/A	N/A	BJ22_5	HQ326125
CN074	CN	07_BC	N/A	N/A	XJ16_6	HQ326133
CN075	CN	07_BC	N/A	N/A	XJ47_5	HQ326134
CN076	CN	07_BC	N/A	N/A	GX33m_25	HQ326141
CN077	CN	07_BC	N/A	N/A	GX45_57	HQ326143
CN078	CN	07_BC	2007	N/A	CNE39	HQ699965
CN079	CN	07_BC	2007	SH	CNE68	HQ699982
CN080	CN	07_BC	2007	N/A	CNE69	HQ699983
CN081	CN	07_BC	2007	N/A	CNE70	HQ699984
CN082	CN	07_BC	2004	PWID	CBJA025	JF346905
CN083	CN	07_BC	2006	PWID	Sichuan_2006_SC020	JX392380
CN084	CN	07_BC	2006	PWID	Sichuan_2006_SC025	JX392381
CN085	CN	07_BC	2006	PWID	Sichuan_2006_SC124	JX392382
CN086	CN	07_BC	2009	MSM	09LNA446	JX960600
CN087	CN	07_BC	2007	MSM	BJ070032	KF250366
CN088	CN	07_BC	2007	MSM	GZ070087	KF250372
CN089	CN	07_BC	2007	MSM	BJOX016000.e02	KM217857
CN090	CN	07_BC	2008	MSM	BJOX037000.e01	KM218157
CN091	CN	08_BC	1998	N/A	98CN006	AF286229
CN092	CN	08_BC	1997	PWID	97CNGX_6F	AY008715
CN093	CN	08_BC	1997	PWID	97CNGX_7F	AY008716
CN094	CN	08_BC	1997	PWID	97CNGX_9F	AY008717
CN095	CN	08_BC	2007	PWID	CNE52	HM215416
CN096	CN	08_BC	2007	PWID	CNE53	HM215417
CN097	CN	08_BC	2007	N/A	CNE22	HQ699952
CN098	CN	08_BC	2007	N/A	CNE24	HQ699953
CN099	CN	08_BC	2007	N/A	CNE25	HQ699954
CN100	CN	08_BC	2007	N/A	CNE29	HQ699957
CN101	CN	08_BC	2007	N/A	CNE32	HQ699958
CN102	CN	08_BC	2007	N/A	CNE33	HQ699959
CN103	CN	08_BC	2007	N/A	CNE35	HQ699961
CN104	CN	08_BC	2007	N/A	CNE36	HQ699962
CN105	CN	08_BC	2007	N/A	CNE37	HQ699963
CN106	CN	08_BC	2007	N/A	CNE38	HQ699964
CN107	CN	08_BC	2007	N/A	CNE41	HQ699966
CN108	CN	08_BC	2007	N/A	CNE42	HQ699967
CN109	CN	08_BC	2007	N/A	CNE43	HQ699968
CN110	CN	08_BC	2007	N/A	CNE44	HQ699969
CN111	CN	08_BC	2007	N/A	CNE45	HQ699970
CN112	CN	08_BC	2007	PWID	CNE46	HQ699971
CN113	CN	08_BC	2007	PWID	CNE47	HQ699972
CN114	CN	08_BC	2007	N/A	CNE48	HQ699973
CN115	CN	08_BC	2007	PWID	CNE49	HQ699974
CN116	CN	08_BC	2007	N/A	CNE50	HQ699975
CN117	CN	08_BC	2007	N/A	CNE54	HQ699976
CN118	CN	08_BC	2007	N/A	CNE51	HQ700004

CN119	CN	08_BC	2007	PWID	2007CNGX_HK	JF719819
CN120	CN	55_01B	2010	MSM	HNCS102056	JX574661
CN121	CN	55_01B	2011	MSM	11HNCS500434	KF927150
CN122	CN	55_01B	2011	MSM	11CN.GDDG325	KF927151
CN123	CN	59_01B	2009	MSM	09LNA423	JX960635
CN124	CN	59_01B	2011	MSM	11LNSY300392	KC462190
CN125	CN	64_BC	2009	PWID	YNFL08	KC870031
CN126	CN	64_BC	2009	PWID	09YNLX047sg	KC898994
CN127	CN	65_cpx	2010	SH	YNFL02	KC870028
CN128	CN	65_cpx	2009	SH	YNFL05	KC870030
CN129	CN	85_BC	2014	SH	14CN_SCYB3	KU992931
CN130	CN	85_BC	2014	SH	14CN_SCYB11	KU992935
CN131	CN	B	2004	N/A	CHNHLJSM0417c3	AY905496
CN132	CN	B	2004	N/A	CHNHLJBF04016c4	AY905497
CN133	CN	B	2002	N/A	02HNsmx2	DQ007901
CN134	CN	B	2002	N/A	02HNsq4	DQ007902
CN135	CN	B	2002	N/A	02HNsc11	DQ007903
CN136	CN	B	2003	NO	B02	EU363826
CN137	CN	B	2004	SH	CNE12	HM215399
CN138	CN	B	2004	SH	CNE14	HM215400
CN139	CN	B	2006	PWID	CNE6	HM215423
CN140	CN	B	2004	N/A	CNE13	HQ699951
CN141	CN	B	2004	N/A	CNE63	HQ699978
CN142	CN	B	2004	PB	CNE64	HQ699979
CN143	CN	B	2005	N/A	CNE101_U	HQ699998
CN144	CN	B	2007	N/A	CNE105_U	HQ700000
CN145	CN	B	2012	N/A	2124-0-1	KX693054
CN146	CN	B	2012	N/A	2259-1	KX693100
CN147	CN	B	2012	N/A	2360-0-1a	KX693166
CN148	CN	B	2012	N/A	3617-1	KX693357
CN149	CN	B	N/A	PWID	RL42	U71182
CN150	CN	BC	1996	PWID	YNRL9607	AY967804
CN151	CN	BC	1996	PWID	YNRL9618	AY967807
CN152	CN	BC	2007	PWID	CNE40	HM215414
CN153	CN	BC	2007	PWID	07YNLC18sg	KC898983
CN154	CN	BC	2009	PWID	09YNLC10sg	KC898984
CN155	CN	BC	2009	PWID	09YNLC494sg	KC898990
CN156	CN	BC	1996	PWID	YN3018	KF250378
CN157	CN	BC	1996	PWID	YN4007	KF250379
CN158	CN	BC	1996	PWID	YN4018	KF250380
CN159	CN	C	N/A	N/A	GX_C_14	AY217546
CN160	CN	C	2007	PWID	CNE23	HM215408
CN161	CN	C	2007	PWID	CNE30	HM215411
CN162	CN	C	2007	PWID	CNE65	HQ699980
ID001	ID	01_AE	1993	N/A	ID17	AB485652
IN001	IN	C	2015	N/A	NIRT002	KX069220
IN002	IN	C	2014	MB	NIRT_ENV009	KX756608
JP001	JP	01_AE	1993	SH	93JP_NH1	AB052995
JP002	JP	01_AE	1993	SH	NH25_93JPNH25T_93JP_NH2_5T	AB070352
JP003	JP	01_AE	N/A	N/A	DR0492	AB253423
JP004	JP	01_AE	N/A	N/A	DR1741	AB253635
JP005	JP	01_AE	N/A	N/A	DR1873	AB253647
JP006	JP	01_AE	N/A	N/A	DR1236	AB253692
JP007	JP	01_AE	N/A	N/A	DR2192	AB253703
JP008	JP	01B	2003	MSM	03JP-5091K279	AB859012
JP009	JP	69_01B	2003	MSM	03JP-5091K231	AB845344
JP010	JP	69_01B	2004	MSM	04JP-5091K320	AB845345
JP011	JP	69_01B	2004	MSM	04JP-5091K323	AB845346
JP012	JP	69_01B	2005	MSM	05JP-5091K448	AB845347
JP013	JP	B	2004	N/A	DR6089	AB286955
JP014	JP	B	2002	N/A	194	AB428553
JP015	JP	B	2002	N/A	197	AB428554
JP016	JP	B	2003	N/A	285	AB428558
JP017	JP	B	2004	N/A	329	AB428559
JP018	JP	B	2005	N/A	398	AB428560
JP019	JP	B	2006	N/A	472	AB428561

JP020	JP	B	2006	N/A	574	AB428562
JP021	JP	B	N/A	N/A	DR1673	AB564744
JP022	JP	B	N/A	N/A	DR2735	AB564746
JP023	JP	B	N/A	N/A	pJPDR0796B02	AB565478
JP024	JP	B	N/A	N/A	JRC03B	AB565495
JP025	JP	B	N/A	N/A	JRC53B	AB565499
JP026	JP	B	N/A	N/A	DR5929	AB604950
JP027	JP	BF1	N/A	N/A	DR0769	AB253430
KR001	KR	B	1995	PH	HP-5_95PJH6-10862	KJ140250
KR002	KR	B	2009	MSM	KOR_HIV_Env-1	KT878021
KR003	KR	B	2009	MSM	KOR_HIV_Env-13	KT878033
KR004	KR	B	2009	N/A	KOR_HIV_Env-14	KT878034
KR005	KR	B	2009	SH	KOR_HIV_Env-20	KT878040
KR006	KR	B	1993	SG	Donor-P-93KPS10-13295_13289	KU869532
KR007	KR	B	1992	SH	92KYJ5-13316	KU869580
KR008	KR	B	1993	N/A	93GHS5-13309	KU869597
KR009	KR	B	1992	N/A	92DGi6-13344	KU869604
KR010	KR	B	1992	N/A	92JYO12-13400	KU896119
KR011	KR	B	1991	N/A	91KYB12-15320_15293_15314	KX960966
KR012	KR	B	1991	N/A	91LGJ11-15285_15339_15331	KX960967
KR013	KR	B	1992	N/A	92HJiH1-15292_15294_15318	KX960968
KR014	KR	B	1992	N/A	92PGJ4-15289_15291_15312	KX960969
KR015	KR	B	1993	N/A	93PCKy3-15236	KX960972
KR016	KR	B	1995	N/A	95KJHw4-15335	KX960974
MM001	MM	82_cpx	2013	PWID	mSSDU12	KU820825
MM002	MM	82_cpx	2013	PWID	mSSDU191	KU820836
MM003	MM	82_cpx	2013	PWID	mSSDU195	KU820837
MM004	MM	82_cpx	2013	PWID	mSSDU63	KU820844
MM005	MM	82_cpx	2013	PWID	mSSDU75	KU820845
MM006	MM	83_cpx	2013	PWID	mSSDU118	KU820824
MM007	MM	83_cpx	2013	PWID	mSSDU137	KU820826
MM008	MM	83_cpx	2013	PWID	mSSDU144	KU820828
MM009	MM	83_cpx	2013	PWID	mSSDU151	KU820829
MM010	MM	83_cpx	2013	PWID	mSSDU178	KU820833
MM011	MM	83_cpx	2013	PWID	mSSDU180	KU820834
MM012	MM	83_cpx	2013	PWID	mSSDU21	KU820839
MM013	MM	83_cpx	2013	PWID	mSSDU24	KU820842
MM014	MM	83_cpx	2013	PWID	mSSDU94	KU820847
MY001	MY	51_01B	2009	N/A	09MYKL050	KJ485698
MY002	MY	58_01B	2011	PWID	11MY1ZK731	KC522032
MY003	MY	58_01B	2011	PWID	11MY1EP794	KC522034
PK001	PK	A1	2015	PWID	PK026	KX232618
PK002	PK	A1	2014	PWID	PK027	KX232619
PK003	PK	C	2014	SH	DEMC14PK009	KU749412
PK004	PK	C	2014	MSM	PK010	KX232603
SG001	SG	01_AE	2008	N/A	HM033	KY213722
SG002	SG	01_AE	2008	N/A	HM067	KY213726
SG003	SG	01_AE	2009	N/A	HM132F	KY213729
SG004	SG	01_AE	2008	N/A	HM018	KY213730
SG005	SG	51_01B	2011	SG	11SG_HM021	JN029801
SG006	SG	B	2008	N/A	HM024	KY213741
SG007	SG	B	2009	N/A	HM151	KY213744
TH001	TH	01_AE	1993	N/A	93TH051	AB220944
TH002	TH	01_AE	1993	N/A	93TH054	AB220945
TH003	TH	01_AE	1993	N/A	93TH060	AB220946
TH004	TH	01_AE	1993	N/A	93TH062	AB220947
TH005	TH	01_AE	1993	PWID	93TH065	AB220948
TH006	TH	01_AE	1993	PWID	93TH057	AB253424
TH007	TH	01_AE	1990	SH	CM235	AF259954
TH008	TH	01_AE	2001	PWID	OUR786I	AY358036
TH009	TH	01_AE	2002	PWID	OUR737I	AY358037
TH010	TH	01_AE	2001	PWID	OUR674I	AY358038
TH011	TH	01_AE	1999	PWID	OUR199I	AY358039
TH012	TH	01_AE	1999	PWID	OUR066I	AY358043
TH013	TH	01_AE	1999	PWID	OUR098I	AY358044
TH014	TH	01_AE	1999	PWID	OUR164I	AY358045

TH015	TH	01_AE	2000	PWID	OUR201I	AY358046
TH016	TH	01_AE	1999	PWID	OUR202I	AY358047
TH017	TH	01_AE	1999	PWID	OUR203I	AY358048
TH018	TH	01_AE	2002	PWID	OUR769I	AY358062
TH019	TH	01_AE	1999	N/A	OUR008I	AY358065
TH020	TH	01_AE	2000	PWID	OUR200I	AY358066
TH021	TH	01_AE	1992	SH	92TH001	AY494968
TH022	TH	01_AE	1990	N/A	90TH_CM244	AY713425
TH023	TH	01_AE	1990	SH	90TH_CM240	AY736838
TH024	TH	01_AE	2004	N/A	BKD	DQ314731
TH025	TH	01_AE	2004	N/A	BKM	DQ314732
TH026	TH	01_AE	2006	N/A	41CC1	EU743763
TH027	TH	01_AE	2006	N/A	41PB3	EU743764
TH028	TH	01_AE	2006	N/A	45CC1	EU743765
TH029	TH	01_AE	2006	N/A	45PB1	EU743766
TH030	TH	01_AE	2006	N/A	47CC11	EU743767
TH031	TH	01_AE	2006	N/A	47PL1	EU743768
TH032	TH	01_AE	2006	N/A	52PB3	EU743771
TH033	TH	01_AE	2006	N/A	52PL4	EU743772
TH034	TH	01_AE	2006	N/A	52PL7	EU743773
TH035	TH	01_AE	2006	N/A	60PB2	EU743776
TH036	TH	01_AE	2006	N/A	60PL2	EU743777
TH037	TH	01_AE	2006	N/A	65CC1	EU743779
TH038	TH	01_AE	2006	N/A	65CC4	EU743780
TH039	TH	01_AE	2006	N/A	98CC2	EU743782
TH040	TH	01_AE	2006	N/A	98CC3	EU743783
TH041	TH	01_AE	2006	N/A	99CC8	EU743785
TH042	TH	01_AE	2006	N/A	99PB2	EU743786
TH043	TH	01_AE	2006	N/A	105PB1	EU743791
TH044	TH	01_AE	2006	N/A	105PL3	EU743793
TH045	TH	01_AE	2005	SH	703357_c02	JN944658
TH046	TH	01_AE	1990	SH	CM246_c1	JN944663
TH047	TH	01_AE	2008	N/A	AA007a_WG1	JX446755
TH048	TH	01_AE	2005	N/A	AA051a01R	JX447294
TH049	TH	01_AE	2006	N/A	AA100b07R	JX447902
TH050	TH	01_AE	N/A	N/A	107747_048	JX848346
TH051	TH	01_AE	N/A	N/A	113035_007	JX848348
TH052	TH	01_AE	2009	N/A	RTA2-env	KF268035
TH053	TH	01_AE	2009	N/A	RTA4-env	KF268037
TH054	TH	01_AE	2009	N/A	RTA5-env	KF268038
TH055	TH	01_AE	2009	N/A	RTA6-env	KF268039
TH056	TH	01_AE	2009	N/A	RTA8-env	KF268040
TH057	TH	01_AE	2009	N/A	RTA9-env	KF268041
TH058	TH	01_AE	2009	N/A	RTA11-env	KF268042
TH059	TH	01_AE	2009	N/A	RTA21-env	KF268045
TH060	TH	01_AE	2009	N/A	RTA23-env	KF268046
TH061	TH	01_AE	2009	N/A	RTA24-env	KF268047
TH062	TH	01_AE	2009	N/A	RTA27-env	KF268048
TH063	TH	01_AE	2000	N/A	3002B01.A12	KJ952242
TH064	TH	01_AE	N/A	N/A	3017F5.A1	KJ952314
TH065	TH	01_AE	2000	N/A	3090A02.C6	KJ952765
TH066	TH	01_AE	2000	N/A	3111A02.A2	KJ952897
TH067	TH	01_AE	2000	N/A	3112A02.A1	KJ952917
TH068	TH	01_AE	N/A	N/A	3141A16.B2	KJ953175
TH069	TH	01_AE	1999	N/A	3151A16.A2	KJ953229
TH070	TH	01_AE	1999	N/A	3184A08.B1	KJ953408
TH071	TH	01_AE	1999	N/A	3210A08.D22	KJ953596
TH072	TH	01_AE	1999	N/A	3212A10.B10	KJ953611
TH073	TH	01_AE	2000	N/A	3219A08.D1	KJ953664
TH074	TH	01_AE	1992	SH	TH023	KU562843
TH075	TH	01_AE	2013	MSM	2544233P000FL_Sa	MG989508
TH076	TH	01_AE	2010	MSM	2545374P000FL_Sj	MG989517
TH077	TH	01_AE	2015	MSM	2547188P000FL_Sk	MG989534
TH078	TH	01_AE	2015	MSM	2548453P000FL_Sa	MG989553
TH079	TH	01_AE	2009	MSM	2545573P000R_Sd	MG989591
TH080	TH	01_AE	2009	MSM	2545577P000R_Sd	MG989592

TH081	TH	01_AE	2009	MSM	2547933P000R_Sa	MG989606
TH082	TH	01_AE	2010	MSM	2549196P000R_Sn	MG989615
TH083	TH	01B	2001	PWID	OUR033I	AY358071
TH084	TH	01B	2002	PWID	OUR846I	AY358072
TH085	TH	01B	2006	N/A	40PB1	EU743761
TH086	TH	01B	2006	N/A	40PB3	EU743762
TH087	TH	01B	2005	N/A	AA095a_WG16	JX447827
TH088	TH	01B	2007	N/A	AA096a_WG1	JX447833
TH089	TH	01B	2013	MSM	2543499P000FL_Sc	MG989494
TH090	TH	01B	2015	MSM	2544032P000FL_Sa	MG989505
TH091	TH	34_01B	1999	PWID	OUR1969P	EF165539
TH092	TH	34_01B	1999	PWID	OUR2478P	EF165541
TH093	TH	B	2005	N/A	T286588_sga01	JF297222
TH094	TH	B	2005	N/A	05TH356764	JN248344
TH095	TH	B	2005	N/A	05TH356883	JN248345
TH096	TH	B	2005	N/A	T286588_01	KC749001
TH097	TH	B	2010	MSM	DEMB10TH002	KP109514
TH098	TH	B	2010	N/A	40353v04_01R	KU230423
TW001	TW	07_BC	2004	PWID	TW_D3	DQ230841
TW002	TW	07B	2004	PWID	TW_D60	DQ230842
VN001	VN	01_AE	1998	N/A	98VNBG4	FJ185228
VN002	VN	01_AE	1998	N/A	98VNBG5	FJ185229
VN003	VN	01_AE	1998	N/A	98VNBG6	FJ185230
VN004	VN	01_AE	1998	N/A	98VNBG7	FJ185231
VN005	VN	01_AE	1998	N/A	98VNHD9	FJ185232
VN006	VN	01_AE	1998	N/A	98VNHD10	FJ185233
VN007	VN	01_AE	1998	N/A	98VNHD11	FJ185234
VN008	VN	01_AE	1998	N/A	98VNND17	FJ185236
VN009	VN	01_AE	1997	N/A	97VNHCM302	FJ185238
VN010	VN	01_AE	1997	N/A	97VNAG216	FJ185254
VN011	VN	01_AE	1997	N/A	97VNAG220	FJ185256
VN012	VN	01_AE	1997	N/A	97VNAG221	FJ185257
